# Supplementary material for: Deep learning-driven adaptive optics for single-molecule localization microscopy
Source: Nat Methods. 2023 Sep 28;20(11):1748–58. doi: 10.1038/s41592-023-02029-0 (PMC10630144; doi:10.1038/s41592-023-02029-0)
Supplement: Supplementary file 1 — Supplementary Figs. 1–9, Supplementary Videos 1–12, Supplementary Tables 1–4 and Supplementary Notes 1–9. [file 41592_2023_2029_MOESM1_ESM.pdf]

nature methods

Article

https://doi.org/10.1038/s41592-023-02029-0

Deep learning-driven adaptive optics for single-molecule localization microscopy

In the format provided by the authors and unedited

Table of Content

Supplementary Fig. 1: Comparison between measured PSFs and PSFs simulated from network estimations. 4

Supplementary Fig. 2: Sub-regions from raw blinking frames and network estimation per sub-region during DL-AO compensation. 6

Supplementary Fig. 3: Repeated tests of DL-AO. 8

Supplementary Fig. 4: Comparison between DL-AO and metric-based AO on compensating sample induced distortion. 10

Supplementary Fig. 5: Comparison between DL-AO and metric-based AO in simulation. 11

Supplementary Fig. 6: 12

Supplementary Fig. 7: DL-AO compensates for random and sudden wavefront changes during continuous SMLM acquisition. 14

Supplementary Fig. 8: Resolution measurements of super resolution images acquired with DL-AO. 15

Supplementary Fig. 9: Examples of observed wavefront variations at four different segments of the imaging area. 16

Supplementary Video 1: Single molecule blinking frames during deep learning driven adaptive optics (DL-AO) for SMLM, when compensating artificially induced aberrations. 17

Supplementary Video 2: Single molecule blinking frames during deep learning driven adaptive optics (DL-AO) for SMLM, when compensating aberrations induced by refractive index mismatch. 17

Supplementary Video 3: Single molecule blinking frames during deep learning driven adaptive optics (DL-AO) for SMLM, when compensating aberrations induced by inhomogeneous refractive indices of 101 μm mouse brain tissue. 18

Supplementary Video 4: Compensation speed comparison between metric-based AO and DL-AO. 18

Supplementary Video 5: Comparison between metric-based AO and DL-AO on compensating aberrations when PSFs are out-of-focus 18

Supplementary Video 6-7: DL-AO compensates dynamic aberrations in real time 19

Supplementary Video 8: Comparison between metric-based AO and DL-AO on compensating aberration through dynamic structure changes 19

Supplementary Video 9: Single molecule blinking frames acquired with and without DL-AO when imaging through brain sections. 20

Supplementary Video 10-11: Single molecule blinking frames acquired when DL-AO is compensating artificially induced aberrations in Astigmatism-based setup. 20

Supplementary Video 12: Single molecule blinking frames when DL-AO is compensating aberrations induced by refractive index mismatch in Astigmatism-based setup. 21

Supplementary Table 1. Imaging parameters for experimental data 22

Supplementary Table 2. Detailed sizes in each layer of the network architecture......23
Supplementary Table 3. Computation Time for compensation with DL-AO......24
Supplementary Table 4. Variation range of parameters in training data generation......25
Supplementary Notes......26
1. Comparison between DL-AO and metric-based AO......26
1.1 Comparison between DL-AO and metric-based AO in concept......26
1.2 Comparison between DL-AO and metric-based AO in practice......28
2. Workflow of deep learning driven AO......33
2.1 General workflow of SMLM imaging with DL-AO......33
2.2 Segmentation process to obtain sub-regions......34
2.3 Aberration estimation with deep neural network......35
2.4 Combining estimation with Kalman filter......36
2.5 Switching neural networks for estimation......38
2.5.1 Reasons of switching networks......39
2.5.2 Detailed process in switching networks......41
3. Considerations in mirror mode generation......42
3.1 Reasons of using mirror mode......42
3.2 Mirror modes generation process......42
3.3 Measurements of experimental mirror modes......45
4. Training data generation......47
4.1 Measurement of pupil functions under instrument optimum......48
4.2 Simulating PSFs with wavefront distortions......49
5. Kalman filter......51
5.1 General concept of Kalman filter......51
5.2 Kalman filter implementation for deep learning driven AO......52
6. PSFs and pupil functions used for characterizing DL-AO performance......56
6.1 PSF measurements for *in vitro* PSF models......56
6.2 in vitro PSF models and pupil functions......57
6.2.1 Phase retrieval process......57
6.2.2 *in vitro* PSF models and pupil functions......59
6.3 *in situ* PSF models and pupil functions......59
7. DL-AO for Astigmatism-based setup......60
7.1 Modifications to adapt DL-AO for Astigmatism-based setup......60
7.1.1 Modifications in Optical Setup......60
7.1.2 Modifications in Network Architecture......61

7.1.3 Modifications in Training Data Generation 61
7.1.4 Modifications in DL-AO workflow 62
7.2 Response accuracy of DL-AO network for Astigmatism-based setup 64
7.3 Restoring Astigmatism PSFs with DL-AO 71
7.4 Robustness of DL-AO for Astigmatism-based setup 76
7.5 Discussion about adapting DL-AO for other 3D imaging modalities. 80
8. Investigation on controlling 50 modes simultaneously with DL-AO 80
8.1 Modifications in DL-AO for controlling 50 modes simultaneously. 80
8.2 Response accuracy of DL-AO network to 50 mirror modes changes 85
8.3 Restoring PSFs using 50 modes 90
9. Discussion about imaging tissue sections 92
9.1 Challenges in comparing resolution among super resolution techniques 92
9.2 Challenges in whole slice reconstruction and vision for future development 93
References 98

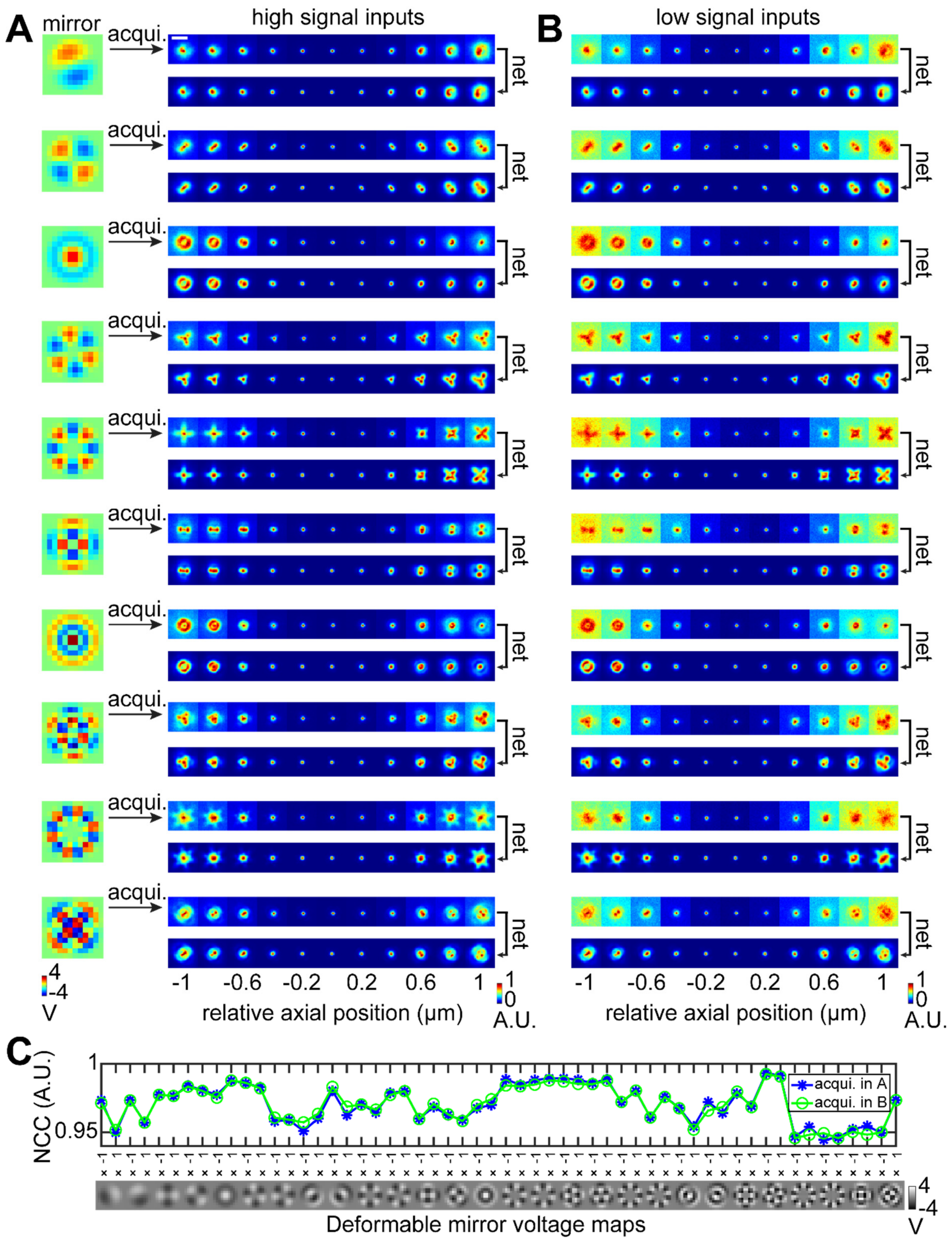

Supplementary Fig. 1: Comparison between measured PSFs and PSFs simulated from network estimations. (**A**) Comparison between the measured PSFs that are input to the trained network and PSFs simulated from the corresponding outputs from the network. The

arrows under “acqui.” point to PSF stacks measured under a specific deformable mirror voltage map. These measured PSFs are input to the network for estimating aberrations. After obtaining the estimated wavefronts from the network, we simulated PSF stacks based on this estimation, which can be referred to as the network-estimated PSFs. We displayed the network estimated PSFs under the corresponding measured input PSFs to visually demonstrate the one-time inference accuracy with the DL-AO network. Network estimations were obtained by inputting 31 biplane sub-regions, each of which contains a single PSF measured from 100-nm-diameter crimson bead, then averaging among the 31 outputs. The 31 sub-regions were measured by moving Piezo stage from -1.5 μm to 1.5 μm around the focus of the first detection plane, with 0.1 μm step size. Only PSFs from first detection plane were shown in this figure for comparison. PSFs were simulated without background and noise for visualization. Scale bar: 2 μm. ‘acqui.’ stands for acquisition. ‘net’ stands for network estimation. (**B**) A repeat of what is shown in A with relatively lower signal level in measured PSFs. (**C**) Quantitative comparisons between measured PSFs under different mirror voltage maps and PSFs simulated from network estimations w.r.t. the measured PSFs. The similarities between measured PSFs and simulated PSFs were quantified using 3D normalized cross correlation (NCC). Acquisition in A has relatively higher signal level comparing to acquisition in B. The NCC values were calculated between PSFs simulated from network estimations (w.r.t. measurements under either higher signal or lower signal) and the PSFs measured under higher signal level as examples shown in A.

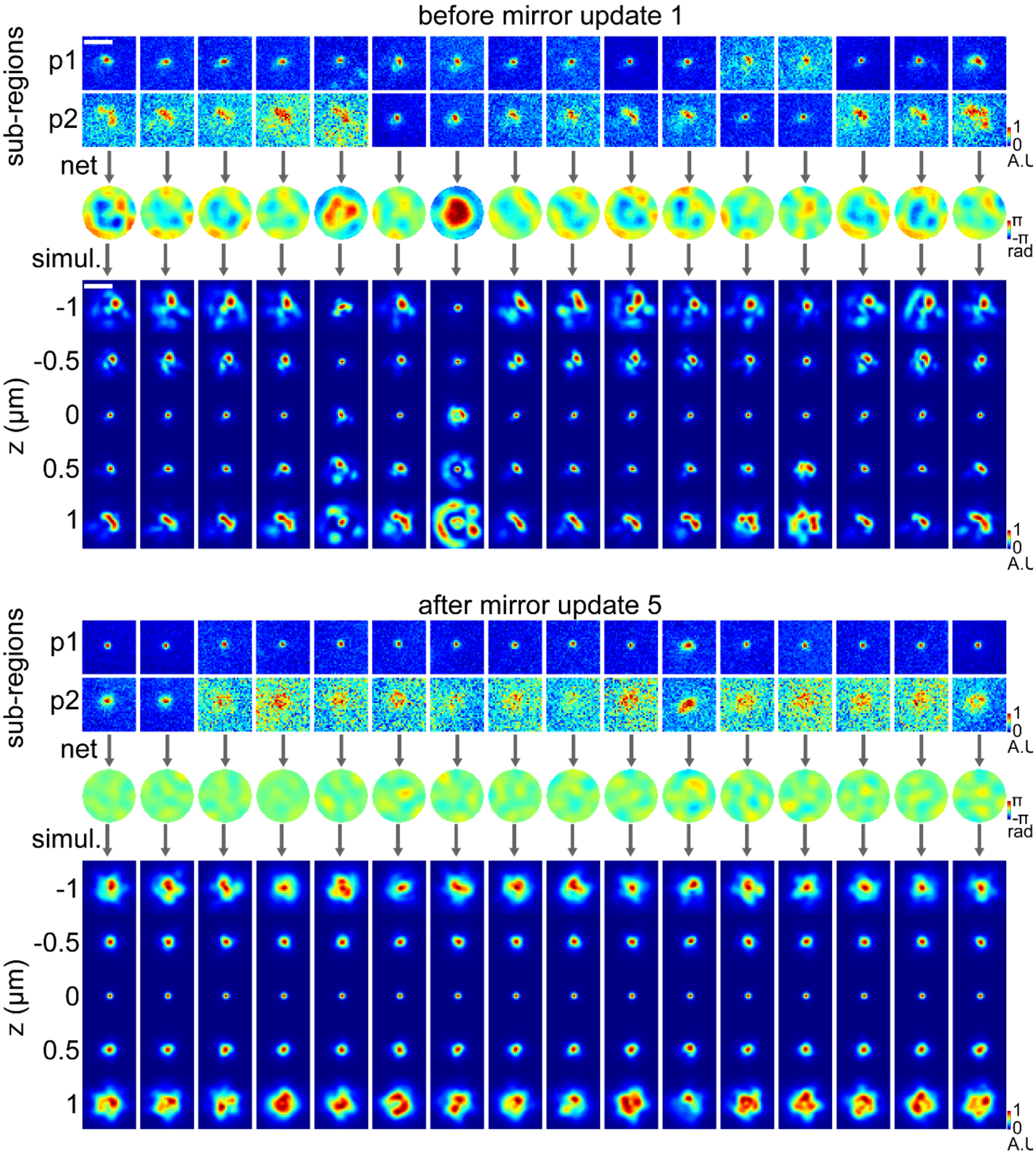

Supplementary Fig. 2: Sub-regions from raw blinking frames and network estimation per sub-region during DL-AO compensation. The sub-regions shown in this figure are all the data used for network estimation before mirror update 1 and after mirror update 5 during DL-AO compensation. ‘p1’ stands for detection plane 1 and ‘p2’ stands for detection plane 2. The SMLM blinking frames for compensation were acquired from immune-fluorescence-labeled Tom20 specimen. Network outputs a vector of mirror mode coefficients for each sub-region. The wavefronts below each sub-region were obtained by linear combining measured mirror modes with output coefficients from neural network. The wavefronts were then used to simulate PSFs at different axial positions to check the similarity between measured PSFs and PSFs

simulated from network estimations. PSFs were simulated without background and noise for visualization. The corresponding PSFs measured from 100-nm-diameter crimson beads nearby this compensation area post each mirror update were shown in **Extended Data Fig. 8A**. Scale bars: 2 μm.

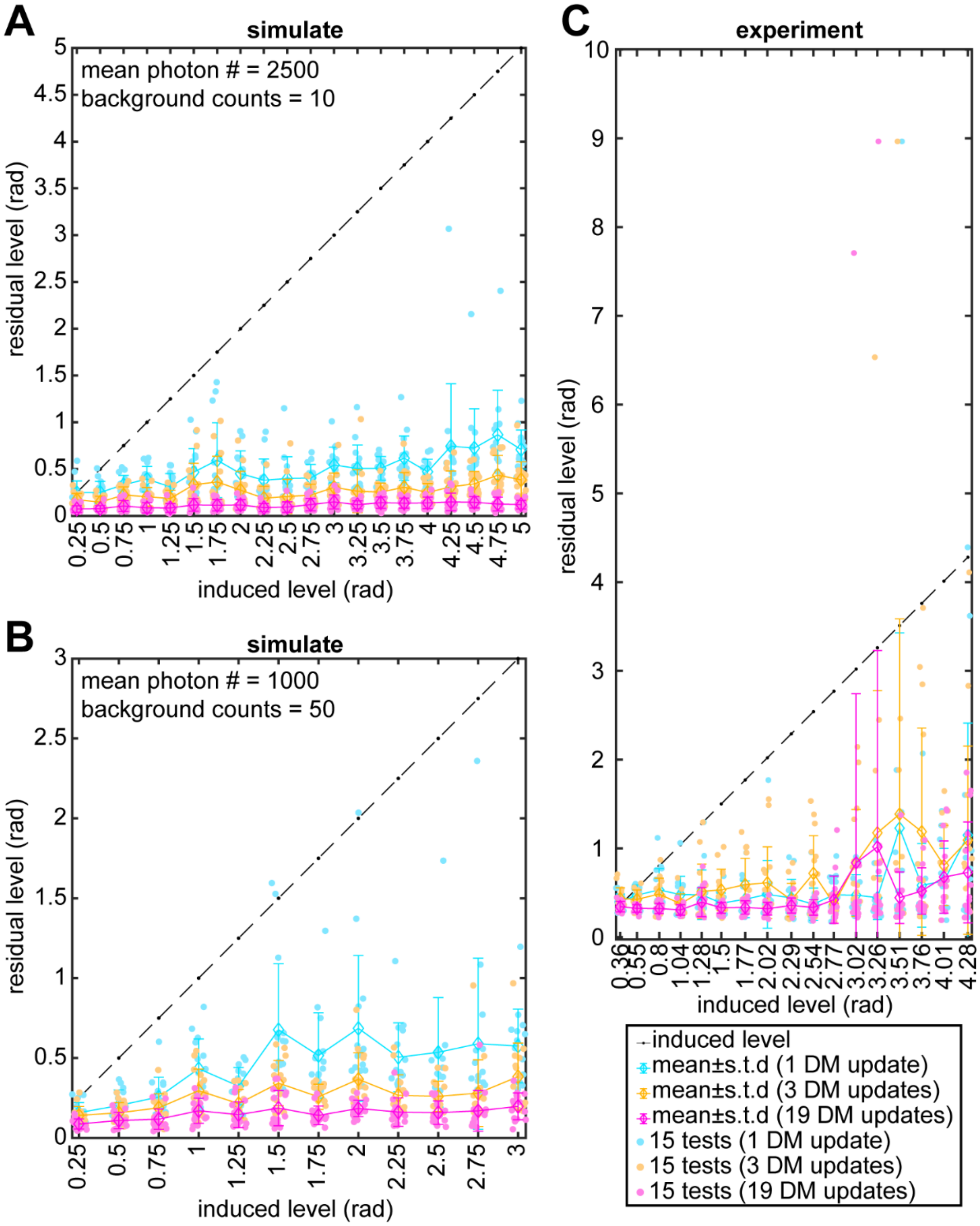

Supplementary Fig. 3: Repeated tests of DL-AO. (**A**, **B**) Summary of repeated tests of DL-AO for compensating aberrations of different levels (in W_{rms}) based on simulated SMLM blinking data. Each simulated SMLM frames contain 128×128 pixels, with pixel size of 119 nm. Number of PSFs per frame were generated from Poisson distribution with a mean of 13. Axial positions of molecules were generated from uniform distribution from -1 to 1 μm range. The number of photon counts in each PSF was generated from exponential distribution with mean equal to 2500 and 1000 for A and B respectively. The number of background photon counts in each frame was set to be 10 and 50 for A and B respectively. (**C**) Summary of repeated tests

of DL-AO for compensating aberrations in different levels (in W_{rms}) based on experimental blinking frames from immune-fluorescence-labeled Tom20 specimen.

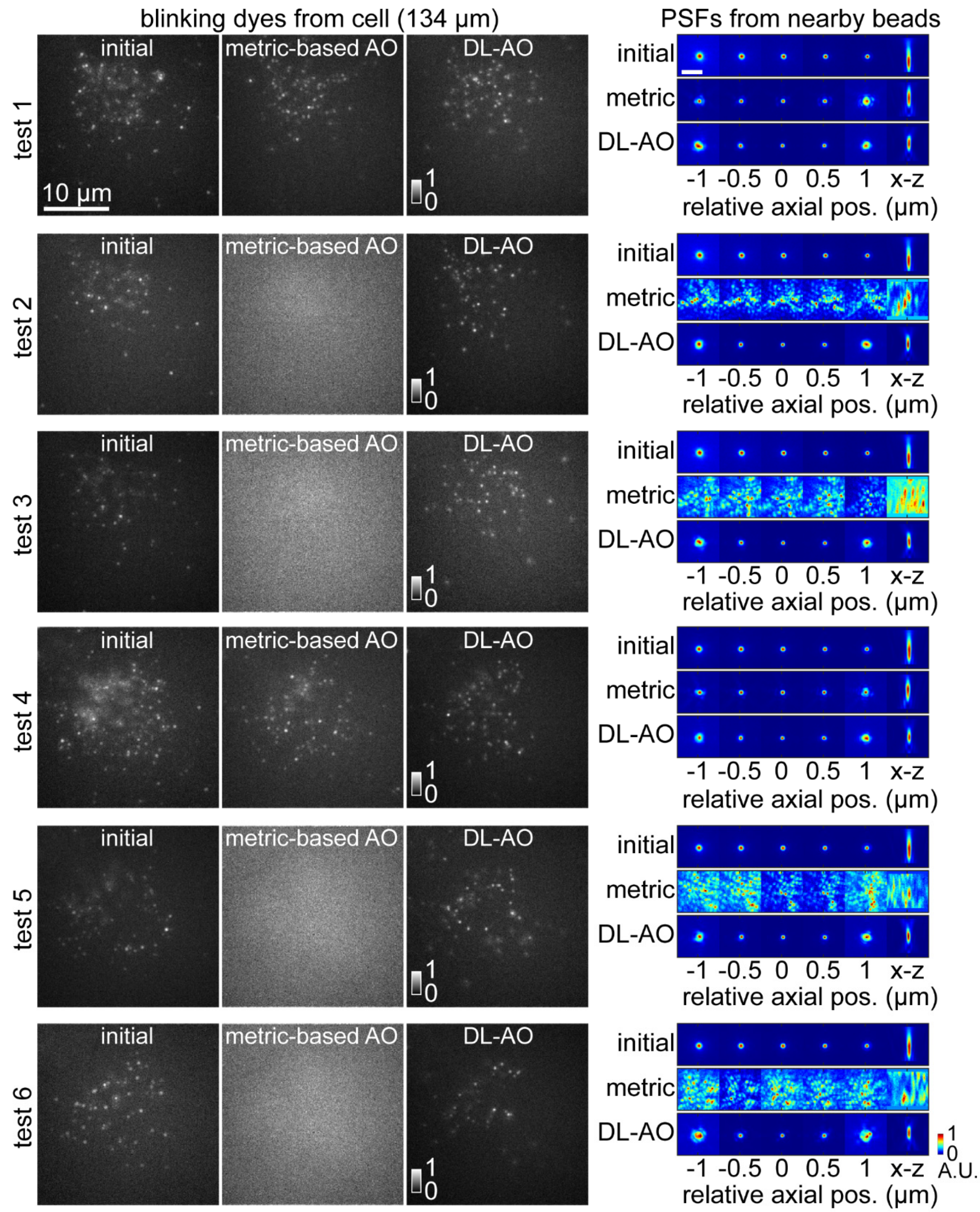

Supplementary Fig. 4: Comparison between DL-AO and metric-based AO on compensating sample induced distortion. The SMLM blinking frames for compensation were acquired from immune-fluorescence-labeled Tom20 specimen at 134 μm from bottom coverslip surface in water-based media (n = 1.35). PSFs were measured from 100-nm-diameter crimson beads nearby the compensation area post SMLM acquisition. Scale bar: 2 μm. The results shown are representatives of 9 tests.

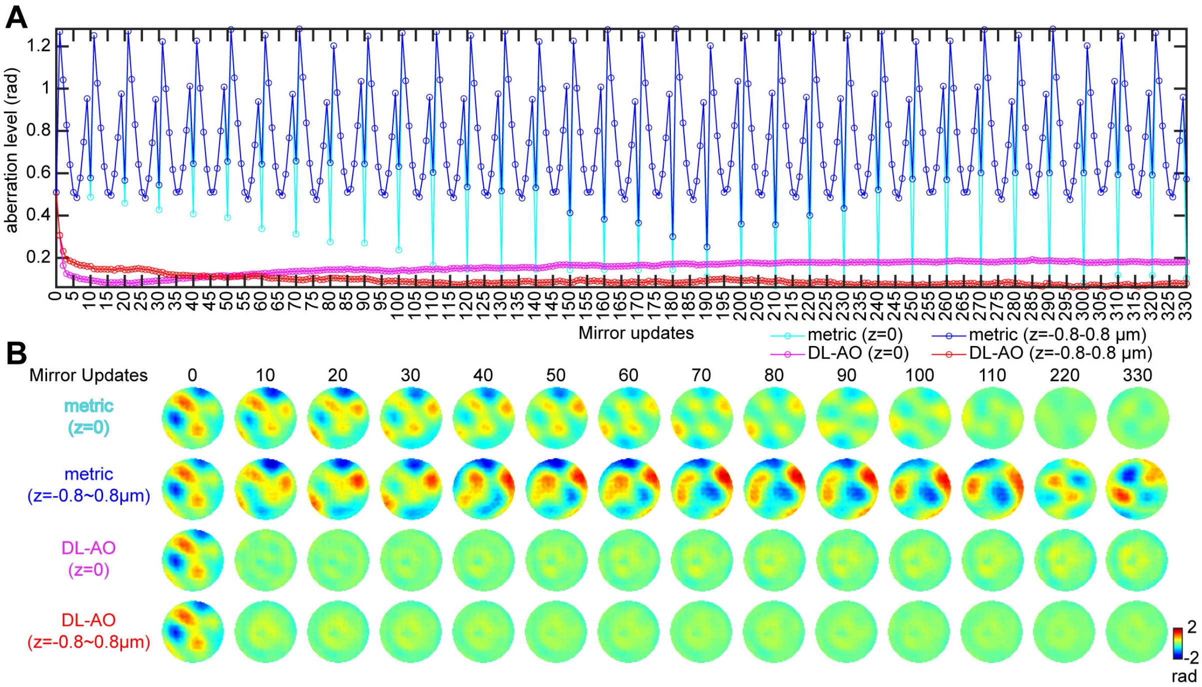

Supplementary Fig. 5: Comparison between DL-AO and metric-based AO in simulation. Each simulated SMLM frames contain 128×128 pixels, with pixel size of 119 nm. Number of PSFs per frame were generated from Poisson distribution with a mean of 13. Axial positions of molecules were set to be 0, or generated from uniform distribution from -0.8 to 0.8 μm range. The number of photon counts in each PSF was generated from exponential distribution with mean equal to 2500. The number of background photon counts in each frame was set to be 20. To compare DL-AO with metric-based AO with same initial aberration and same compensation mode, we chose measured mirror modes 3, 4, 1, 2, 6, 7, 5, 12, 13, 10, 11 (which resemble similar shape of Zernike Polynomials used in metric-based AO) to simulate PSFs containing aberration (**Supplementary Note 3**). The initial wavefront has distortion level of 0.5 radian in W_{rms}. We used the optimal setting suggested in metric-based AO for compensation: maximum bias of 1 radian, 9 biases and 3 rounds.

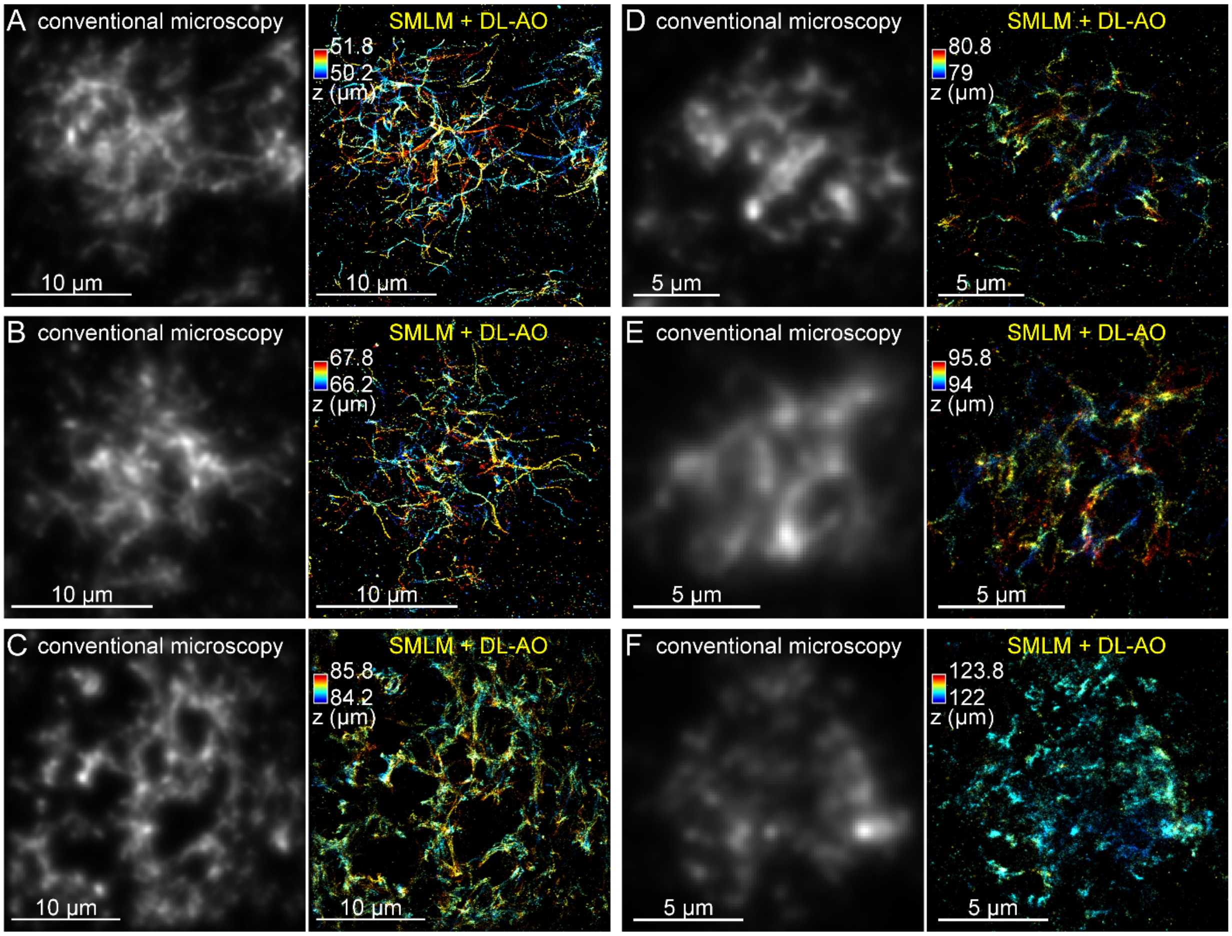

Supplementary Fig. 6: Comparison between images obtained from conventional diffraction-limited microscopy and 3D SMLM reconstruction with DL-AO (**A-F**) Representative of six datasets of Immune-fluorescence-labeled amyloid-β fibrils in 125-µm-cut brain sections of 7.5-month-old 5XFAD female mouse. Color code indicates axial positions of single molecules. Conventional microscopy cannot resolve the 3D organizations of amyloid-β fibrils, e.g. bundle-like structures of amyloid-β fibrils. Conventional diffraction-limited images are generated by replacing single molecule localization points with their corresponding PSFs without aberration.

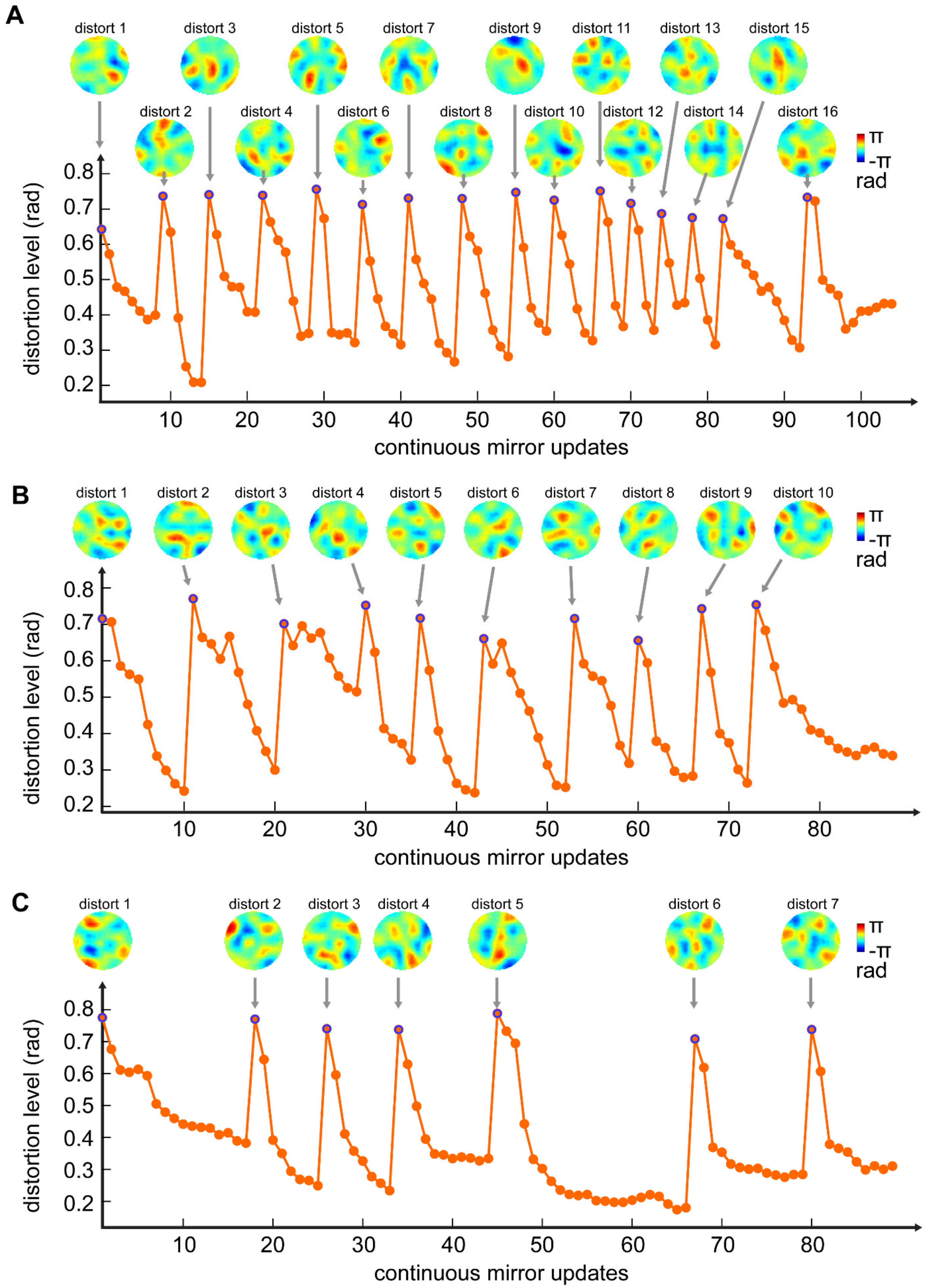
A
distort 1
distort 2
distort 3
distort 4
distort 5
distort 6
distort 7
distort 8
distort 9
distort 10
distort 11
distort 12
distort 13
distort 14
distort 15
distort 16
π
-π
rad
distortion level (rad)
continuous mirror updates
B
distort 1
distort 2
distort 3
distort 4
distort 5
distort 6
distort 7
distort 8
distort 9
distort 10
π
-π
rad
distortion level (rad)
continuous mirror updates
C
distort 1
distort 2
distort 3
distort 4
distort 5
distort 6
distort 7
π
-π
rad
distortion level (rad)
continuous mirror updates

Supplementary Fig. 7: DL-AO compensates for random and sudden wavefront changes during continuous SMLM acquisition. Images in the top row of A-C are the distorted wavefronts introduced during continuous imaging. A dot with a blue circle corresponds to a mirror update that introduces a random wavefront distortion (targeted level of 0.75 rad in W_{rms}). Each grey arrow points from an induced wavefront distortion to its corresponding mirror update. The dots without blue circles correspond to mirror updates driven by deep neural network. The single molecule blinking frames with random and sudden wavefront changes were continuously acquired for four, four, and three minutes for A, B, C, respectively. The compensations were performed based on blinking data from specimens of immune-fluorescence-labeled Tom20 in COS-7 cells. 50 camera frames (with 50 Hz frame rate) were used for DL-AO estimation before each mirror update.

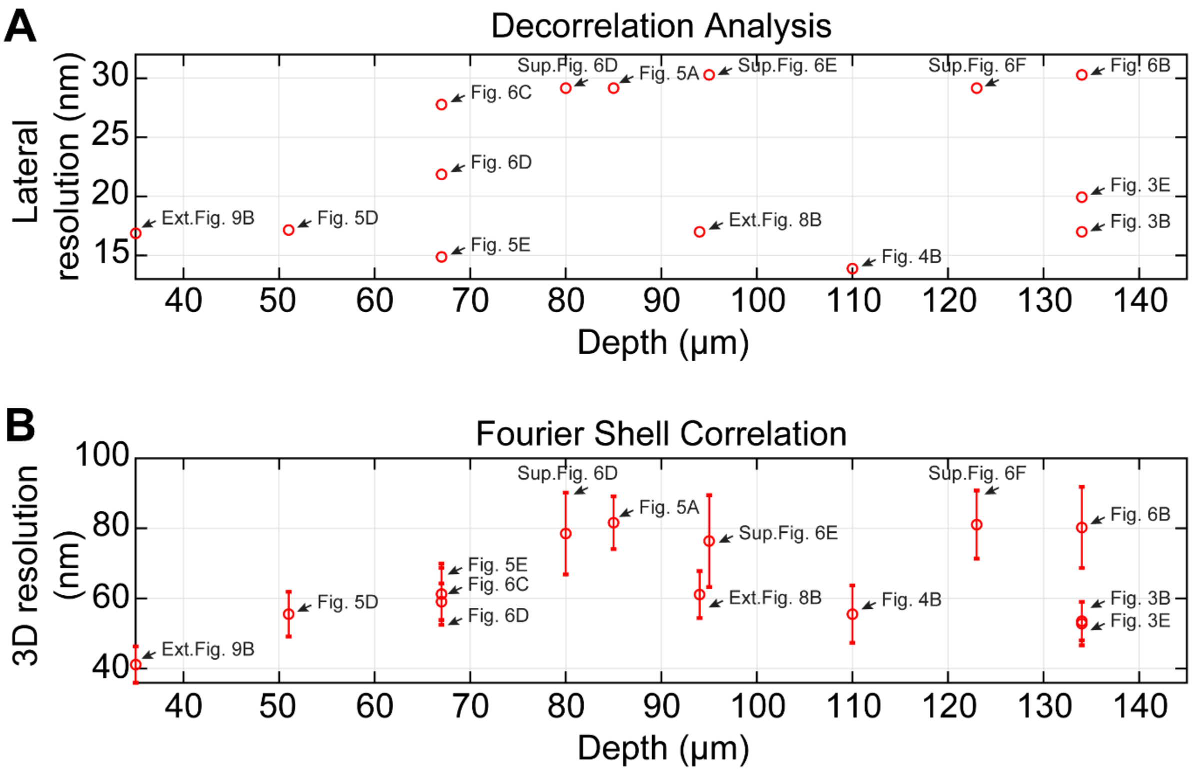

Supplementary Fig. 8: Resolution measurements of super resolution images acquired with DL-AO. (**A**) Lateral resolution measured with decorrelation analysis[1]. The resolution measured by decorrelation: 16.87 nm for Extended Data Fig. 9B, 17.15 nm for Fig. 5D, 14.87 nm for Fig. 5E, 27.77 nm for Fig. 6C, 21.85 nm for Fig. 6D, 29.16 nm for Supplementary Fig. 6D and Fig. 5A, 17.00 nm for Extended Data Fig. 8B, 30.27 nm for Supplementary Fig. 6E, 13.88 nm for Fig. 4B, 29.16nm for Supplementary Fig. 6F, 17.00 nm for Fig. 3B, 19.93 nm for Fig. 3E, 30.27 nm for Fig. 6B. (**B**) 3D resolution measured with Fourier Shell Correlation (FSC) analysis[2]. The images were segmented into 49 regions and FSC was calculated for each region separately due to the limited computation resources. The mean and standard deviation of the resolution measured from 49 regions are: 41.10 ± 5.17 nm for Extended Data Fig. 9B, 55.52 ± 6.40 nm for Fig. 5D, 61.25 ± 7.48 nm for Fig. 5E, 61.22 ± 8.73 nm for Fig. 6C, 59.05 ± 5.20 nm for Fig. 6D, 78.50 ± 11.65 nm for Supplementary Fig. 6D, 81.60 ± 7.51 nm for Fig. 5A, 61.10 ± 6.69 nm for Extended Data Fig. 8B, 76.37 ± 13.10 nm for Supplementary Fig. 6E, 55.50 ± 8.19 nm for Fig. 4B, 81.04 ± 9.70 nm for Supplementary Fig. 6F, 53.50 ± 5.46 nm for Fig. 3B, 52.79 ± 6.19 nm for Fig. 3E, 80.22 ± 11.58 nm for Fig. 6B. Image reconstruction without AO is not included as a comparison here, because the image artifacts cannot be reflected by resolution measurements. ‘Ext.Fig.’ stands for ‘Extended Data Figure’.

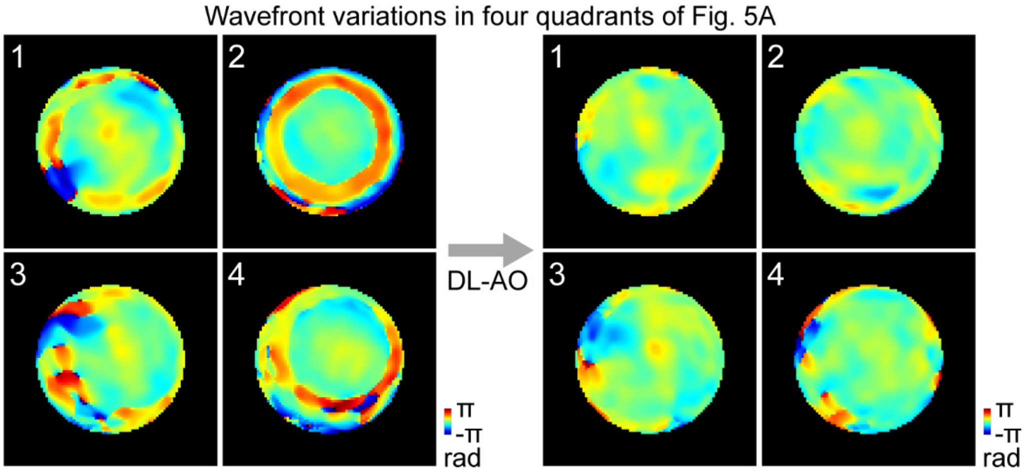

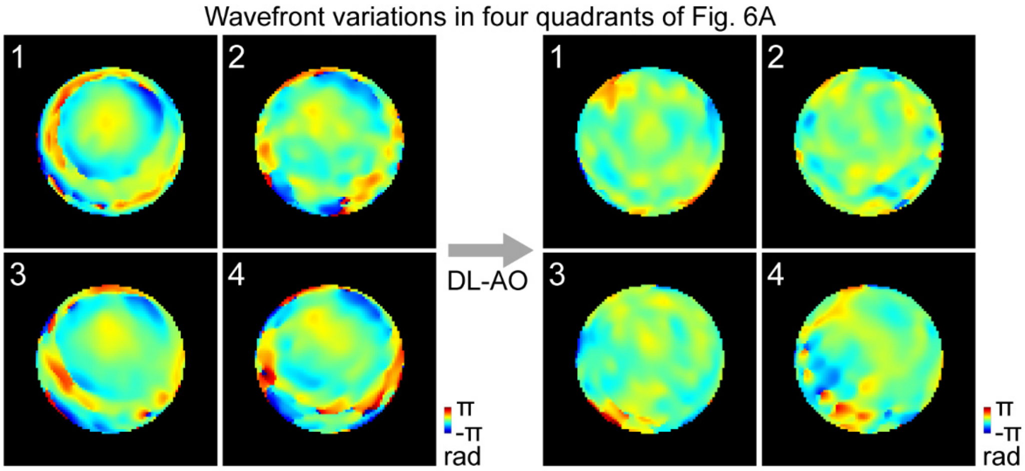

Supplementary Fig. 9: Examples of observed wavefront variations at four different segments of the imaging area. The region-specific wavefronts were calculated at each segment of our field-of-view with INSPR[3]. The left column shows wavefronts retrieved in dataset acquired without DL-AO. The right column shows wavefronts retrieved in dataset acquired with DL-AO. The two examples correspond to reconstructions in **Fig. 5A** and **Fig. 6A** respectively.

Supplementary Video 1: Single molecule blinking frames during deep learning driven adaptive optics (DL-AO) for SMLM, when compensating artificially induced aberrations.

Compensations are performed in real time during SMLM experiments when imaging immune-fluorescence-labeled Tom20 specimen. The left two panels are the raw blinking frames (after converting the analog-to-digital unit readings in camera frames to the effective photoelectrons, referred as photon #) captured from the two detection planes of a biplane setup. The displayed frame rate is set to be the same as the acquisition frame rate, which is 50 Hz. The top right panel shows the deformable mirror voltage map w.r.t current detection. The deformable mirror was updated every 50 camera frames. The amount of mirror update proposed by DL-AO will be displayed on the bottom right panel every 50 camera frames.

Supplementary Video 2: Single molecule blinking frames during deep learning driven adaptive optics (DL-AO) for SMLM, when compensating aberrations induced by refractive index mismatch.

Compensations are performed in real time during SMLM experiments when imaging immune-fluorescence-labeled Tom20 specimen. The specimen locates at 134 µm (**Methods**) with refractive indices of sample media and immersion oil being 1.35 and 1.406 respectively measured by Abbe refractometer (334610, Thermo Scientific). The left two panels are the raw blinking frames (after converting the analog-to-digital unit readings in camera frames to the effective photoelectrons, referred as photon #) captured from the two detection planes of a biplane setup. The displayed frame rate is set to be the same as the acquisition frame rate, which is 50 Hz. The top right panel shows the deformable mirror voltage map w.r.t current detection. The deformable mirror was updated every 30 camera frames. The amount of mirror update proposed by DL-AO will be displayed on the bottom right panel every 30 camera frames. Apparent focal planes were adjusted by moving the sample stage during compensation, since index mismatch induced aberration causes an apparent focal plane shift[4].

Supplementary Video 3: Single molecule blinking frames during deep learning driven adaptive optics (DL-AO) for SMLM, when compensating aberrations induced by inhomogeneous refractive indices of 101 µm mouse brain tissue.

Compensations are performed in real time during SMLM experiments when imaging immune-fluorescence-labeled amyloid-β fibrils of 7.5-month-old 5XFAD female mouse. The left two panels are the raw blinking frames (after converting the analog-to-digital unit readings in camera frames to the effective photoelectrons, referred as photon #) captured from the two detection planes of a biplane setup. The displayed frame rate is set to be the same as the acquisition frame rate, which is 50 Hz. The top right panel shows the deformable mirror voltage map w.r.t current detection. The deformable mirror was updated every 100 camera frames. The amount of mirror update proposed by DL-AO will be displayed on the bottom right panel every 100 camera frames.

Supplementary Video 4: Compensation speed comparison between metric-based AO and DL-AO.

Compensations are performed with in-focus PSFs from 100-nm-fluorescent beads. The left and right panel shows the raw data during the metric-based AO compensation and DL-AO compensation, respectively. The timestamp of each camera frame during imaging with AO is displayed on the top right corner of each panel. The bottom left panel shows the deformable mirror voltage map w.r.t current detection. The deformable mirror was updated every one camera frame. The grey levels indicate the photon counts per pixel. The time stamps were obtained from recorded files in each mirror updating cycle.

Supplementary Video 5: Comparison between metric-based AO and DL-AO on compensating aberrations when PSFs are out-of-focus

Compensations are performed with 100-nm-fluorescent beads that are slightly out-of-focus. The left and right panel shows the raw data during the metric-based AO compensation and DL-AO compensation, respectively. The timestamp of each camera frame during imaging with AO is

displayed on the top right corner of each panel. The bottom left panel shows the deformable mirror voltage map w.r.t current detection. The deformable mirror was updated every one camera frame. The grey levels indicate the photon counts per pixel. The time stamps were obtained from recorded files in each mirror updating cycle.

Supplementary Video 6-7: DL-AO compensates dynamic aberrations in real time

Compensations are performed in real time during SMLM experiments when imaging immune-fluorescence-labeled Tom20 in COS-7 cells. The two panels in the upper row are the raw blinking frames (after converting the analog-to-digital unit readings in camera frames to the effective photoelectrons, referred as photon #) captured from the two detection planes of a biplane setup. The wavefront shape w.r.t current detection is shown at the bottom left corner of the upper left panel. The deformable mirror was updated every 50 camera frames. The displayed frame rate is set to be the same as the acquisition frame rate, which is 50 Hz. The number of mirror updates proposed by DL-AO after each distortion will be displayed on the bottom left panel. The plot on the bottom row shows the distortion level changes after each mirror update. A dot with a blue circle corresponds to a mirror update that introduces a random wavefront distortion (targeted level of 0.75 rad in W_{rms}). The dots without blue circles correspond to mirror updates driven by deep neural network. The single molecule blinking frames with random and sudden wavefront changes were continuously acquired for three and four minutes from the immune-fluorescence-labeled Tom20 in COS-7 cells for Supplementary Video 6 and 7, respectively.

Supplementary Video 8: Comparison between metric-based AO and DL-AO on compensating aberration through dynamic structure changes

The two panels are the raw blinking frames (after converting the analog-to-digital unit readings in camera frames to the effective photoelectrons, referred as photon #) captured during metric-based AO and DL-AO, respectively. Compensations are performed in real-time during SMLM experiments when imaging immune-fluorescence-labeled Tom20 in COS-7 cells. The bottom left

panel shows the deformable mirror voltage map w.r.t current detection. The grey levels indicate the photon counts per pixel. The displayed frame rate is set to be the same as the acquisition frame rate, which is 50 Hz. The deformable mirror was updated every 10 camera frames when using DL-AO, while the specimen was shifted 10 nm in between each adjacent frame by the nano-positioning system (Nano-LP200, Mad City Labs) to introduce structural changes before DL-AO propose each mirror update.

Supplementary Video 9: Single molecule blinking frames acquired with and without DL-AO when imaging through brain sections.

The two panels are the raw blinking frames (after converting the analog-to-digital unit readings in camera frames to the effective photoelectrons, referred as photon #) captured without and with DL-AO, respectively. The grey levels indicate the photon counts per pixel. The acquisition frame rate is 50 Hz. Twenty-two different imaging areas were shown in this video. These raw data were acquired from the following specimens: immune-fluorescence-labeled amyloid-β plaques in mouse brain sections, immune-fluorescence-labeled Thy1-ChR2-EYFP in mouse brain sections, and Tom20 proteins in COS-7 cells placed on top of unlabeled mouse brain sections.

Supplementary Video 10-11: Single molecule blinking frames acquired when DL-AO is compensating artificially induced aberrations in Astigmatism-based setup.

Compensations are performed in real time during SMLM experiments when imaging immune-fluorescence-labeled Tom20 specimen. The left panel shows the raw blinking frames (after converting the analog-to-digital unit readings in camera frames to the effective photoelectrons, referred as photon #). The displayed frame rate is set to be the same as the acquisition frame rate, which is 50 Hz. The top right panel shows the deformable mirror voltage map w.r.t current detection. The deformable mirror was updated every 100 camera frames. A background map estimated by the temporal median filter was subtracted from each camera frame before segmentation. The intensity of each sub-region were estimated by summing up the photon counts

in each pixel, after subtracting the median map. An intensity threshold of 1500 photons were applied to the segmented subregions to filter out PSFs with low photon counts.

Supplementary Video 12: Single molecule blinking frames when DL-AO is compensating aberrations induced by refractive index mismatch in Astigmatism-based setup.

Compensations are performed in real time during SMLM experiments when imaging immune-fluorescence-labeled Tom20 specimen. The specimen locates at 146 µm (**Methods**) with refractive indices of sample media and immersion oil being 1.35 and 1.406 respectively measured by Abbe refractometer (334610, Thermo Scientific). The left panel shows the raw blinking frames (after converting the analog-to-digital unit readings in camera frames to the effective photoelectrons, referred as photon #). The displayed frame rate is set to be the same as the acquisition frame rate, which is 50 Hz. The top right panel shows the deformable mirror voltage map w.r.t current detection. The deformable mirror was updated every 100 camera frames. Apparent focal planes were adjusted by moving the sample stage during compensation, since index mismatch induced aberration causes an apparent focal plane shift[4]. A background map estimated by the temporal median filter was subtracted from each camera frame before segmentation. The intensity of each sub-region was estimated by summing up the photon counts in each pixel, after subtracting the median map. An intensity threshold of 1500 photons was applied to the segmented subregions to filter out PSFs with low photon counts.

Supplementary Table 1. Imaging parameters for experimental data

Datasets	Number of frames acquired	Apparent focal plane depth (μm)	Mean $\sqrt{CRLB_x}$ (nm)	Mean $\sqrt{CRLB_y}$ (nm)	Mean $\sqrt{CRLB_z}$ (nm)	Mean photon counts	Mean background counts	Number of localizations
Fig. 3B (Tom20)	100,000	134	9.4	9.1	50.0	2,946	68	1,090,416
Fig. 3E (Tom20)	116,000	134	10.2	9.3	48.7	2,893	67	1,478,280
Fig. 4B (Tom20)	116,000	112	10.7	9.2	48.8	2,641	64	1,729,048
Fig. 5A (amyloid β)	220,000	85	13.4	12.6	56.8	2,920	130	1,155,993
Fig. 5D (amyloid β)	66,000	51	10.7	10.7	51.0	3,091	88	396,333
Fig. 5E (amyloid β)	54,000	67	11.5	11.0	51.1	3,645	142	170,683
Fig. 6A (Thy1-ChR2-EYFP)	146,000	134	13.5	13.4	56.9	3,848	222	958,608
Fig. 6B (Thy1-ChR2-EYFP)	138,000	67	11.3	11.2	51.6	2,972	93	2,407,206
Fig. 6D (Thy1-ChR2-EYFP)	236,000	67	11.1	10.9	51.8	2,708	86	2,648,649
Extended Data Fig. 8 (Tom20)	70,000	92	9.7	8.8	49.6	3,677	95	805,107
Extended Data Fig. 9 (Tom20)	152,000	35	7.8	8.0	39.8	3,355	49	2,367,528
Supp. Fig. 6D (amyloid β)	168394	80	15.7	14.7	58.3	3,159	192	78,000
Supp. Fig. 6E (amyloid β)	175432	95	14.0	14.1	58.7	3,348	180	90,000
Supp. Fig. 6F (amyloid β)	243761	123	12.8	12.8	58.0	2,345	89	122,000

Supplementary Table 2. Detailed sizes in each layer of the network architecture

Building blocks (index)	Components in each building block	Convolutional Kernel size	Stride size	Output size
Conv (1)	Convolutional layer[5,6]<br>→ Batch normalization<br>→ PReLU**	2 × 7 × 7 (biplane)<br>Or 1 × 7 × 7 (1 plane)	1	64 × 32 × 32
Conv (2)	Same as above	64 × 5 × 5	1	128 × 32 × 32
Res* (1-3)	Convolutional layer<br>→ Batch normalization<br>→ Convolutional layer<br>→ Batch normalization<br>→ Convolutional layer<br>→ sum with output of "shortcut" connection[+]<br>→ PReLU	$\begin{bmatrix} 128 \times 3 \times 3 \\ 32 \times 3 \times 3 \\ 64 \times 3 \times 3 \end{bmatrix}$	$\begin{bmatrix} 1 \\ 1 \\ 1 \end{bmatrix}$	$\begin{bmatrix} 32 \times 32 \times 32 \\ 64 \times 32 \times 32 \\ 128 \times 32 \times 32 \end{bmatrix}$
Res (4)	Convolutional layer<br>→ Batch normalization<br>→ Convolutional layer<br>→ Batch normalization<br>→ Convolutional layer<br>→ sum with output of "shortcut" connection[++]<br>→ PReLU	$\begin{bmatrix} 128 \times 3 \times 3 \\ 64 \times 3 \times 3 \\ 128 \times 3 \times 3 \end{bmatrix}$	$\begin{bmatrix} 1 \\ 4 \\ 1 \end{bmatrix}$	$\begin{bmatrix} 64 \times 32 \times 32 \\ 128 \times 8 \times 8 \\ 256 \times 8 \times 8 \end{bmatrix}$
Res (5-7)	Convolutional layer<br>→ Batch normalization<br>→ Convolutional layer<br>→ Batch normalization<br>→ Convolutional layer<br>→ sum with output of "shortcut" connection[+]<br>→ PReLU	$\begin{bmatrix} 256 \times 3 \times 3 \\ 64 \times 3 \times 3 \\ 128 \times 3 \times 3 \end{bmatrix}$	$\begin{bmatrix} 1 \\ 1 \\ 1 \end{bmatrix}$	$\begin{bmatrix} 64 \times 8 \times 8 \\ 128 \times 8 \times 8 \\ 256 \times 8 \times 8 \end{bmatrix}$
Res (8)	Convolutional layer<br>→ Batch normalization<br>→ Convolutional layer<br>→ Batch normalization<br>→ Convolutional layer<br>→ sum with output of "shortcut" connection[+++]<br>→ PReLU	$\begin{bmatrix} 1024 \times 3 \times 3 \\ 256 \times 3 \times 3 \\ 512 \times 3 \times 3 \end{bmatrix}$	$\begin{bmatrix} 1 \\ 8 \\ 1 \end{bmatrix}$	$\begin{bmatrix} 256 \times 8 \times 8 \\ 512 \times 1 \times 1 \\ 1024 \times 1 \times 1 \end{bmatrix}$
Res (9-11)	Convolutional layer<br>→ Batch normalization<br>→ Convolutional layer<br>→ Batch normalization<br>→ Convolutional layer<br>→ sum with output of "shortcut" connection[+]<br>→ PReLU	$\begin{bmatrix} 1024 \times 3 \times 3 \\ 256 \times 3 \times 3 \\ 512 \times 3 \times 3 \end{bmatrix}$	$\begin{bmatrix} 1 \\ 1 \\ 1 \end{bmatrix}$	$\begin{bmatrix} 256 \times 1 \times 1 \\ 512 \times 1 \times 1 \\ 1024 \times 1 \times 1 \end{bmatrix}$
Conv (3)	Convolutional layer	1024 × 1 × 1	1	28

* abbreviate for residual block[7].

** PReLU[8] (Parametric Rectified Linear Unit), with an initial gradient of 0.25.

[+] The output of "shortcut" connection here is the input to the residual block

[++] The output of "shortcut" connection here is result of the input to the residual block going through a 1×1 convolutional layer, with a stride of 4.

[+++] The output of "shortcut" connection here is result of the input to the residual block going through a 1×1 convolutional layer, with a stride of 8.

Supplementary Table 3. Computation Time for compensation with DL-AO

Data	Number of frames per mirror update	Tests	Number of PSFs for estimation	Estimation time (ms)	Segmentation time (ms)
Fig. 2F	20	Test 1	6	55	110
Fig. 2F	20	Test 2	28	76	112
Fig. 2F	20	Test 3	5	52	113
Fig. 2F	20	Test 4	14	63	110
Fig. 2F	20	Test 5	19	66	116
Fig. 2F	20	Test 6	7	66	109
Fig. 2F	20	Test 7	7	61	109
Fig. 2F	20	Test 8	10	56	109
Fig. 2F	20	Test 9	2	64	106
Fig. 2F	20	Test 10	13	50	108
Supp. Fig. 5A	50	Test 1	28	81	218
Supp. Fig. 5A	50	Test 2	17	64	221
Supp. Fig. 5A	50	Test 3	27	70	223
Supp. Fig. 5A	50	Test 4	17	56	220
Supp. Fig. 5A	50	Test 5	18	57	231
Supp. Fig. 5A	50	Test 6	16	60	271
Supp. Fig. 5A	50	Test 7	2	40	265
Supp. Fig. 5A	50	Test 8	28	74	250
Supp. Fig. 5A	50	Test 9	8	57	269
Supp. Fig. 5A	50	Test 10	28	70	299
Fig. 6A	100	Test 1	14	64	773
Fig. 6A	100	Test 2	10	65	797
Fig. 6A	100	Test 3	11	76	770
Fig. 6A	100	Test 4	19	62	765
Fig. 6A	100	Test 5	9	59	759
Fig. 6A	100	Test 6	11	63	751
Fig. 6A	100	Test 7	7	53	776
Fig. 6A	100	Test 8	21	61	754
Fig. 6A	100	Test 9	18	76	766
Fig. 6A	100	Test 10	11	65	803

Note:
1. Computation time was calculated by the difference between two millisecond timers (‘Tick Count (ms)’ function, LabVIEW 2015, National Instruments) before and after performing segmentation/estimation.
2. Estimation time is the total time for: inputting sub-region to neural network (with Python Integration Toolkit for LabVIEW, Enthought Inc.), estimating aberrations with neural network (programed with Python 3.6.5, Anaconda Inc.), and combining network estimations with Kalman filter (programed with LabVIEW 2015, National Instruments).
3. Segmentation time is the total time for biplane registration and segmentation.
4. The aberration estimation was running on a NVIDIA GeForce GTX 1070 graphics card with 8 GB memory. The rest of the computations were performed on an Intel Core i7-5820K processor at 3.30 GHz with 32 GB memory.

Supplementary Table 4. Variation range of parameters in training data generation

Parameters	Range of uniform distributions		
	Network1	Network2	Network3
Mirror Mode 1 and 2⁺	[-1, 1]	[-2, 2]	[-0.5, 0.5]
Mirror Mode 3 and 4	[-1, 1]	[-1, 1]	[-0.5, 0.5]
Mirror Mode 5	[-20, 20]	[-1, 1]	[-0.5, 0.5]
Mirror Mode 6-13	[-1, 1]	[-1, 1]	[-0.5, 0.5]
Mirror Mode 14	[-5, 5]	[-1, 1]	[-0.5, 0.5]
Mirror Mode 15-28	[-1, 1]	[-1, 1]	[-0.5, 0.5]
Photon counts per PSF	[1000, 20000] counts		
Photon counts in detection plane 2 ÷ Photon counts in detection plane 1	[0.9, 1.5]		
Background photon counts per detection plane	[1, 300] counts		
Background photon counts in detection plane 2 ÷ Background photon counts in detection plane 1	[0.9, 1.5]		
PSF position relative to sub-region center (x and y)	[-3, 3] pixels**		
Lateral shift of PSF in detection plane 2 relative to plane 1*	[-1.5, 1.5] pixels		
Molecule's axial position relative to focus*	[-2, 2] μm		
Axial distance between two detection plane	[0.512, 0.64] μm		

⁺ Mirror mode 1-28 can have different shapes and levels in W_{rms} for different optical systems. The coefficient values here are the scaling factors for deformable mirror voltage control. They have arbitrary units. Their actual influences can be estimated by linear combining of measured mirror modes (**Supplementary Note 3**, **Fig. SS4**) then calculating W_{rms} of the composed wavefront. This estimation is accurate only when mirror deforms linearly with input voltages.

* Focus of biplane setup here is defined as the axial position where the PSFs in two detection planes look most similar.

** pixel size is 119 nm.

Supplementary Notes

1. Comparison between DL-AO and metric-based AO

1.1 Comparison between DL-AO and metric-based AO in concept

Wavefront of a single emitter can be measured directly or indirectly if the fluorescent signal is stable and contain enough photon budget[9–12]. Signals from photo-switchable or photo-convertible dyes in SMLM experiments blink stochastically with limited photons, making it difficult to measure wavefront. Still, wavefront can be obtained when building an *in situ* PSF model[3]. However, this process requires accumulating thousands of emission patterns, and removing wavefront variations induced by lateral and axial positions of emitters during the iterative phase-retrieving process. Besides, the retrieved pupil phase wraps when wavefront deviation is larger than a wavelength (e.g. the *in situ* retrieved phase in **Fig. 3F**), which cannot be used to feedback the correction element. Due to these difficulties in inferring aberration during SMLM imaging, current sensorless AO methods compensate aberration by iteratively introducing mirror changes then evaluating these changes with image-quality metrics[13–17]. Though different optimization algorithms[13–17] have been developed to improve the efficiency and robustness of the compensation, the effectiveness relies on the evaluation criteria, i.e. how to quantify the distortion changes. For conventional fluorescent microscopes, the intensity value can reflect distortion level. However, it cannot be adapted to SMLM due to the intrinsic intensity fluctuations in blinking molecules. Observed that high frequency components are less sensitive to intensity fluctuations, current sensorless AO methods describe the distortion level with a weighted sum of spatial frequency components in a SMLM frame, which is usually called an image-sharpness metric. Different weighting methods[13–17] are designed to make the metric values respond correctly to distortion changes. Ideally, the metric values should change quadratically with an increasing

amplitude of each mirror shape (when we scan large enough range of amplitude), and reach a maximum/minimum when the amplitude corresponds to an optimal compensation.

To test the current state-of-art metric design[13,14], we simulated the aberration inferring process. To rule out the sample structure induced variations, we simulated cases where there is only one molecule being detected. When the molecule being detected is right in-focus, we observed that the metric changes quadratically with increasing amplitude, and reaches a maximum value when amplitude is the ground-truth amplitude for optimal compensation (**Extended Data Fig. 1**). However, for the molecule located at 400 nm axially, which is within the commonly captured axial range of SMLM, the ground-truth amplitude corresponds to a minimum metric value, and this minimum value is close to other metric values for amplitude ±2 rad, when scanning the Astigmatism shape. Besides, when scanning the Spherical mode, neither a maximum nor a minimum metric value corresponds to the ground truth amplitude. And similar phenomenon is observed for molecules at 800 nm axial position. These inconsistent, sometimes opposite responses limit the robustness of metric based approaches in SMLM, especially when imaging 3D structures in tissue.

It is difficult to design a feature extractor that summarizes aberration-related information from a SMLM frame, while ignoring irrelevant variations, such as intensity, background, and molecules' positions. Deep neural networks rely on backpropagation[18] to turn the first few layers into an appropriate feature extractor[19], which has been demonstrated to learn the complex relationship from fluorescent beads and single molecule emission patterns to aberration[20–25]. But the downside of automatic feature extraction is that result can become uncontrollable when irrelevant features are extracted for inference. Therefore, covering the sample space in training dataset is of key importance[20]. Through a careful characterization of DL-AO performance, we demonstrated that a trained DL-AO network can give estimation to experimental PSFs achieving a 3D normalized cross correlation (NCC) value of >0.95 when comparing measured PSFs with those generated

from network estimation (**Supplementary Fig. 1**). Feeding back the network estimation to deformable mirror, we demonstrated that DL-AO simultaneously estimates and compensates 28 types of wavefront deformation shapes based on signals from blinking molecules, restores single molecule emission patterns approaching the shapes untouched by sample-induced distortion, and improves the resolution and fidelity of 3D SMLM through thick tissue specimens, with as few as 3-20 mirror changes.

1.2 Comparison between DL-AO and metric-based AO in practice

For SMLM, the shape of PSF is important for achieving optimal resolution[26]. To compare the capability to restore PSF shapes, we tested DL-AO and current state-of-art metric-based AO, REALM[14], on compensating sample induced aberrations based on same area of blinking molecules from immune-fluorescence-labeled Tom20 in COS-7 cells. For sample at coverslip surface, where the aberration is dominated by Coma, both of the methods can restore the PSF shapes. To quantify the restoration, we calculated 3D normalized cross correlation (NCC) between PSFs after AO and the PSFs measured under instrument optimum (**Methods**). We found that the PSFs after DL-AO is closer to the instrument optimum, with a similarity of 0.964 ± 0.06 (mean ± s.t.d, N=6) in NCC, versus a similarity of 0.939 ± 0.031 (mean ± s.t.d, N=6) after metric-based AO (**Fig. 2C**, **2G**). For compensating index mismatch induced aberration at 134 μm from coverslip surface, metric-based AO occasionally improved the PSF shapes but sometimes causes extra distortions, while DL-AO can consistently optimize the PSF shapes, achieving a PSF similarity of 0.933 ± 0.012 (mean ± s.t.d, N=9) (**Fig. 2D**, **2G**, **Supplementary Fig. 4**). This is because metric-based AO aims at improving the image quality, which is described by improving the high frequency content in an image, however, gaining magnitude in high frequency domain doesn't necessarily mean the 3D PSF is optimized.

Since a compensation loop that requires less mirror changes is always preferred to run alongside SMLM imaging, we further compared the number of mirror updates required by DL-AO and metric-based AO using simulated SMLM frame. We performed metric-based AO following the optimal setting of REALM: 12 modes, scanning 9 amplitudes from -1 to 1 radian, 3 rounds of compensation. We compared DL-AO and metric-based AO by checking the residual wavefront after each mirror update, when same initial distortion is generated to both methods. We observed that DL-AO reduces the distortion level to below 0.2 rad in W_{rms} with <5 mirror updates, when estimating from volumetric blinking data (**Supplementary Fig. 5**). But metric-based AO requires >100 updates to achieve the same level, in the case of looking at blinking molecules with no axial distribution. If we test metric-based AO with volumetric data, which is common case for imaging whole cell and tissue, the distortion level barely changed (**Supplementary Fig. 5**). This is an expected result according to the concepts investigation in previous section, the metric behaves inconsistently for molecules at different axial positions (**Extended Data Fig. 1**). For imaging a volumetric sample, molecules from different axial planes (usually from -1~1 μm w.r.t. focus) are detected simultaneously, which would introduce large uncertainties in the corresponding metrics.

To compare metric-based AO and DL-AO under an optimal condition for metric-based AO method, we performed these two methods on compensating aberration using an in-focus fluorescent bead, which is an optimal planar specimen (**Fig. SS1**). We placed 100-nm-crimson bead on top of 200-μm-cut unlabeled brain section and mounted the specimen with refractive index matched media, since current state-of-art metric-based AO requires using refractive index matched specimen with low fluorescent background. We observed the achievable precisions of 24.01 ± 3.45 nm (mean ± s.t.d, N=55) in lateral and 57.26 ± 15.73 nm (mean ± s.t.d, N=55) in axial after compensating tissue-induced aberration with DL-AO, versus the achievable precisions of 25.84 ± 3.71 nm (mean ± s.t.d, N=55) in lateral and 71.72 ± 16.32 nm (mean ± s.t.d, N=55) in axial after compensating

tissue-induced aberration with metric-based AO (**Fig. SS1**). These precisions were calculated based on Fisher information under the condition of 1000 photon counts and 10 background counts. In general, both methods improve the localization precision when compensating aberration with in-focus fluorescent beads. When tissue induced aberration introduces variations in magnitude, e.g. the clipping in pupil magnitude and phase (shown in the last row of **Fig. SS1**), both AO methods designed for compensating phase changes cannot restore PSF to the instrument optimum. One interesting observation is that the wavefront shape in the non-clipping area becomes flatter after compensating with DL-AO.

To further demonstrate that the performance of metric-based AO is sensitive to molecules' positions, we compared metric-based AO and DL-AO with fluorescent beads that are slightly out-of-focus. We observed that the PSFs after metric-based AO deviates from the commonly used Gaussian PSF model, which could introduce localization bias and image artifact in the SMLM reconstruction (**Fig. SS2A**). Assuming that we can get an accurate PSF model for localization, the best achievable precision is 20.92 ± 1.91 nm (mean ± s.t.d, N=55) in lateral and 49.23 ± 18.51 nm (mean ± s.t.d, N=55) in axial after compensating aberration with DL-AO, and the best achievable precision is 27.18 ± 4.82 nm (mean ± s.t.d, N=55) in lateral and 59.30 ± 17.77 nm (mean ± s.t.d, N=55) in axial after compensating aberration with metric-based AO (**Fig. SS2B**). These precisions were calculated based on Fisher information under the condition of 1000 photon counts and 10 background counts.

The lateral and axial resolution are commonly quantified with the lateral and axial normalized intensity profiles of a fluorescent bead in conventional microspore systems. However, SMLM reconstructs a super-resolution image with molecular centers. The resolution of SMLM depends on the accuracy and precision achieved in estimating each emitter' center coordinate. We can see that the intensity profiles from a fluorescent bead cannot reflect the difference in localization precision and difference in the wavefront distortion (**Fig. SS2C-D**)

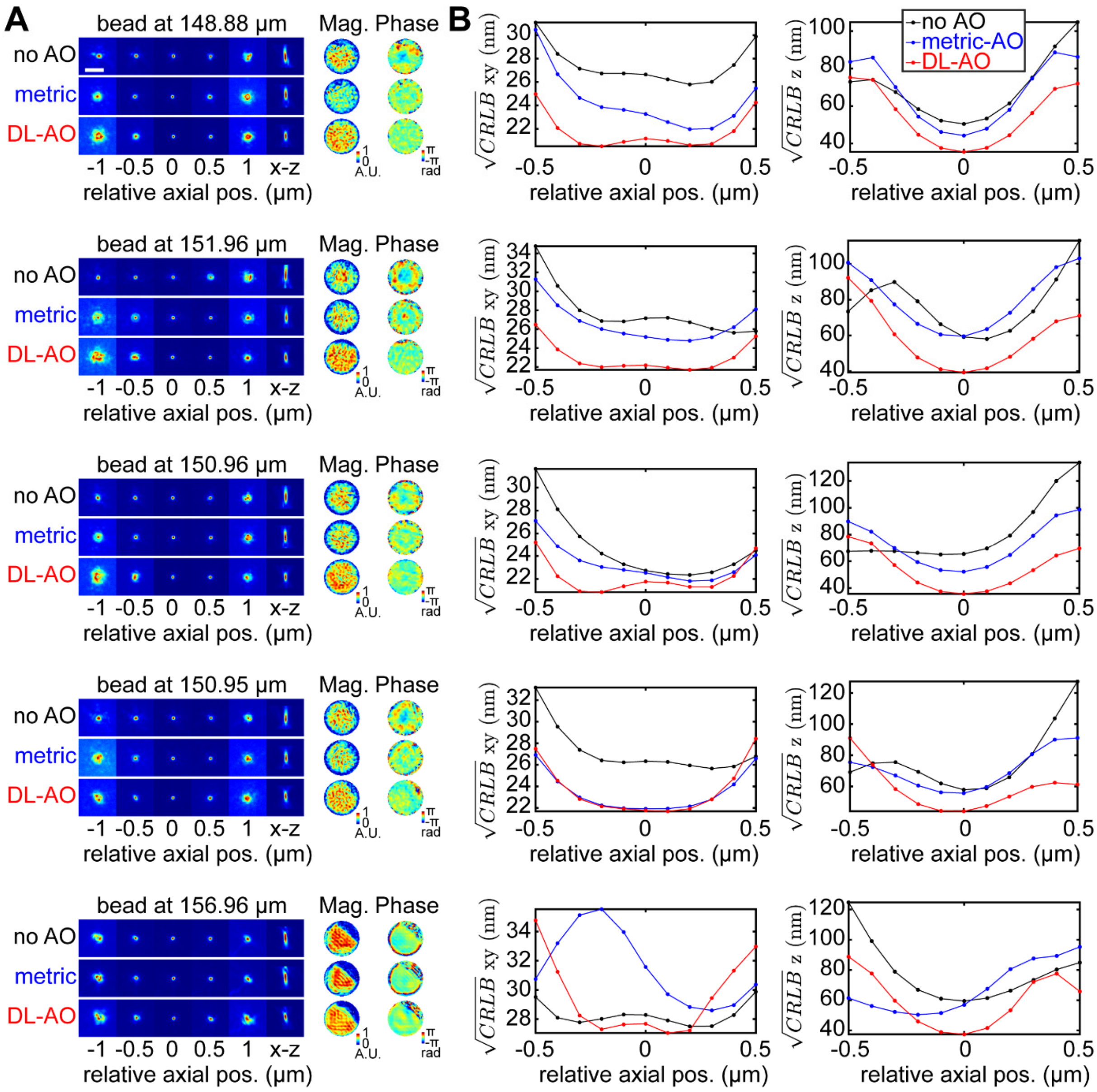

Fig. SS1: Comparison between DL-AO and metric-based AO on compensating aberration using planar fluorescent bead specimen placed on top of 200-μm-cut unlabeled brain section and mounted in refractive index matched media. (**A**) PSFs measured from 100-nm-diameter crimson beads and the corresponding phase retrieved pupil functions before and after AO. Scale bar: 2 μm. (**B**) Theoretically achievable localization precision without and with DL-AO was calculated based on PSF model built from fluorescent beads measurements. The values correspond to PSFs with 1000 total photon counts and 50 background photons per pixel at axial positions of -0.5 μm to 0.5 μm.

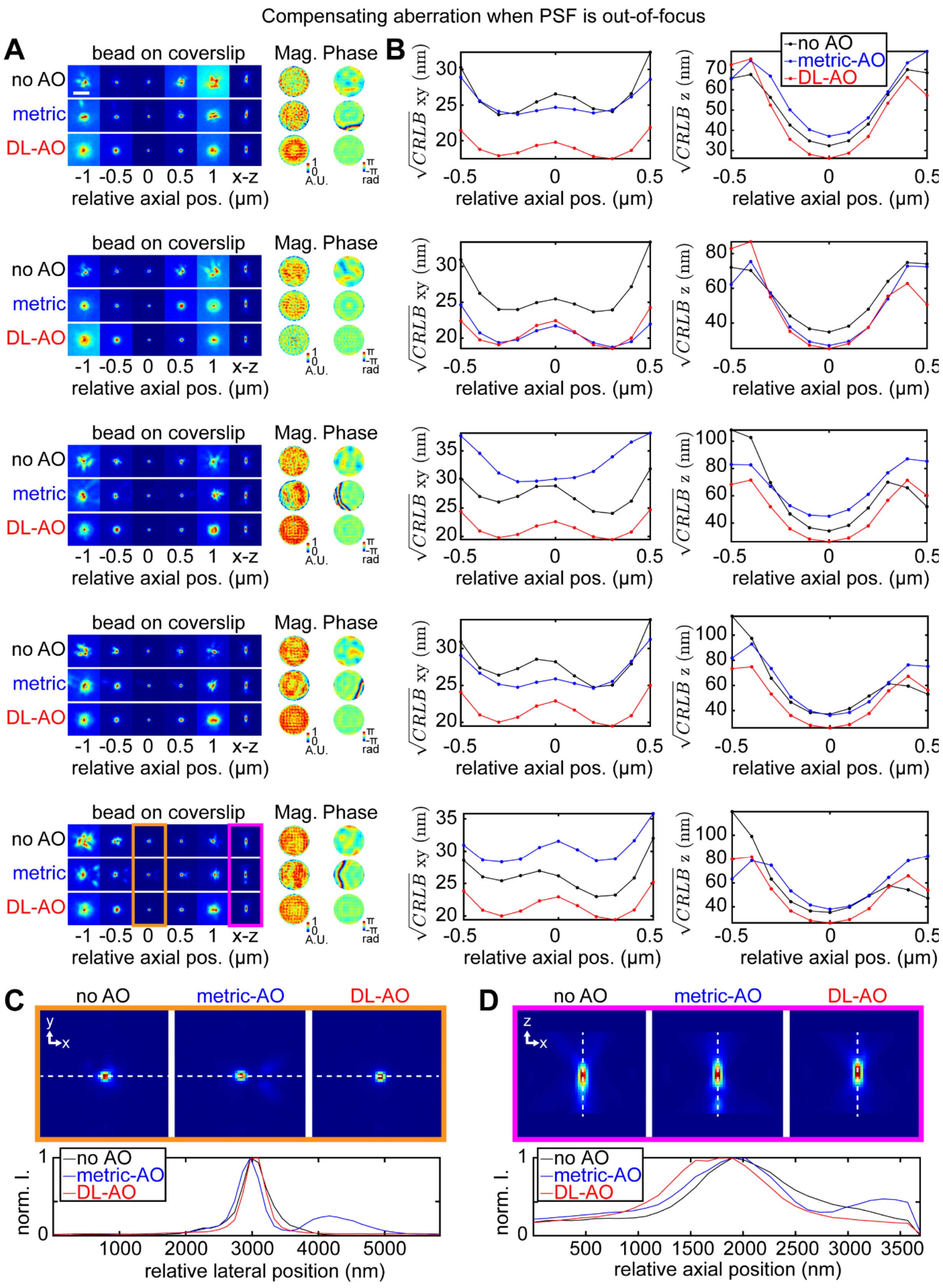
Compensating aberration when PSF is out-of-focus
A
bead on coverslip
Mag. Phase
no AO
metric
DL-AO
-1 -0.5 0 0.5 1 x-z
relative axial pos. (μm)
A.U.
rad
B
√CRLB xy (nm)
√CRLB z (nm)
no AO
metric-AO
DL-AO
relative axial pos. (μm)
C
no AO
metric-AO
DL-AO
norm. I.
relative lateral position (nm)
D
relative axial position (nm)

Fig. SS2: Comparison between DL-AO and metric-based AO on compensating artificially induced aberration using fluorescent bead that are slightly out-of-focus. (**A**) PSFs measured from 100-nm-diameter crimson beads and the corresponding phase retrieved pupil functions without AO, with metric-AO and with DL-AO. Scale bar: 2 μm. The fluorescent beads were placed on coverslip surface. (**B**) Theoretically achievable localization precision without AO, with metric-AO and with DL-AO was calculated based on PSF model built from fluorescent beads measurements. The values correspond to PSFs with 1000 total photon counts and 50 background photons per pixel at axial positions of -0.5 μm to 0.5 μm. (**C**) Comparison on the in-focus PSF shapes and their lateral normalized intensity profiles before and after AO. The PSFs are zoom-in views of regions inside the orange box of A. Line profiles are calculated from the sum projection along the dashed white lines. (**D**) Comparison on the axial PSF shapes and their normalized intensity profiles before and after AO. The PSFs are zoom-in views of regions inside the magenta box of A. Line profiles are calculated from the sum projection along the dashed white lines.

2. Workflow of deep learning driven AO

2.1 General workflow of SMLM imaging with DL-AO

During SMLM imaging, the blinking data were collected at a laser intensity of 2–6 kW/cm^2 and a frame rate of 50 Hz, where the first ~1-100 frames were used for DL-AO. Mirror shape is updated based on DL-AO networks' output every 20-100 frames. In the case where significant background photons were observed (~100 per pixel per frame), a temporal median filter was used to estimate structured background for each pixel, and 100 frames were used to compute this background map. This background map was then subtracted from each camera frame before the frames are segmented into sub-regions for DL-AO processing. After DL-AO correction, 2000 frames were collected per cycle, and 20-120 cycles (50000-236000 frames, **Supplementary Table 1**) were collected per imaging area. For the interleaved SMLM imaging without and with AO, deformable mirror shape was set to switch between DL-AO compensated shape and the shape used for instrument optimum (**Methods**) per imaging cycle (2000 frames). Acquisition of no-AO data was performed first in the interleaved sequence for fair comparison. Upon each switch between no-AO and DL-AO acquisitions, PIFOC objective positioner was moved to compensate apparent focal shift in the case of index mismatch induced aberration[4]. The focal shifts were determined by

an estimated linear relationship between the apparent focus shift and the amplitudes of two radially symmetric mirror deformation modes. The shifts per unit amplitude changes were empirically estimated to be -0.3 μm for mirror mode 5 and -0.2 μm for mirror mode 15 (**Fig. SS6**). Here, a negative movement of PIFOC objective positioner corresponds to shifting the imaging plane closer to the bottom coverslip surface.

2.2 Segmentation process to obtain sub-regions

Before segmentation, the camera offset, with an estimated value of 100 ADU per pixel were removed from each detected camera frame. In the case where significant background photons were observed (∼100 per pixel per frame), a background map estimated by the temporal median filter was subtracted from each camera frame. Then we removed the camera gain by dividing each pixel value with an estimated gain of 2 ADU/e^-. The non-positive pixel values in the each processed camera frame are also set to be 1×10^{-6}. Then each pair of camera frames from two detection planes were sum together, after performing affine transformation to the frames detected on the second plane (‘imwarp’ function with ‘cubic’ interpolation type, MATLAB R2020a, The MathWorks, Inc.) to align the detections from two planes. The transformation matrix was obtained following the previously described method[3]: We first calculated the maximum intensity projection map of 1000-2000 frames containing single-molecule blinking events for each detection plane. Then we calculated the affine matrix based on these projection images in two planes (‘imregtform’ function, MATLAB R2020a, The MathWorks, Inc.). The same transformation matrix was calculated once and used for different specimen to avoid extra time delay in calculating transformation matrix for each frame.

We then segmented out biplane sub-regions, each of 32×32 pixels, using a segmentation algorithm[27]. To locate center coordinates of isolated PSFs in SMLM frames, two uniform filters with different kernel sizes (3 × 3 pixels and 9 × 9 pixels) were applied to each image, where the image is a summation of two frames from two detection planes. The images filtered with larger

kernel size were subtracted from the images filtered with smaller kernel size. Then we applied a maximum filter to the resulting image to locate the pixels containing local maximum intensities. For pixels with local maximum intensities, we considered their positions as candidate sub-region centers if their pixel values are larger than an initial threshold (empirically chosen as 20 photon counts). We then discarded those candidate center coordinates that are closer than 26 pixels to prevent overlapping PSFs in one sub-region. Then we chose center coordinates for cropping sub-regions as the candidate coordinates whose pixel values are larger than a segmentation threshold (empirically chosen as 40-80 photon counts). The center coordinates were used to crop sub-regions out from the first detection plane. For cropping sub-regions in the second detection plane, we transformed coordinates in detection plane 1 to crop the PSF from SMLM frame in detection plane 2.

2.3 Aberration estimation with deep neural network

Pixels in each plane of the bi-plane sub-regions are normalized separately by dividing the maximum pixel value of that plane. Each input sub-region goes through a sequence of template matching processes, which are organized as convolutional layers and residual blocks with PReLU activations and batch normalizations in between, then "fully connects" through 1×1 convolutional layers to an output vector of 28 values — amplitude estimates for wavefront shapes in terms of the native mirror deformation modes[28].

The neural network resembles the architecture as previously developed single molecule network (smNet) for 21 Zernike coefficients' estimations[22], with slight modification to accommodate for the input and output size change. The detailed structure of neural network architecture is shown in **Supplementary Table 2**. Convolutional layers are used throughout the architecture, as studies have shown that deep neural network architectures with the help of convolutional layers are capable of learning relevant features[6,19]. Each convolutional kernel slide through the input images or feature maps, outputting a high value when local features have high similarity to the kernels.

This process is similar to feature extraction process, and the output of each convolutional process is called a feature map. Except for the first two layers and the final layer connecting to the output, all other convolutional layers are packed into residual blocks[7], which add outputs of "shortcut" connections (**Supplementary Table 2**) to the outputs of the stacked layers. The residual blocks were developed to address common issues in training deep architectures, e.g. overfitting, vanishing/exploding gradient[7], and excessive inactive neurons. Due to limited GPU memory and computational resource, it is not practical to update network parameters using the entire training dataset, usually ~6 million images, at the same time. Instead, a batch of 128 images (empirically chosen) is processed together and the gradient for updating network parameter is the average gradient of the batch. In each iteration, the images are processed batch by batch. The normalization step (Batch Normalization[29]) is used to normalize the output distribution of each convolutional layer. Activation functions are added to perform non-linear transform in between linear transformations with convolutional layers. We chose PReLU[8] (Parametric Rectified Linear Unit) as the activation function due to the advantages of no saturation, computational efficient and fast convergence.

2.4 Combining estimation with Kalman filter

Our initial compensation starts when N_0 sub-regions are segmented. The sub-regions are sent to the trained network, which then output N_0 vectors of 28 mirror mode coefficients. Then we calculated the mean and variance for each mirror mode coefficient among the N_0 estimations. The initial mirror update is applied according to the mean of estimation. Then we measure new SMLM frames under the updated mirror shape, and obtain N_1 sub-regions. After calculating the mean and standard deviation among the N_1 estimations, we update deformable mirror by multiplying the current estimation with a Kalman Gain[30] (KG), which is the ratio between variance of the compensation history (till now it is the variance of initial estimation) and the sum of the new variance with history variance. Then the history variance is updated by multiplying

with $(1 - KG)$. Similarly, future compensations will be new estimations damped by Kalman Gain, and we keep updating history variance after each mirror update. Each compensation starts only when $N_i \geq 2$ (for each compensation $i = 0, 1, 2, \cdots$) to make sure there are enough sub-regions for estimating variance. The intuition behind the Kalman filter was to combine noisy measurements that are related, such that the combined prediction become closer to the ground truth in the criterion of mean squared error. Kalman Gain here is a weighting factor between prediction based on previous compensation and a new measurement after previous compensations. When the new measurement has higher variance comparing to the previous compensations, we damp the estimation value based on a variance ratio. The variance of the accumulated compensation will keep decreasing (or keep constant) through combining new measurements. Due to the uncontrollable availability of single molecule emission patterns with high signal-to-background ratio and the evolving PSFs after each correction (**Supplementary Fig. 2**), we use this process to weigh heavily on high precision measurements against the uncertain ones to ensure stable feedbacks from the network. A detailed derivation of Kalman filter implementation for DL-AO is in **Supplementary Note 5**.

2.5 Switching neural networks for estimation

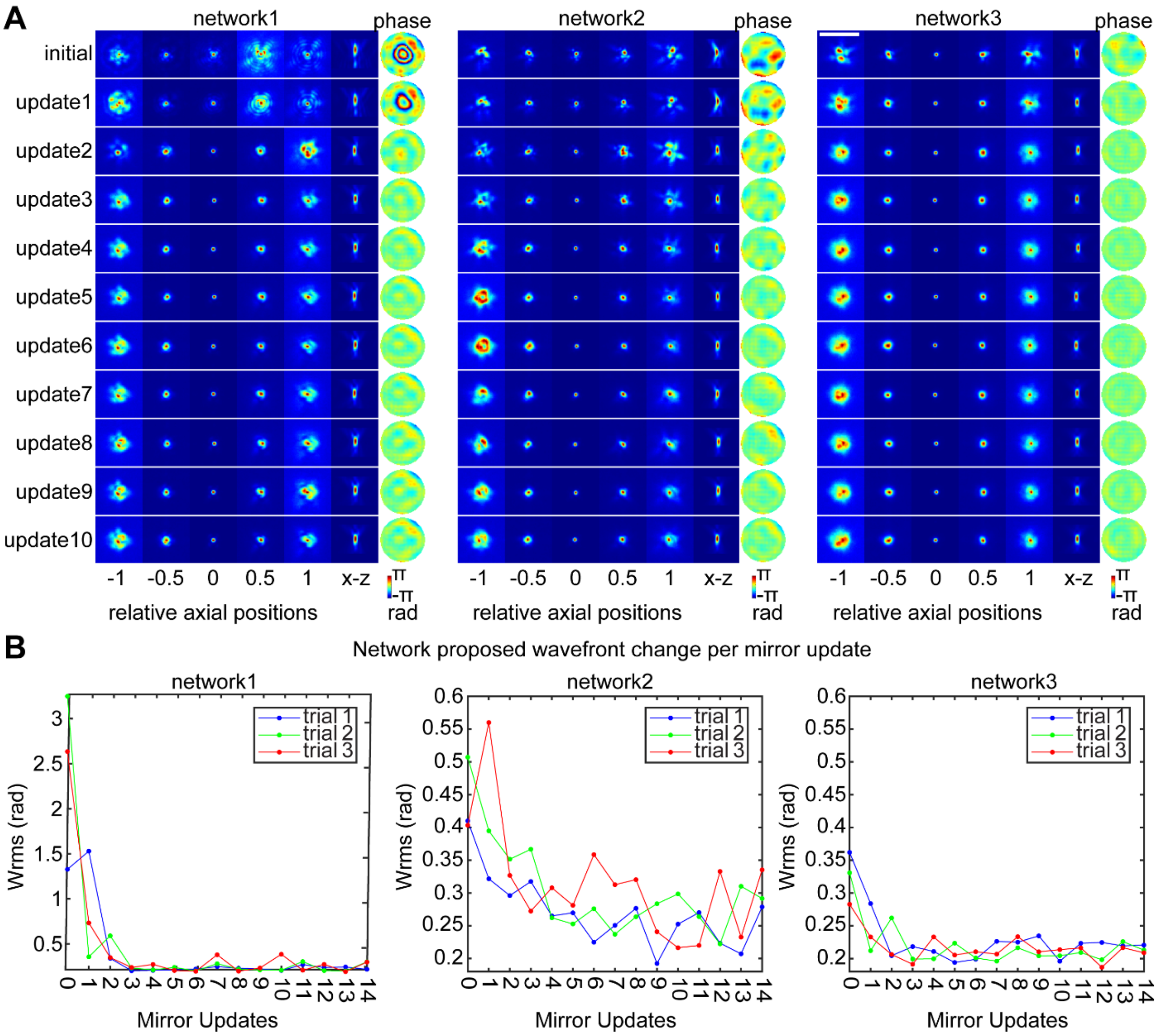

Fig. SS3: Trade-off between compensation range and stability with DL-AO. (**A**) Examples of PSFs before and after each mirror update, when compensating artificially induced aberrations with neural network trained from three different ranges. Compensations are performed based on blinking dyes on immune-fluorescence-labeled Tom20 specimen. PSFs are measured from 100-nm-diameter crimson beads nearby the compensation area post SMLM acquisition. Scale bar: 5 μm. 'phase' stands for pupil phase obtained by phase retrieval on the measured PSFs from beads. Each PSF was normalized to maximum equals to 1. (**B**) Examples of proposed wavefront change per mirror update, when compensating artificially induced aberrations with neural network trained from three different ranges. 'Wrms' stands for root mean square wavefront error (**Methods**). The training ranges for network1-3 are shown in **Supplementary Table 4**.

2.5.1 Reasons of switching networks

Compensating wavefront distortions inferred from PSFs of blinking molecules, we found that the network proposed mirror change fluctuates with non-vanishing uncertainty before/after each mirror update. This uncertainty increases with the network training range, resulting in a trade-off between the compensation range and stability (**Fig. SS3**). To deal with the trade-off between the compensation range and stability, networks trained from three different ranges (**Supplementary Note 4**, **Supplementary Table 4**) are switched for each compensation loop. The detailed training parameters for network 1-3 are shown in **Supplementary Table 4**. For network 2, we observed that independent measurements from DL-AO and phase retrieval[11] using PSFs of fluorescent beads resulted in nearly identical wavefront shapes with a small difference of 0.13 ± 0.02 rad (mean ± s.t.d, N=28) quantified in root mean square wavefront error[31] (W_{rms}, **Methods**, **Extended Data Fig. 3**). Further, comparing the wavefronts estimated by DL-AO network using single molecule blinking data (100 PSFs) to that retrieved by phase retrieval from beads, we observed high similarities of 0.83 ± 0.06 (mean ± s.t.d, N=28, normalized cross correlation), and a small wavefront difference of 0.15 ± 0.03 rad (mean ± s.t.d, N=28) in W_{rms} (**Extended Data Fig. 2**). Using the same dataset as described above, for network1, which included larger variations for Mirror Mode 5 and Mirror Mode 15, the wavefront similarity decrease to 0.84 ± 0.08 and 0.63 ± 0.19 (mean ± s.t.d) in 3D normalized cross correlation (NCC) for beads and cell samples respectively, and wavefront difference increase to 0.17 ± 0.05 and 0.2 ± 0.05 (mean ± s.t.d) in W_{rms} (**Fig. SS4A, C**). And for network3, the wavefront can be estimated with a similarity of 0.83 ± 0.05 and 0.82 ± 0.05 (mean ± s.t.d) in NCC for beads and cells respectively, and wavefront difference of 0.14 ± 0.02 and 0.18 ± 0.03 (mean ± s.t.d) in W_{rms} (**Fig. SS4B, F**). We note that the experimental dataset is used for characterizing the three networks, and the wavefront distortions in these experimental PSFs are outside (~2x larger than) the variation range included in training of network3.

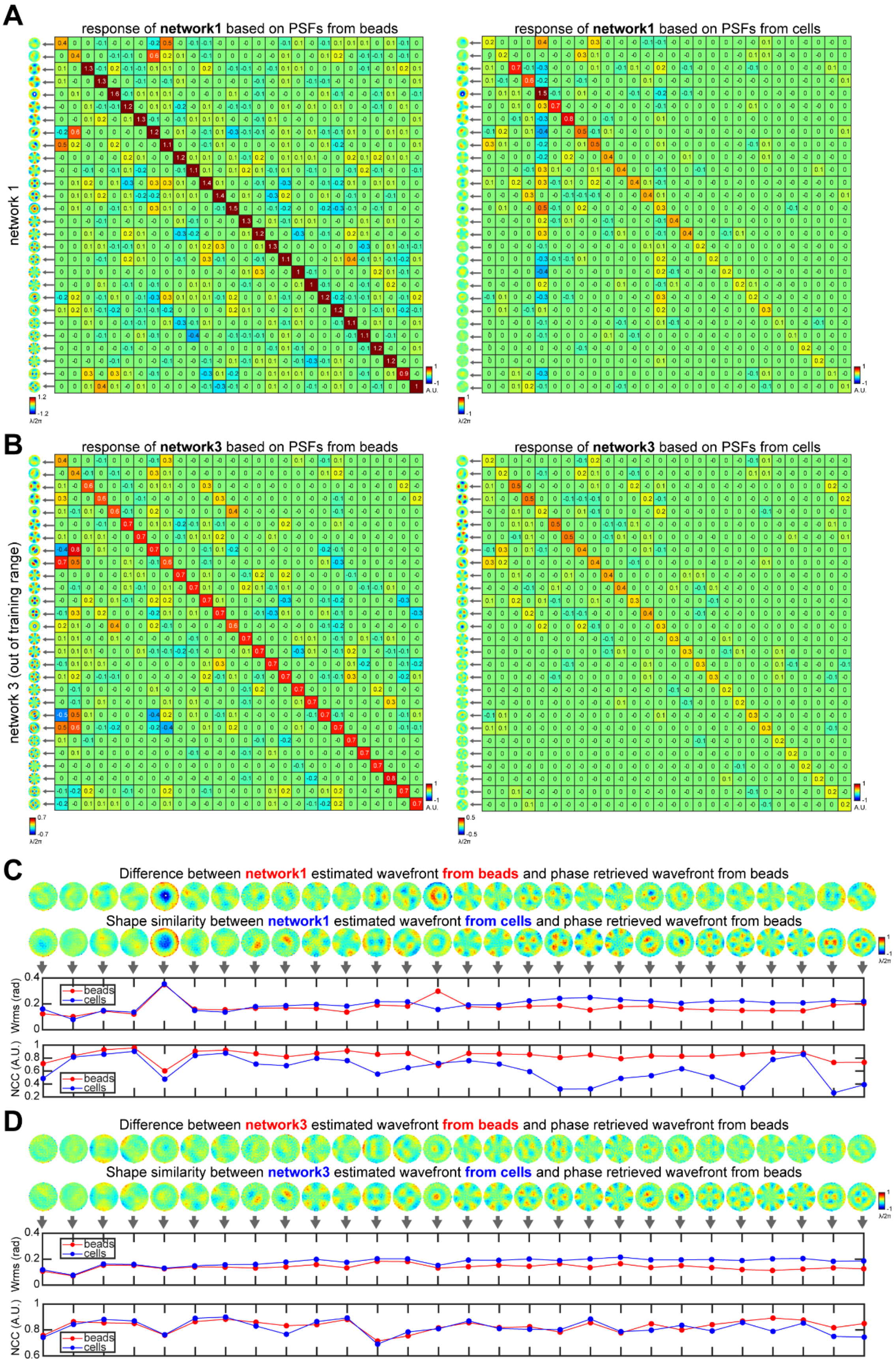
A
response of network1 based on PSFs from beads
response of network1 based on PSFs from cells
network 1
B
response of network3 based on PSFs from beads
response of network3 based on PSFs from cells
network 3 (out of training range)
C
Difference between network1 estimated wavefront from beads and phase retrieved wavefront from beads
Shape similarity between network1 estimated wavefront from cells and phase retrieved wavefront from beads
Wrms (rad)
NCC (A.U.)
beads
cells
D
Difference between network3 estimated wavefront from beads and phase retrieved wavefront from beads
Shape similarity between network3 estimated wavefront from cells and phase retrieved wavefront from beads
Wrms (rad)
NCC (A.U.)
beads
cells

Fig. SS4: Characterizing neural network response to mirror mode changes using experimental PSFs. (**A**, **B**) The responses of networks trained from two different ranges, in complement to the characterization for network2 in **Extended Data Figs. 2, 4**. The three training ranges are shown in **Supplementary Table 4**. Each row of the response matrix shows the network responded mirror coefficients under a unit change of each mirror deformation mode. After linear combining measured mirror modes (**Fig. SS6**) with network responded coefficients, we obtained network estimated wavefront shape w.r.t. individual mirror mode changes. The experimental PSFs were obtained from fluorescent beads (31 PSFs imaged when scanning in axial dimension from -1.5 µm to 1.5 µm with a step size of 0.1 µm) and blinking molecules (100 PSFs) from immune-fluorescence-labeled Tom20 specimen. (**C**, **D**) Pixel-wise differences and shape similarities between network estimated wavefronts and phase retrieved wavefronts, when estimating with network1 and network3 respectively. The top row shows the pixel-wise differences between network estimated wavefront and phase retrieved wavefront using beads. The row below that shows the pixel-wise differences between network estimated wavefront using blinking dyes on Tom20 specimen and phase retrieved wavefront using beads. The plot below that shows the root mean square wavefront error[3] (W_{rms}, **Methods**) of each wavefront difference. The plot on the bottom row shows the similarities between network estimated wavefronts and phase retrieved wavefronts, which are quantified with 2D normalized cross correlation (NCC).

2.5.2 Detailed process in switching networks

The initial compensation starts with estimation from Network1 (**Supplementary Table 4**). By comparing the current estimation variance with two empirically chosen variance-thresholds *th1* and *th2*, we decide whether we will switch to Network2 or Network3 respectively. For example, if current estimation variation is larger than *th1*, the program will use Network1 to continue estimate after the following compensation. With the help of Kalman filter, the history variance will continue decrease (**Supplementary Notes 2.4 and 5.2**) and reach below *th1* or *th2*, the program will then switch to Network2 or Network3 for the following estimation respectively. Whenever we switch to a different network, we reset history variance to be the first estimation variance of the new network. Kalman filter was not applied to Network3, due to its small uncertainty observed and the ignorable PSF shape changes caused by its small uncertainty (**Fig. SS3**). If the current estimation variance with Network3 is larger than *th1* or *th2*, the program will switch back to Network1 or Network2 respectively. We either manually stop the compensation after 10-20 compensations, or stop when

the wavefront change proposed by Network3 has a maximum displacement smaller than 1/20 λ for three consecutive compensations.

3. Considerations in mirror mode generation

3.1 Reasons of using mirror mode

Training neural network for deformable mirror control requires incorporating accurate wavefront deformations in training data generation. To incorporate these, we can measure the wavefront deformations induced by changes of either individual mirror actuator or several actuators together. However, representing wavefront with coefficients of orthogonal basis helps cut down on the number of outputs and network parameters to be optimized in training. Besides, non-orthogonal basis result in non-unique coefficients for representing the same wavefront, which cause vanishing compensation due to the requirement of averaging coefficients from estimations on different sub-regions. Forming this orthogonal basis directly from native mirror deformations further ensured the coefficients' accuracy in representing mirror responses. With this consideration, the conversion from mirror modes to Zernike polynomials—commonly used as the analytical basis to describe aberrations—is dropped to minimize mismatches between mirror responses and Zernike-based wavefront shapes.

3.2 Mirror modes generation process

Mirror mode generation process follows previously described methods[28]. In brief, the steps are: (1) simulating actuators' influence functions, i.e. wavefront deformations introduced by poking individual actuators. The simulation is performed by generating a Gaussian blur at the actuator's location, with Gaussian $\sigma = (-a^2/2\log(0.2))^{1/2}$, where a represents the distance between nearby actuators' centers. (2) multiplying the wavefront deformation with a binary mask representing 2D shape of pupil in the optical system. (3) calculating the cross-talk between

deformations induced by each actuator. (4) generating orthogonal deformation types by linear combining actuators' influence functions. The combination coefficients are found through singular value decomposition of the cross-talk matrix. The final orthogonal deformation types are called mirror modes. The relative actuator voltages for inducing these wavefront deformations in the optical system with deformable mirror are voltage maps of mirror modes.

We observed mismatch between expected wavefront deformations and experimental wavefront deformations, when loading the voltage maps generated in above process to the optical system (**Fig. SS5A**). The expected wavefront deformations come from mirror mode and voltage map generation process as described above. The experimental wavefront deformations are measured through phase retrieval[11] (**Supplementary Note 6.2.1**). Although the expected wavefront deformations are orthogonal with each other, due to the singular value decomposition process, the measured mirror modes are non-orthogonal with each other (**Fig. SS5D-E**). We observed that the experimental wavefront deformations look similar to center areas of the expected wavefront deformations (**Fig. SS5A**), indicating that the expected wavefront deformations were being cut on the boundary in the optical setup. After re-adjusting the relative positions between pupil and mirror actuators (**Fig. SS5C**), we observed experimental mirror modes become more similar to the expected shapes (**Fig. SS5B**). Such adjustment makes experimental mirror modes less coupled with each other (**Figs. SS5F and SS7**). This coupling is verified by performing pixel-wise multiplication between a pair of measured mirror modes, where each mirror mode is normalized by dividing its root mean square. The final relationship between pupil and actuators are adjusted based on physical size of mirror actuators, with slight modification based on the measured influence functions (**Fig. SS5G**). The residual difference between expected and experimental mirror modes (**Fig. SS5B**) is potentially caused by the mismatch between simulated and actual influence function of each actuator (**Fig. SS5G**). Replacing the simulated actuator influence

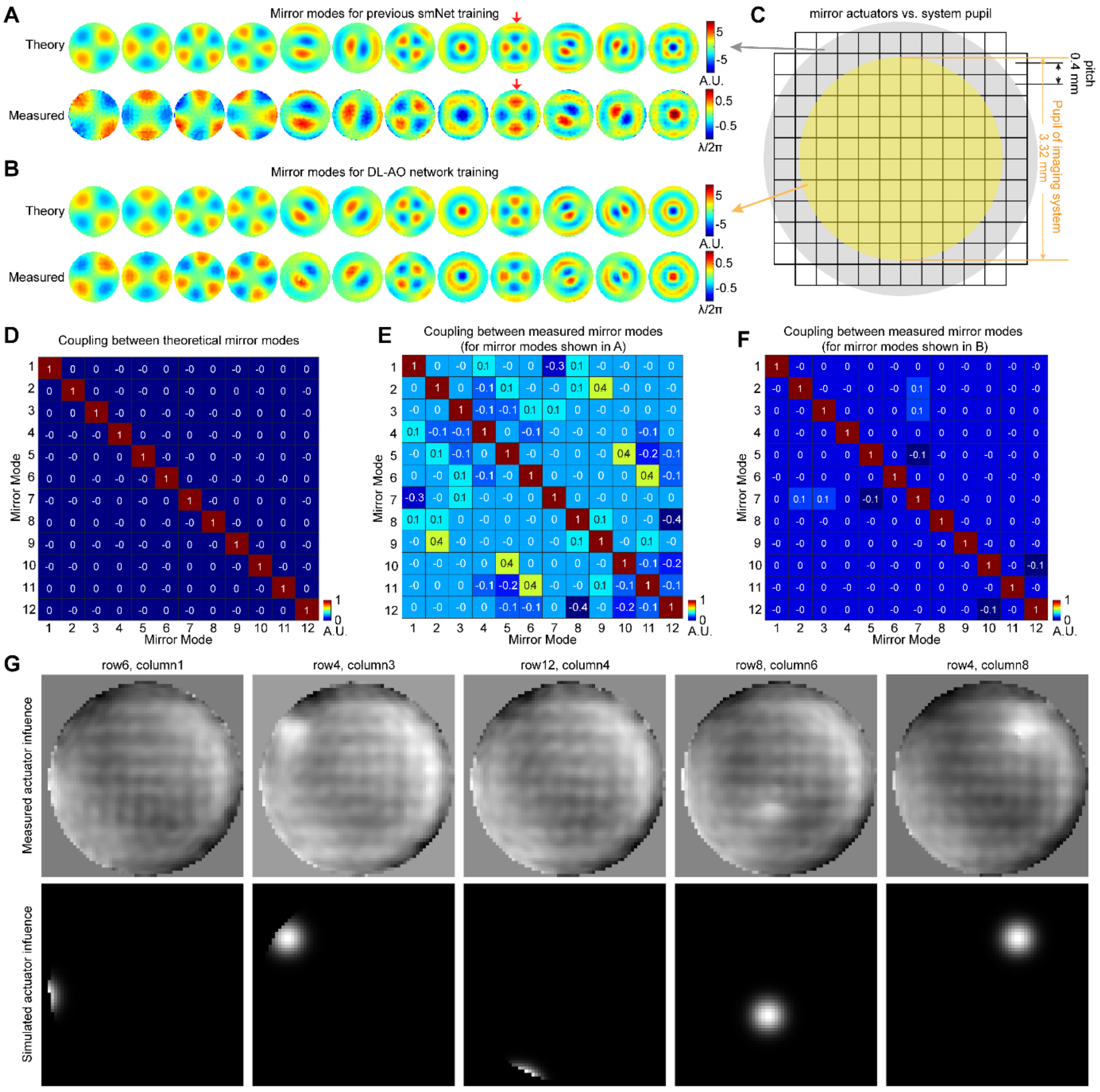

Fig. SS5: Considerations in mirror mode generation. (**A**) Comparison between expected wavefront deformation and the measured wavefront deformation when generating mirror mode voltage maps based on relative actuator vs. pupil position shown in grey area in C. These were the mirror modes converted to Zernike polynomials for testing smNet's responses to Zernike modes in previous work[22]. (**B**) Comparison between expected wavefront deformation and the measured wavefront deformation after updating the relationship of actuator vs. pupil position in C. These are the mirror modes used for DL-AO. (**C**) Relationship of actuator vs. pupil position used for generating mirror mode voltage maps. (**D-F**) Coupling between mirror modes. Each entry is the sum of element-wise multiplication between two mirror mode patterns, which are normalized by dividing root mean square of each pattern. The final matrix is further normalized by dividing the maximum of the entire matrix. (**G**) Comparison between simulated actuator influence functions and those measured through phase retrieval.

function with an experimental actual actuator influence function in the system during mirror mode generation is feasible, however, the generated mirror modes are noisy. Therefore, we proceed with this difference and incorporate this into PSF simulation process by using measured mirror modes.

3.3 Measurements of experimental mirror modes

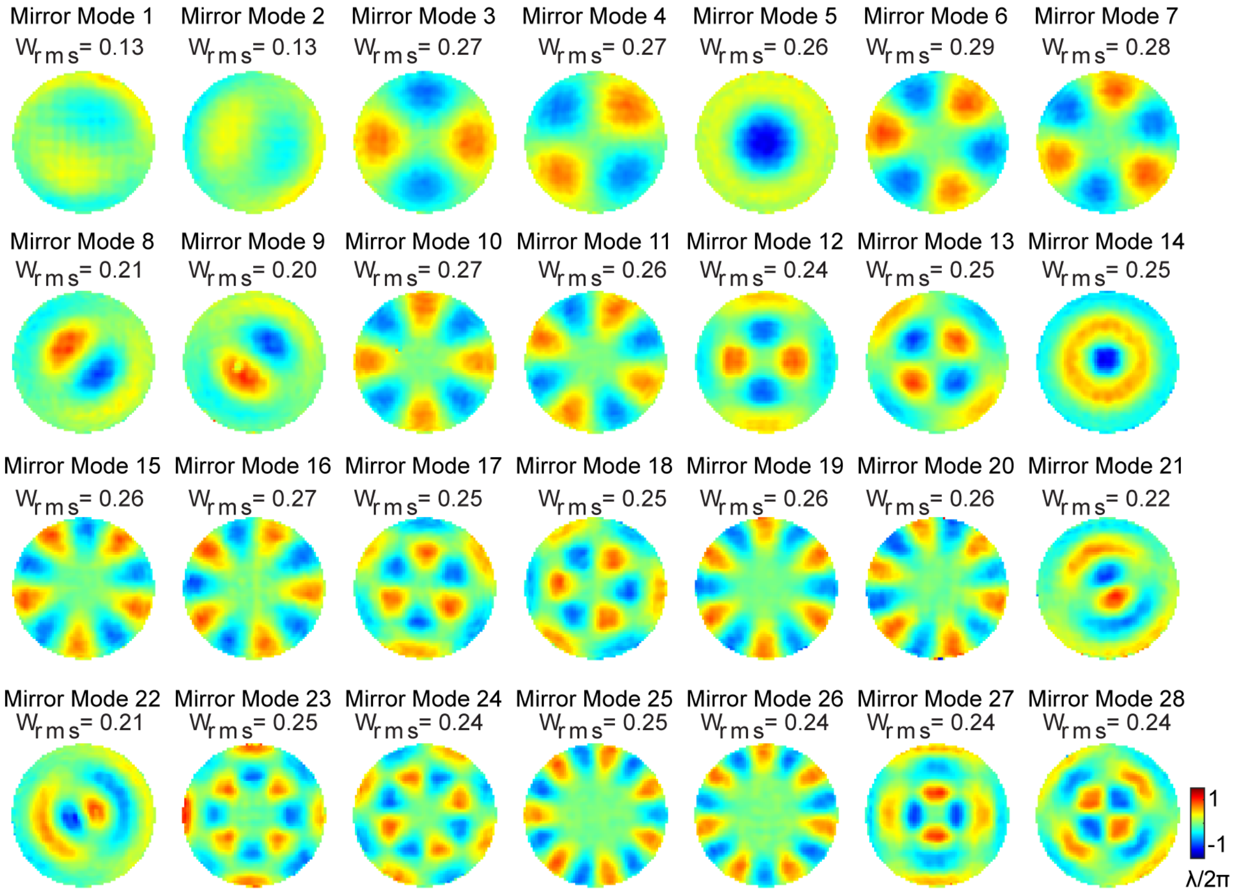

Fig. SS6: Measured mirror modes in optical setup. The mirror modes were measured with phase retrieval on 100-nm-diameter crimson beads (**Methods**). Each mirror deformation mode is generated by introducing a unit change in mirror mode voltage control. The level of distortion introduced by each mirror mode is estimated by calculating the root mean square wavefront error (W_{rms}, **Methods**) of each phase retrieved wavefront. The unit of W_{rms} is λ/2π.

The expected mirror deformations simulated from mirror mode generation process have arbitrary unit, which cannot be directly used for generating PSFs. The experimental deformation in optical

system needs to be measured. The residual differences between theoretical expectations and experimental mirror deformations (**Fig. SS6**) are incorporated into training data generation. The experimental mirror deformation modes[4] were measured using fluorescent bead sample described above. We introduced a positive and a negative (unit amplitude) mirror changes for each of the 28 mirror deformation modes. For each mirror shape setting, we acquired PSFs at z positions from –1.5 μm to 1.5 μm, with a step size of 100 nm, a frame rate of 10 Hz, and 3 frames per z position. Pupil phase was extracted through phase retrieval algorithm[11,28] for each mirror change. To obtain the experimental mirror deformation bases without the influences of instrument or sample induced aberrations, we calculated the differences of the retrieved pupil phases between the positive and negative unit changes of mirror mode and divided them by two. The actual distortion level introduced by each unit amplitude change of mirror mode voltage control is quantified through root mean square wavefront error[31] (**Methods**, **Fig. SS6**).

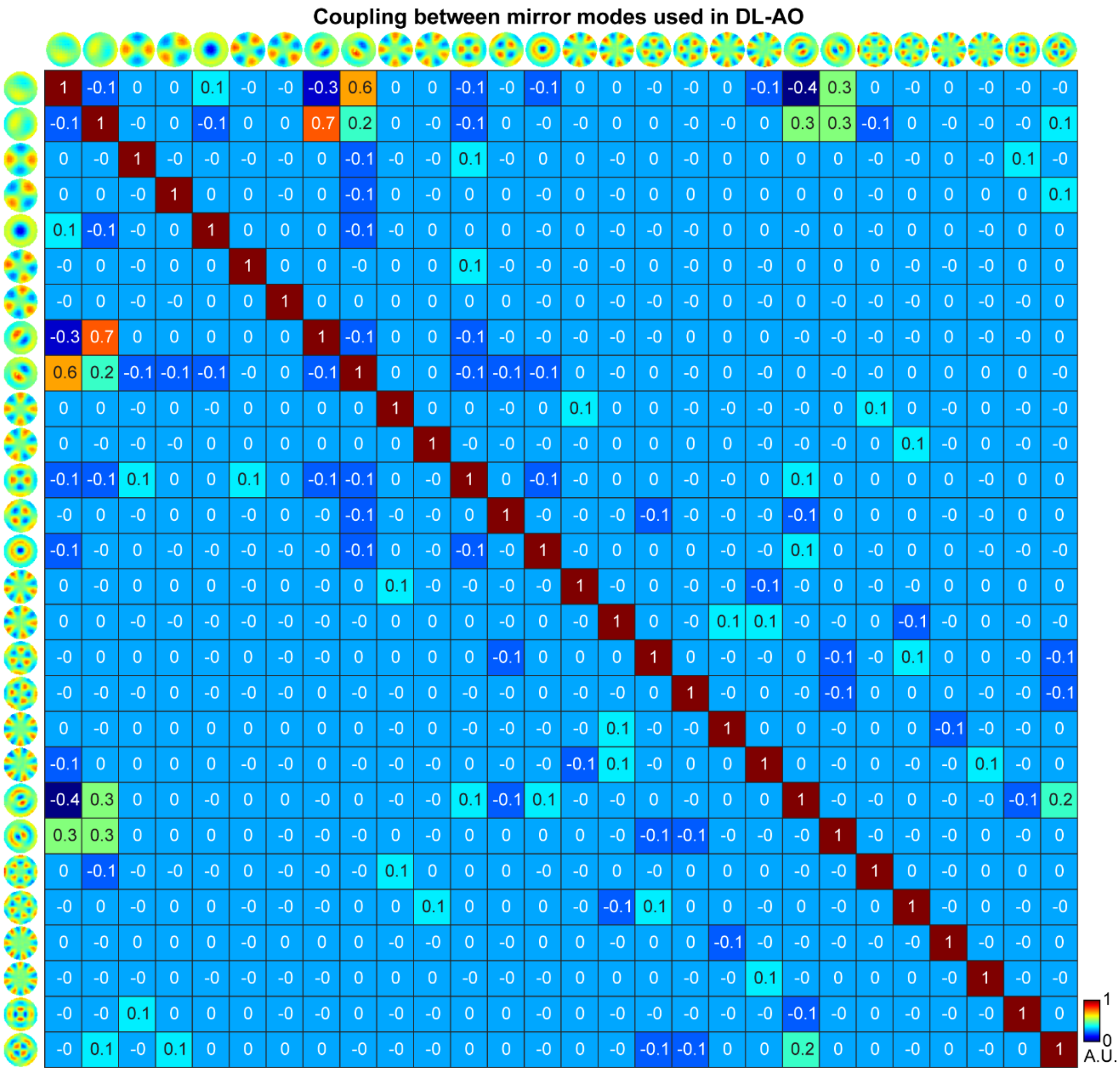

Fig. SS7: Coupling between mirror modes used in DL-AO. Each entry is the sum of element-wise multiplication between two mirror mode patterns, which are divided by root mean square of each pattern to normalize to unit variance. The final matrix is normalized by dividing the maximum of the entire matrix.

4. Training data generation

To build an accurate link between experimentally detected emission patterns and the mirror control with neural networks, it is imperative to train the network with data that match those obtained experimentally. However, experimental training data of single molecules are challenging

to obtain, since the ground-truth wavefronts are usually unknown and the extensive variations of the intensity, background and the lateral and axial locations of single emitters, are impractical to cover experimentally. To this end, we simulate PSFs for training neural network. This allows us to efficiently generate millions of training PSFs based on experimentally measured wavefronts with highly accurate training ground truth (**Supplementary Fig. 1,** 3D normalized cross correlation (NCC) value of >0.95, comparing measured PSFs with those generated from network estimation).

4.1 Measurement of pupil functions under instrument optimum

The static residue of system aberration after optimizing the microscope system is also incorporated as the baseline of the wavefront shapes. We measured the wavefront shape under instrument optimum (**Methods**) with the following steps: (1) collecting a stack of experimental PSFs at z positions from -1.5 to 1.5 μm, with a step size of 100 nm (**Methods**). (2) preprocessing the data to reduce the noise. (3) obtaining the pupil function through an iterative process based on Gerchberg-Saxton algorithm. Following the same process, we obtained two pupil functions $h_1(k_x, k_y)$ and $h_2(k_x, k_y)$ for the two detection planes (**Fig. SS8A**). The relative defocus between two pupil functions were removed during phase retrieval process (**Supplementary Note 6.2.1**). The phase term contains the best achievable wavefront shape when compensating for sample induced aberrations. The common step of decomposing the obtained wavefront φ_o into Zernike polynomials are excluded to avoid residual errors for representing the wavefront after decomposition. We used the Zernike expansion (Wyant ordering) of the pupil function to simulate PSFs at arbitrary positions (x, y, z).

Using biplane setup comes with additional benefits: (1) Simultaneous detection at two axial planes provides improved Fisher information[26] about wave-front distortion than one detection plane[32]. (2) The relative small PSF size compared to that of the Astigmatism setup results in

increased number of sub-regions containing well-isolated emitters and thus the reliability of real-time aberration measurements.

4.2 Simulating PSFs with wavefront distortions

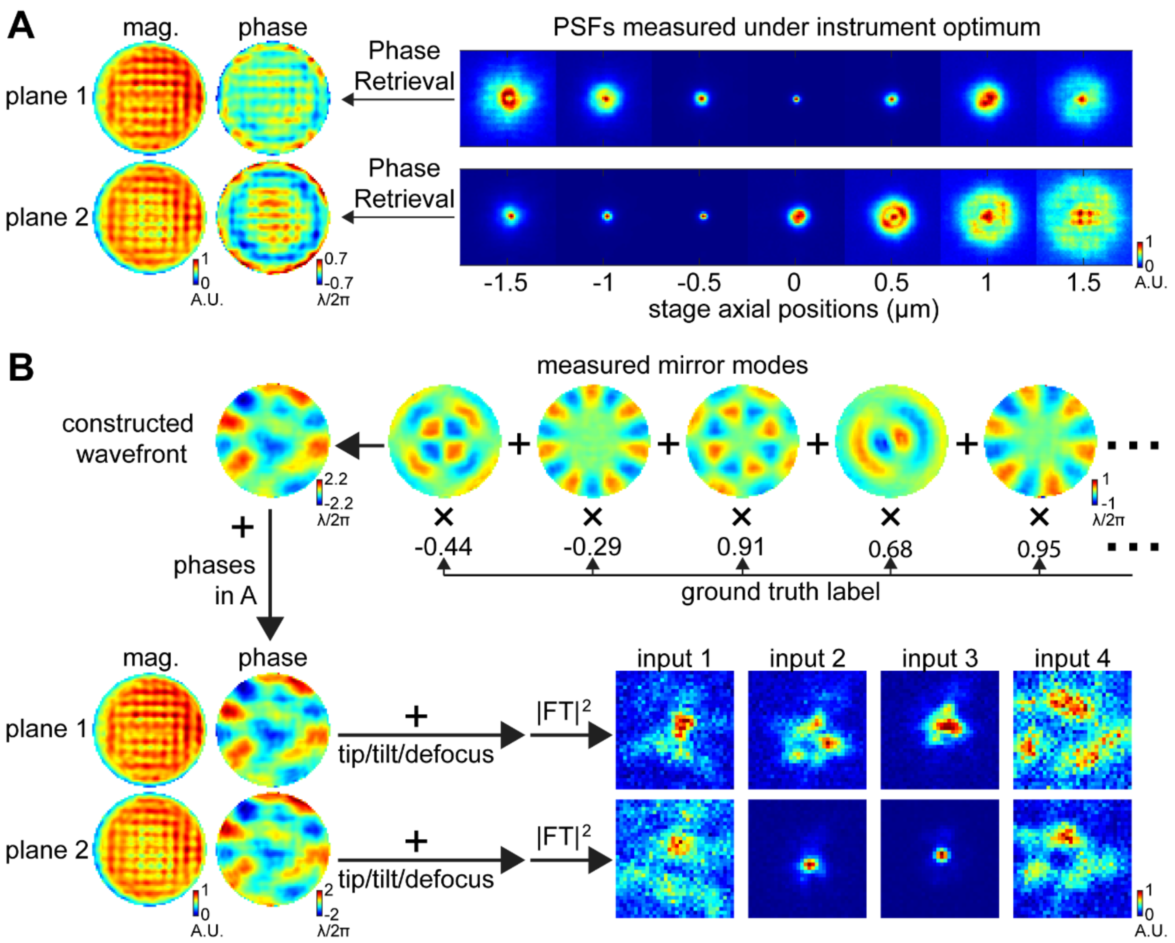

Fig. SS8: Training data generation. (**A**) PSFs and pupil functions measured under instrument optimum for two detection planes. (**B**) Process of simulating aberrated PSFs with constructed wavefront distortion. The tip/tilt/defocus in Zernike Polynomials are added to pupil phase to generate PSFs at different x/y/z positions. The relative focal shift between two detection planes are added by relative defocus difference. FT stands for Fourier Transform of the pupil function. For each wavefront distortion, we can generate various biplane PSFs, the shape variations among which are caused by the variations in molecules' axial positions.

Each PSF was generated as follows: (1) generating wavefront distortion by linear combining measured mirror modes (**Supplementary Note 3.3**) with coefficients $(c_1, c_2 \ldots, c_{28})$. These

coefficients serve as ground truth label for each PSF. (2) generating normalized PSFs for two detection planes, μ_{01} and μ_{02}, at position (x, y, z):

$$\mu_{01}(x, y, z, c_1, c_2 \ \dots, c_{28}) \\ = \left|\mathcal{F}^{-1}\left[h_1(k_x, k_y)\ e^{i(k_x x + k_y y)} e^{ik_z(z-\frac{z_d}{2})} e^{i(\varphi_o + c_1\varphi_{M1} + c_2\varphi_{M2} + \dots + + c_{28}\varphi_{M28})}\right]\right|^2 \quad (1)$$

$$\mu_{02}(x, y, z, c_1, c_2 \ \dots, c_{28}) \\ = \left|\mathcal{F}^{-1}\left[h_2(k_x, k_y) e^{i[k_x(x+\Delta x) + k_y(y+\Delta y)]} e^{ik_z(z+\frac{z_d}{2})} e^{i(\varphi_o + c_1\varphi_{M1} + c_2\varphi_{M2} + \dots + + c_{28}\varphi_{M28})}\right]\right|^2 \quad (2)$$

, where $(\varphi_{M1}, \varphi_{M2} \ \dots, \varphi_{M28})$ represent measured mirror modes (**Supplementary Note 3.3**). The terms $e^{ik_z(z-\frac{z_d}{2})}$ and $e^{ik_z(z+\frac{z_d}{2})}$ describe the defocus phase, where $k_z = ((\frac{2\pi n}{\lambda})^2 - k_x^2 - k_y^2)^{1/2}$ is the axial component of the wave vector ***k*** and z_d represents the axial distance between the two focal planes. (3) multiplying the normalized PSFs with photon count, I, and background count, bg to obtain μ_1 and μ_2.

$$\mu_1(x, y, z, c_1, c_2 \ \dots, c_{28}) = I\mu_{01}(x, y, z, c_1, c_2 \ \dots, c_{28}) + bg \\ \mu_2(x, y, z, c_1, c_2 \ \dots, c_{28}) = r \times I\mu_{02}(x, y, z, c_1, c_2 \ \dots, c_{28}) + r \times bg \quad (3)$$

where *r* represents intensity ratio between two detection planes.

Training a neural network to output the correct mirror mode coefficients while ignoring irrelevant pixel value variations, such as intensity, background, positions shift, is important. This is because we cannot control whether the network learns the correct features relate to the mirror mode coefficients. To avoid irrelevant features being taken into consideration, we generate these aberration-irrelevant parameters from a uniform distribution. The variation range of parameters for generating training dataset are included in **Supplementary Table 4**.

When generating data for training Net1, which contains PSFs resemble index mismatch-induced aberrations, we observed that many sub-regions do not contain recognizable PSFs. This might be caused by the apparent focal shift and the large distortion included. To avoid wasting training

data and training time, we removed the sub-regions that do not contain recognizable PSFs. The selection process is similar to what we have described in **Supplementary Note 2.2** for the segmentation process. In brief, two uniform filters with different kernel sizes (3 × 3 pixels and 6 × 6 pixels) were applied to each simulated sub-region. The sub-regions filtered with larger kernel sizes were subtracted from the sub-regions filtered with smaller kernel sizes. Then we applied a maximum filter to the resulting image, to locate the pixels containing local maximum intensities. For sub-regions containing pixels with local maximum intensities larger than an initial threshold (empirically chosen as 20 photon counts), we accepted these sub-regions as training data.

5. Kalman filter

SMLM experiments require signals from photo-switchable or photo-convertible probes that blink stochastically with limited photons, making it difficult to measure wavefront with wavefront sensor[32], such as a Shack-Hartmann wavefront sensor[33]. In this work, we demonstrated that the trained DL-AO network can estimate 28 types of wavefront deformation shapes based on signals from blinking molecules. Due to the uncontrollable availability of single molecule emission patterns with high signal-to-background ratio and the evolving PSFs after each correction (**Supplementary Fig. 2, Fig. SS9**), we used Kalman filter to weigh heavily on high precision measurements against the uncertain ones to ensure stable feedbacks from the network. Kalman filter is a recursive filter that was introduced by Kalman in 1960[34]. It is a widely used method in many research fields, such as signal processing and autonomous system, and it has been demonstrated in many recent works[35–37] for improved performance when combined with artificial intelligence techniques.

5.1 General concept of Kalman filter

Estimating a parameter based on a single measurement can deviate from the ground truth, as the uncertainty is identical to the standard deviation of measurement noise. Averaging a large

number of repeated measurements reduces the estimation uncertainty, however, these repeats are difficult to carry out in practice. One situation is that the parameter is varying in time, e.g. tracking a car's position with GPS. Another case is that successive measurements can have different inaccuracies and uncertainties over time, e.g. weather change can affect the uncertainties of GPS readings. To prevent our estimation from fluctuating wildly before/after incorporating each new measurement, we can constraint our estimation with the knowledge that successive measurements of the same object are highly related with each other, e.g. car's position readings within 10 minutes cannot be larger than 100 miles. Other prior knowledge can come from either a theoretical model or reading from different sensors, e.g. measurement of a car's speed from odometer.

Kalman filter combines noisy measurements from different sources and the uncertain predictions from theoretical models, such that it tends to give an estimate closer to the ground truth than each single measurement/prediction. More specifically, Kalman filter computes a sequential minimum mean squared error (MMSE) estimator that allows us to estimate the parameter at each time point *n* based on available measurements on and before time *n* as *n* increases[30]. The optimal estimation at time point *n* is recursively computed by a weighted average between the prediction based on all previous information obtained before time point *n* and the new measurements obtained at time point *n*. The weighting factor, named Kalman Gain, is computed based on their uncertainties. Intuitively, this design judges if we should trust more on the new measurements at current time step or on the prediction made based on all previous measurements, according to the uncertainties.

5.2 Kalman filter implementation for deep learning driven AO

Here we describe our application of a scalar Kalman filter in computing an upcoming compensation based on all available wavefront measurements pre-/post- each correction. Before

applying *n^th^* compensation, we describe current ground truth mirror mode coefficient for mirror mode *m* as with $s_m[n]$. We accumulate $N[n]$ sub-regions and send to smNet for wavefront measurement. The measurement for mirror mode m, $x_m[n]$, is noisy due to the uncertainty of neural network estimation and the uncertainty of deformable mirror mechanical movement. To describe this relationship between noisy measurement and ground truth, we can write an equation for each mirror mode *m* as follow, which is called an observation equation:

$$x_m[n] = s_m[n] + w_m[n] \tag{4}$$

, where $s_m[n]$ represent the ground truth value of mirror mode *m* before n^{th} compensation, $w_m[n]$ represent the measurement noise for mirror mode *m*. We assume that $w_m[n]$ is a Gaussian noise with mean $E[w_m[n]\,] = \mu_m[n]$ and variance $E\left[{w_m}^2[n]\right] = \sigma_m^2[n]$ both changing at different *n*. We assume that the noise at different *n* are independent with each other and are uncorrelated with the ground truth $s_m[n]$. We also assume that the measurements of different modes are uncorrelated with each other, so that we can use scalar Kalman filter to compute for each mirror mode separately. The standard deviation $\sigma_m[n]$ can be calculated among smNet measurements based on different sub-regions.

Instead of estimating $s_m[n]$ directly with the measurement $x_m[n]$ (i.e. set them to be equal), which results in an estimation uncertainty same as standard deviation of measurement noise $w_m[n]$, we estimate $s_m[n]$ by combining all available measurements $\{x_m[1], x_m[2], \ldots, x_m[n]\}$ to obtain an estimation $\hat{s}_m[n|n]$ closer to the ground truth $s_m[n]$. The criterion of "being closer to the ground truth" is defined by minimizing the Bayesian MSE:

$$E[(s_m[n] - \hat{s}_m[n|n])^2] \tag{5}$$

where the expectation is taken with respect to $p(x_m[1], x_m[2], \ldots, x_m[n], s_m[n])$. The solution, i.e. the optimal minimum mean squared error estimator (MMSE estimator), is the expectation of the posterior distribution[30]:

$$\hat{s}_m[n|n] = E\big[s_m[n]\big|x_m[1], x_m[2], \dots, x_m[n]\big] \tag{6}$$

To use Kalman filter, the expectation on the right hand side can be simplified as the following equation, assuming the estimator is linear:

$$\hat{s}_m[n|n] = \sum_{k=1}^{n} a_{m,k} x_m[k] \tag{7}$$

, where $a_{m,k}$ are coefficients for linear combining all available measurements.

We then apply our n^{th} compensation by changing the current wavefront shape using deformable mirror. With an ideally behaved deformable mirror, the amount of change is exactly our estimation $\hat{s}_m[n|n]$. Thus, for each mirror mode *m*, the ground truth value is changed to $s_m[n+1]$, which can be described by the following equation, commonly referred as the state equation:

$$s_m[n+1] = s_m[n] - \hat{s}_m[n|n] \tag{8}$$

Since the uncertainty of deformable mirror mechanical movement for each correction is unapproachable, we didn't include it in our state equation.

To restore the coefficients of each mode approaching 0, we need to find an optimal estimate (measured by MMSE), $\hat{s}_m[n|n]$ before each compensation. To find this, we can explicitly solve $a_{m,k}$ in equation (7) for each compensation *n*. But this requires repeated computations with each new measurement arrives. Kalman filter calculates $\hat{s}_m[n|n]$ recursively from $\hat{s}_m[n|n-1]$, the prediction of current mirror mode coefficient based on all previous measurements $\{x_m[1], x_m[2], \dots, x_m[n-1]\}$:

$$\hat{s}_m[n|n] = \hat{s}_m[n|n-1] + K_m[n](x_m[n] - \hat{s}_m[n|n-1]) \tag{9}$$

, $K_m[n]$ is the Kalman Gain, which acts as a weighting factor between new measurements $x_m[n]$ and prediction based on previous measurements, which is defined as[30]:

$$K_m[n] = \frac{M_m[n|n-1]}{M_m[n|n-1] + \sigma_m^2[n]} \tag{10}$$

where the $M_m[n|n-1]$ represents the Bayesian MSE error of the prediction, i,e, estimating $s_m[n]$ based on previous data before $x_m[n]$ is observed, which can be written as following, according to its definition in equation (4):

$$M_m[n|n-1] = E[(s_m[n] - \hat{s}_m[n|n-1])^2] = E[(s_m[n])^2] \quad (11)$$

According to our state equation (8), $s_m[n] = s_m[n-1] - \hat{s}_m[n-1|n-1]$. Thus we can relate the prediction error $M_m[n|n-1]$ with the estimation error before *(n-1)^th^* compensation, $M[n-1|n-1]$, by:

$$M_m[n|n-1] = E[(s_m[n-1] - \hat{s}_m[n-1|n-1])^2] = M[n-1|n-1] \quad (12)$$

The Bayesian MSE error for *n^th^* compensation can be updated recursively based on the error in *(n-1)^th^* compensation by[30]:

$$M_m[n|n] = (1 - K_m[n])M_m[n-1|n-1] \quad (13)$$

Intuitively, this process says that when the uncertainty of the new measurement is much larger than the uncertainty of prediction based on previous measurements, we will trust more on the prediction when estimating for the upcoming compensation. Therefore, as n increases, if we can keep obtaining new measurement which has significantly smaller uncertainty comparing to previous measurements, the accumulated Bayesian MSE error will be reduced. Once the error is reduced to be smaller than certain threshold, we will change the current driving network to another network trained with smaller range.

For the initial condition at $n = 1$, we define $K_m[1] = 1$ and $M_m[1|1]$. According to our definition in equation (8), we assumed that the deformable mirror is behaving ideally, therefore, the prediction based on our previous measurements will be 0, i.e. $\hat{s}_m[n|n-1] = 0$. Thus, equation (9) for combining prediction and new measurement can be simplified as:

$$\hat{s}_m[n|n] = K_m[n]x_m[n] \quad (14)$$

6. PSFs and pupil functions used for characterizing DL-AO performance

6.1 PSF measurements for *in vitro* PSF models

To construct samples with fluorescent beads nearby immune-fluorescence-labeled cells, we diluted 100-nm-diameter crimson beads (custom-designed, Invitrogen) to 1: 1,000,000 in deionized water. Then 500 µL of poly-l-lysine solution (P4707, Sigma-Aldrich) was added to the coverslip with immune-fluorescence-labeled cells, incubated for 20 min and subsequently rinsed with deionized water (pipette gently to avoid washing out cells). We added 1 mL of the diluted bead solution to the coverslip, which was incubated for 20 min at room temperature (RT). Immediately before SMLM imaging, the coverslip without or with specimens attached was placed on a custom-made holder for imaging cells away from or at the bottom coverslip surface respectively. And imaging buffer (10% (wt/vol) glucose in 50 mM Tris, 50 mM NaCl, 10 mM MEA, 50 mM BME, 2 mM COT, 2.5 mM PCA and 50 nM PCD, pH 8.0) was added on top of the bottom coverslip. Then another coverslip with or without specimens was placed on top of the imaging buffer for imaging cells away from or at the bottom coverslip surface respectively. This coverslip sandwich was sealed with two-component silicone dental glue. To control the distance between specimens and bottom coverslip, we used following steps: 200 µL of poly-l-lysine solution was added to cleaned coverslip on bottom, incubated for 20 min and subsequently rinsed with deionized water. Then 20 µL of microsphere suspension (134 µm diameter, 7640A, Thermo Scientific) was spread around the outer ring area of the coverslip, and incubated at RT until the coverslip was dried. Then we placed this coverslip with microspheres at the bottom and added the coverslip with cells on top of it, with the cell-side surface facing down.

After finishing DL-AO compensation on a cell area, we moved manual stage (Manual MicroStage-LT, Mad City Labs Inc.) in lateral dimension to search for area containing fluorescent beads. An

area with one isolated bead, i.e. with no other fluorescent structures within its neighborhood of 60 pixels × 60 pixels, was chosen to measure the 3D PSF stack. The axial position of the bead was adjusted to be approximately in focus in the one detection plane. The PSFs were acquired at a series of z positions from –1.5 µm to 1.5 µm, with a step size of 100 nm, and 3 frames per z position, where z positions are moved by the PIFOC objective positioner (ND72Z2LAQ, Physik Instrumente). The frame rate was chosen from 5-10 Hz and laser power was ~50 W/cm^2. Both frame rate and laser power needs to be adjusted accordingly, such that the PSFs at ±1.5 µm contrast against background and the highest pixel value among the stack doesn't saturate the camera. To acquired 3D PSF stack without DL-AO compensation, we reset deformable mirror the shape as the one obtained for instrument optimum (**Methods**), and following the measurement process as described above. Only one detection plane is used for comparing PSFs without and with DL-AO.

6.2 in vitro PSF models and pupil functions

6.2.1 Phase retrieval process

To obtain the pupil function, which describes how wavefront from a single molecule is affected upon transmission through specimen and imaging system, we performed phase retrieval algorithm on the PSFs measured without and with DL-AO. Multiple phase retrieval methods have been developed for SMLM[11,12,38,39]. In this work, we chose a phase retrieval method[10] based on Gerchberg-Saxon algorithm[10,40,41], which is an iterative process of retrieval the pupil function from a series of PSFs with various amounts of known defocus.

Before performing phase retrieval, the following process was used to preprocess the measured PSFs, which is modified from previous developed method[11]. First, we cropped out an area of 50 × 50 pixels in each acquired camera frame. We then computed an average of the 3 frames acquired at each z position. The camera offset, with an estimated value of 100 ADU per pixel

were removed from each detected camera frame. Then we removed the camera gain by dividing each pixel value with an estimated gain of 2 ADU/e^{-}. The non-positive pixel values in the each processed camera frame are also set to be 1×10^{-4}. The PSF stack is first shifted to its center laterally, with the lateral shift relative to the center of the cropped region estimated by fitting a 2D Gaussian to the most in-focus PSF. Then the background is subtracted from each frame, with background estimated from the minimum of four values, each represent the mean value of the pixels at the four edges of the corresponding cropped image. Following that, a circular mask with diameter of 50 pixels are multiplied to the resulting image to set pixel values outside the mask to be zero. The negative pixel values in the resulting image are also set to be zero. Then the images sizes are restored to 128 × 128 pixels by padding zeros on the boundries. Following that, each image is normalized to sum to 1.

Our parameters used for performing phase retrieval are: numerical aperture of the objective lens *NA* = 1.35, the emission wavelength *λ* = 680 nm, the refractive index of the objective immersion medium *n* = 1.406, the refractive index of the sample medium *n* = 1.35 or 1.406 (measured by Abbe refractometer, 334610, Thermo Scientific) and the effective pixel size of 119 nm on the sample plane. Phase retrieval algorithm was performed on eight PSFs selected from the measured PSF stacks (-1.5 μm, -1.1 μm, -0.7 μm, -0.3 μm, 0.1 μm, 0.5 μm, 0.9 μm, 1.3 μm). For PSF at each axial position, an initial empty pupil was set to start phase retrieval. A defocus phase computed from the expected axial distance to the approximated focus was added to the pupil phase. After performing Fourier transform of the composed pupil, we replaced the magnitude of the resulting image with the square root of the measured PSF. Following an inverse Fourier transform, we updated our pupil function. The defocus phase added before transform was removed from the resulting pupil to estimate the system pupil. Pupil functions computed from all eight PSFs were averaged to complete the first iteration. And the averaged pupil function will be the initial pupil function for the next iteration. The above process was repeated for 25 iterations to

complete the initial phase retrieval process. From the resulting pupil, we estimated the tip, tilt and defocus phase and removed them from the pupil phase to re-center the PSF stack. Subsequently, phase retrieval algorithm was performed again on the measured PSF stack with the updated lateral and axial positions, then a radial modification of the magnitude components of the pupil function was performed on the phase retrieved PSF model to account for the difference between measured PSF and phase retrieved PSF model. The final pupil function is obtained with 10 repeats of re-centering PSF stack, performing phase retrieval to the PSF stack, and modifying the magnitude component of the pupil function.

6.2.2 *in vitro* PSF models and pupil functions

We constructed three different in vitro PSF models and pupil functions for comparing SMLM reconstruction without and with AO. The first one is *in vitro* PSF model and pupil function from bottom beads under instrument optimum, which is used in 'DL-AO+PR' in Figs. 3F, 4B-E, 5B and Supplementary Figs. 8C and 9C. This is obtained by performing phase retrieval on the PSF stacks measured under instrument optimum. The second one is *in vitro* PSF model and pupil function with index mismatched aberration, which is used in 'no AO+PR' in Figs. 3F, 5B and Extended Data Figs. 8C and 9C. This is obtained by performing phase retrieval on the PSF stacks measured next to the compensation area. The third one is *in vitro* PSF model with theoretical index mismatch aberration, which is used in 'no AO+PR' in Fig. 4A and C-E. This is obtained by adding a theoretically derived index mismatch induced aberration phase to the pupil function measured under instrument optimum[11].

6.3 *in situ* PSF models and pupil functions

We constructed in situ PSF models and pupil functions for each compensation area for SMLM reconstruction without and with AO. The models were obtained by performing in situ phase retrieval on the blinking dataset measured for SMLM reconstruction, with INSPR software[3]. For

segmentation process, we set the sub-region size to 32 × 32 pixels, initial intensity threshold to 25, segmentation threshold to 40, distance threshold to 26. And we accumulated at least 3000 PSFs for INSPR model generation. We set similarity threshold to 0.5, and group threshold to 30 during INSPR model generation.

7. DL-AO for Astigmatism-based setup

7.1 Modifications to adapt DL-AO for Astigmatism-based setup

7.1.1 Modifications in Optical Setup

To switch into Astigmatism mode directly from our biplane system (**Methods**), we used signals from one detection plane of the biplane system to perform the tests for Astigmatism-based setup. We introduced a wavefront shape resembling the Astigmatism shape by changing mirror mode 3 (**Fig. SS9**). The experimental mirror deformations (**Fig. SS9**) in optical system were measured following the same procedure described in **Supplementary Note 3.3**. We set the amplitude of Mirror Mode 3 to be +1.5 (A.U.) in the system. This amplitude is chosen empirically. The purpose is to make the amplitude small enough to avoid photons spreading out in PSF, and at the same time make the amplitude large enough to avoid degeneracy problem[3,22], i.e. the situation where different aberrations correspond to the same PSF shape. For certain types of mirror modes, opposite amplitudes result in identical PSFs from opposite axial positions. If left untreated, estimation results might have an opposite sign from the ground-truth aberration amplitude and thus drive the compensation process to distort the PSFs further. Therefore, it is important to avoid the degeneracy problem with pre-engineered PSFs (e.g. Astigmatism in this case), especially when the axial positions are unknown before aberration estimation, a common case for SMLM experiments. A detailed description of this degeneracy problem can be found in a previous work[3].

7.1.2 Modifications in Network Architecture

The only modification to the network architecture is to change the size of the first convolutional kernel from 2×7×7 to 1×7×7 (**Supplementary Table 2**). This modification is necessary, because the biplane subregions contain two input channels (one channel per plane) and the subregions for Astigmatism PSFs contain one channel.

7.1.3 Modifications in Training Data Generation

Each PSF was generated following the process described in **Supplementary Note 4.2**, with only one detection plane being used. In brief, the processes are: (1) generating wavefront distortion by linear combining measured mirror modes (**Fig. SS9**) with coefficients $(c_1, c_2 \ldots, c_{28})$. These coefficients serve as ground truth labels for each PSF. (2) generating normalized PSFs, μ_0 at position (x, y, z):

$$\mu_0(x, y, z, c_1, c_2 \ldots, c_{28}) = \left|\mathcal{F}^{-1}\left[h_1(k_x, k_y)\, e^{i(k_x x + k_y y)} e^{ik_z z} e^{i(\varphi_o + c_1\varphi_{M1} + c_2\varphi_{M2} + \cdots + + c_{28}\varphi_{M28})}\right]\right|^2 \quad (15)$$

, where $(\varphi_{M1}, \varphi_{M2} \ldots, \varphi_{M28})$ represent measured mirror modes (**Fig. SS9**). φ_o represents the Astigmatism wavefront (**Supplementary Note 7.1.1**) on top of the measured instrument optimum wavefront (**Supplementary Note 4.1**). The terms $e^{ik_z z}$ describe the defocus phase, where $k_z = ((\frac{2\pi n}{\lambda})^2 - k_x^2 - k_y^2)^{1/2}$ is the axial component of the wave vector ***k***. (3) multiplying the normalized PSFs with photon count, I, and background count, bg to obtain μ .

$$\mu_1(x, y, z, c_1, c_2 \ldots, c_{28}) = I\mu_0(x, y, z, c_1, c_2 \ldots, c_{28}) + bg \quad (16)$$

The variation range of training parameters were kept the same as the range of the corresponding variables used in biplane setup (**Supplementary Table 4**), which is documented in **Table SS1**.

When generating data for training Net1, we removed the sub-regions that do not contain recognizable PSFs which contains PSFs to avoid wasting training time. The selection process is the same as to what we have described in **Supplementary Note 4.2**.

Table SS1: Variation range of parameters in training data generation for Astigmatism-based setup

Parameters	Range of uniform distributions		
	Network1	Network2	Network3
Mirror Mode 1 and 2^{+}	[-1, 1]	[-2, 2]	[-0.5, 0.5]
Mirror Mode 3 and 4	[-1, 1]	[-1, 1]	[-0.5, 0.5]
Mirror Mode 5	[-20, 20]	[-1, 1]	[-0.5, 0.5]
Mirror Mode 6-13	[-1, 1]	[-1, 1]	[-0.5, 0.5]
Mirror Mode 14	[-5, 5]	[-1, 1]	[-0.5, 0.5]
Mirror Mode 15-28	[-1, 1]	[-1, 1]	[-0.5, 0.5]
Photon counts per PSF	[1000, 20000] counts		
Background photon counts per detection plane	[1, 300] counts		
PSF position relative to sub-region center (x and y)	[-3, 3] pixels*		
Molecule's axial position relative to focus	[-2, 2] μm		

$^{+}$ Mirror mode 1-28 can have different shapes and levels in W_{rms} for different optical systems. The coefficient values here are the scaling factors for deformable mirror voltage control. They have arbitrary units. Their actual influences can be estimated by linear combining of measured mirror modes (**Fig. SS9**) then calculating W_{rms} of the composed wavefront. This estimation is accurate only when mirror deforms linearly with input voltages.

* pixel size is 119 nm.

7.1.4 Modifications in DL-AO workflow

During SMLM imaging, the blinking data were collected as what was described in **Supplementary Note 2.1**. Mirror shape is updated based on DL-AO networks' output every 100 frames. A temporal median filter was used to estimate structured background for each pixel, and 100 frames were used to compute this background map. This background map was then subtracted from each camera frame before the frames are segmented into sub-regions for DL-AO processing. The intensity of each sub-region was estimated by summing up the photon counts in each pixel, after subtracting the median map. An intensity threshold of 1500 photons was applied to the segmented subregions to filter out low intensity segmentations and to improve DL-AO stability for restoring Astigmatism PSFs. When compensating for refractive index mismatch induced aberrations, piezo nano-positioning system (Nano-LP200, Mad City Labs) was moved to compensate apparent focal

shift in the case of index mismatch induced aberration[4]. The focal shifts were determined by an estimated linear relationship between the apparent focus shift and the amplitudes of two radially symmetric mirror deformation modes. The shifts per unit amplitude changes were empirically estimated to be -0.3 μm for mirror mode 5 and -0.2 μm for mirror mode 15 (**Fig. SS6**), which is consistent with experiments in biplane setup.

To segment out subregions containing isolated Astigmatism PSFs, we followed a similar step as what we have described in **Supplementary Note 2.2**. In brief, to locate the center coordinates of isolated PSFs in SMLM frames, two uniform filters with different kernel sizes (3 × 3 pixels and 9 × 9 pixels) were applied to each image. The images filtered with larger kernel sizes were subtracted from the images filtered with smaller kernel sizes. Then we applied a maximum filter to the resulting image to locate the pixels containing local maximum intensities. For pixels with local maximum intensities, we considered their positions as candidate sub-region centers if their pixel values are larger than an initial threshold (empirically chosen as 20 photon counts). We then discarded those candidate center coordinates that are closer than 26 pixels to prevent overlapping PSFs in one sub-region. Then we chose center coordinates for cropping sub-regions as the candidate coordinates whose pixel values are larger than a segmentation threshold (empirically chosen as 40-80 photon counts). The center coordinates were used to crop sub-regions out.

Before inputting sub-regions to the DL-AO network, pixels in each segmented sub-region are normalized separately by dividing the maximum pixel value of that sub-region. The initial compensation starts with estimation from Network1 (**Table SS1**). By comparing the current estimation variance with two empirically chosen variance-thresholds *th1* and *th2*, we decide whether we will switch to Network2 or Network3 respectively. We manually stop the compensation after ~20 compensations in the current investigations.

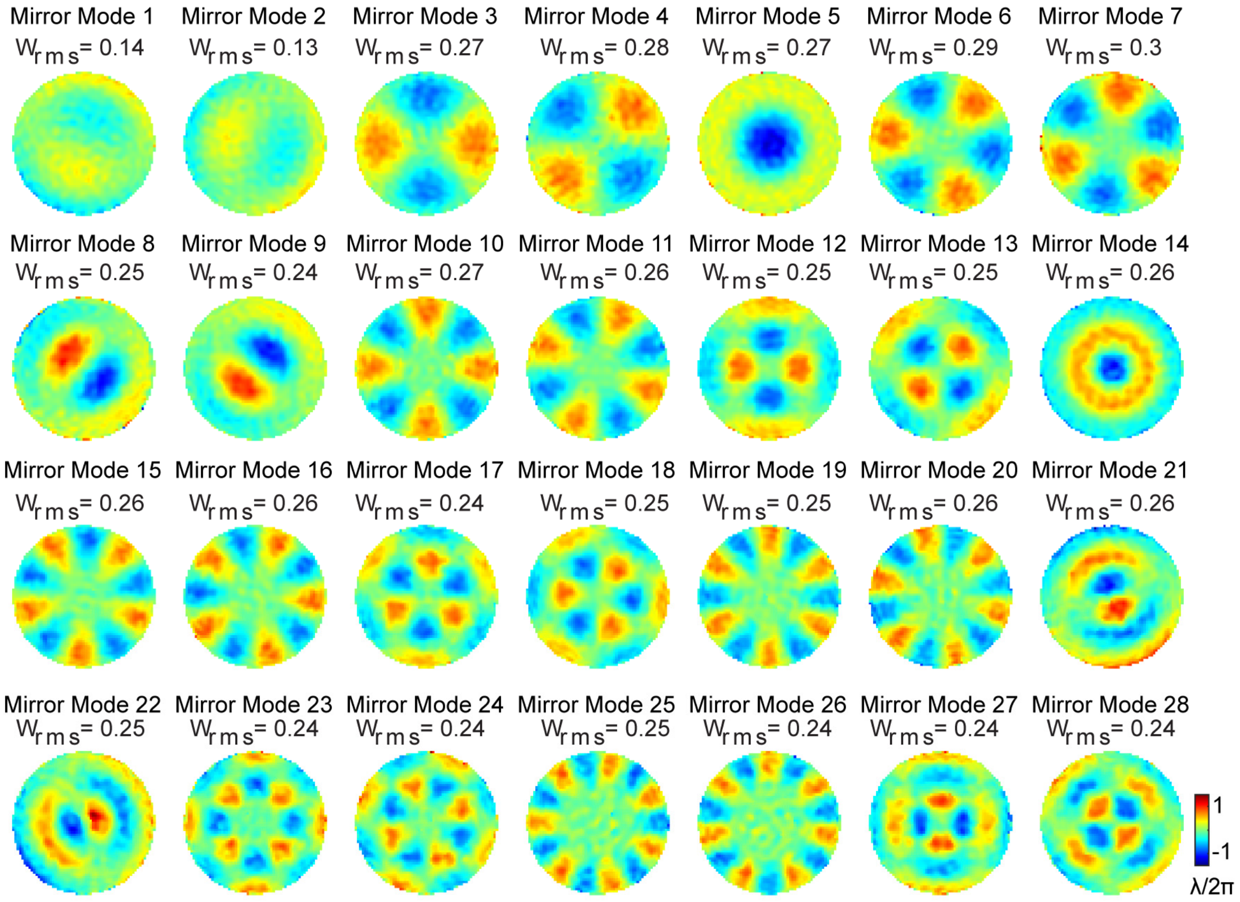

Fig. SS9: Measured mirror modes used for Astigmatism-based setup. The mirror modes were measured with phase retrieval on 100-nm-diameter crimson beads (**Methods**). Each mirror deformation mode is generated by introducing a unit change in mirror mode voltage control. The level of distortion introduced by each mirror mode is estimated by calculating the root mean square wavefront error (W_{rms}, **Methods**) of each phase retrieved wavefront. The unit of W_{rms} is λ/2π. Mirror Mode looks slightly different with the corresponding Mirror Modes in **Fig. SS6** and due to deformable mirror repositioning.

7.2 Response accuracy of DL-AO network for Astigmatism-based setup

To check whether our network was trained to capture the experimental features, we compared measured PSFs with PSFs simulated from network estimations based on a single measurement of an isolated molecule in Astigmatism setup (**Fig. SS10**). To obtain experimental PSFs, we measured PSFs from 100-nm-diameter crimson beads when scanning the piezo nano-positioning system at different axial positions. A single sub-region was sent to the trained neural network,

which outputs a vector of mirror mode coefficients. The measured mirror modes (**Fig. SS9**) were linearly combined with mirror mode coefficients output from network, which gives us wavefronts estimated by the neural network. The wavefront was then used to simulate PSFs at different axial positions to check the similarity between measured PSFs and PSFs simulated from network estimations. We observed that the PSF simulated based on network estimation (from a single subregion) approaches the PSF obtained experimentally (**Fig. SS10**).

As what we have tested for biplane setup, we first characterized the response accuracy of DL-AO network using controlled wavefront distortions generated by the deformable mirror. These wavefront distortions resulted in aberrated emission patterns, which were then collected and sent to DL-AO network (**Supplementary Note 7.1.4**). We induced mirror changes on top of the Astigmatism shape generated by Mirror Mode 3. By comparing the induced deformation amplitudes with those estimated by DL-AO, we observed that DL-AO network responded toward individual mirror deformations mostly in a one-to-one manner. And this behavior was consistently observed with both beads samples and blinking single molecules from immune-fluorescence-labeled cell specimens (**Figs. SS11-12**). We observed that independent measurements from DL-AO and phase retrieval[10,32] using PSFs of fluorescent beads resulted in nearly identical wavefront shapes with a small difference of 0.11 ± 0.02 rad (mean ± s.t.d, N=28) quantified in root mean square wavefront error[31] (W_{rms}, **Methods**, **Fig. SS11A-B**). Further, comparing the wavefronts estimated by DL-AO network using single molecule blinking data to that retrieved by phase retrieval from beads, we observed high similarities of 0.78 ± 0.09 (mean ± s.t.d, N=28, normalized cross correlation), and a wavefront difference of 0.17 ± 0.03 rad (mean ± s.t.d, N=28) in W_{rms} (**Fig. SS12**).

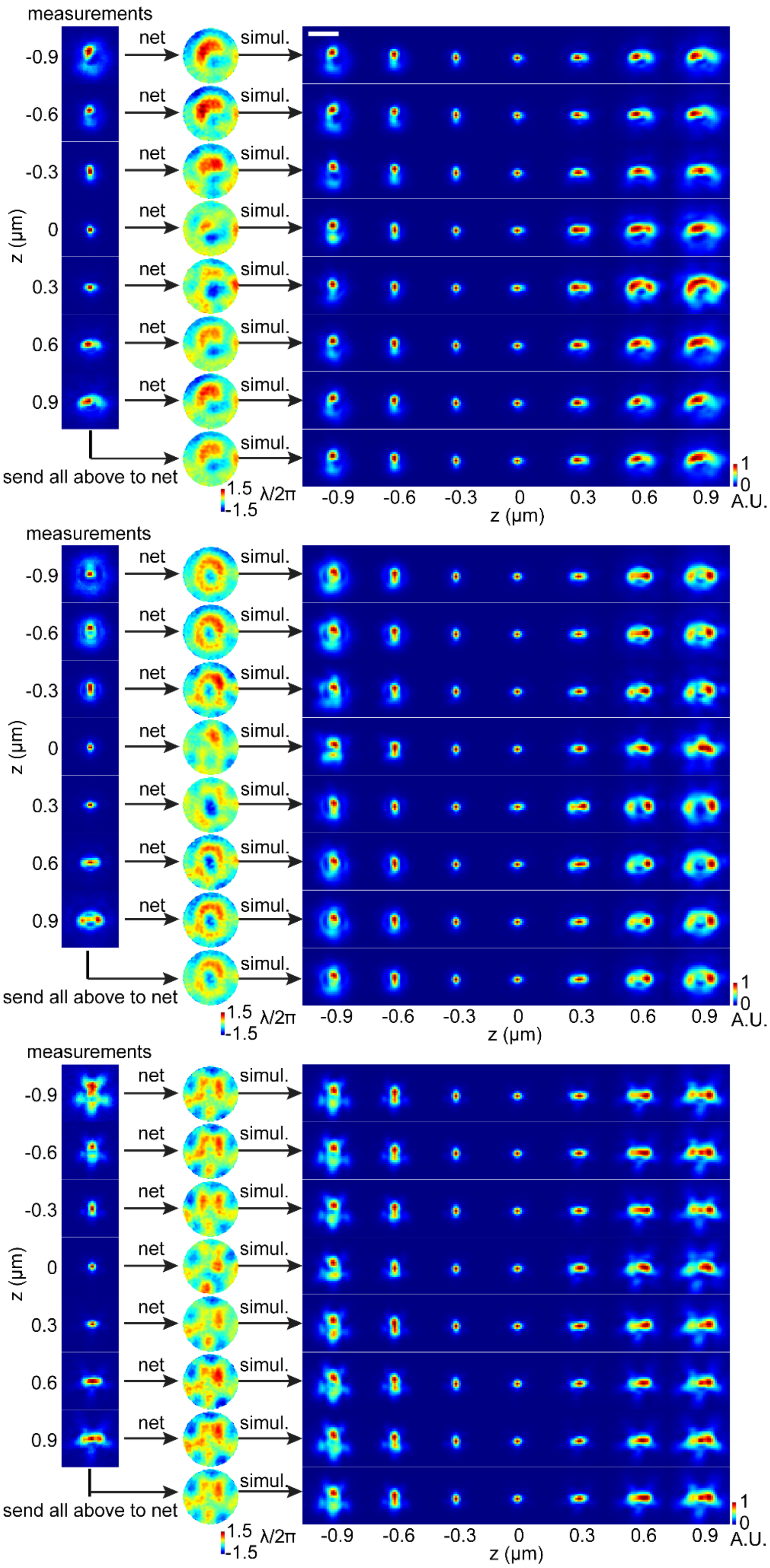
measurements
net
simul.
-0.9
-0.6
-0.3
0
0.3
0.6
0.9
z (µm)
send all above to net
1.5 λ/2π
-1.5
-0.9 -0.6 -0.3 0 0.3 0.6 0.9
z (µm)
1
0
A.U.

Fig. SS10: Comparison between measured PSFs and PSFs simulated from network estimations based on a single measurement of an isolated molecule in Astigmatism setup. The left column shows measured PSFs from 100-nm-diameter crimson beads when scanning Piezo stage at different axial positions. The measured PSFs in biplane sub-regions were sent to neural network, which outputs a vector of mirror mode coefficients for each sub-region. The measured mirror modes (**Supplementary Note 3**) were linearly combined with mirror mode coefficients output from network, which result in wavefronts shown in the middle column. The wavefront for network estimations based on all measured PSFs was generated with an averaged value among network outputs w.r.t. PSFs at different axial positions. The wavefront was then used to simulate PSFs at different axial positions to check the similarity between measured PSFs and PSFs simulated from network estimations. PSFs were simulated without background and noise for visualization. Scale bars: 2 µm.

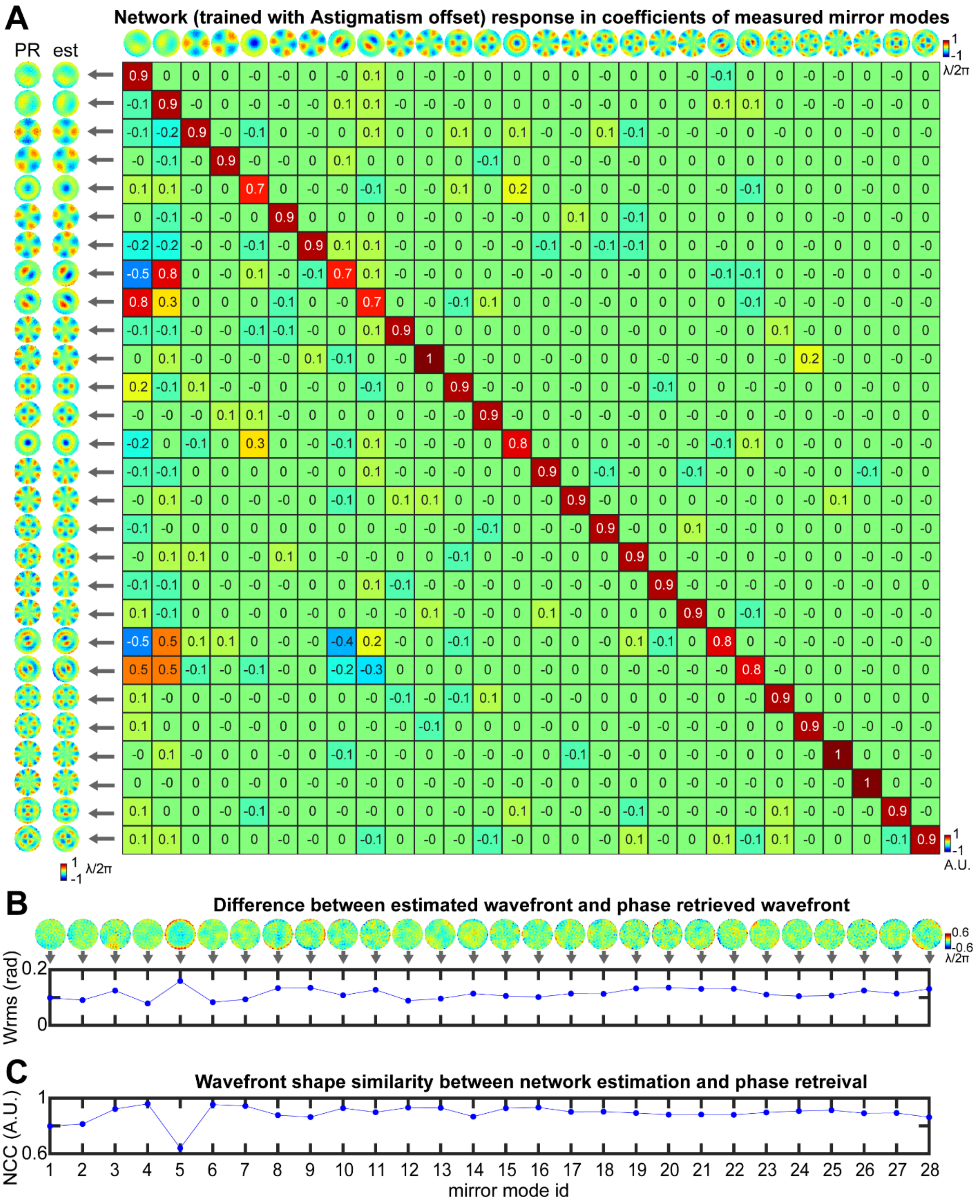
A
Network (trained with Astigmatism offset) response in coefficients of measured mirror modes
PR
est
λ/2π
A.U.
B
Difference between estimated wavefront and phase retrieved wavefront
0.6
-0.6
λ/2π
Wrms (rad)
0.2
0
C
Wavefront shape similarity between network estimation and phase retreival
NCC (A.U.)
1
0.6
1 2 3 4 5 6 7 8 9 10 11 12 13 14 15 16 17 18 19 20 21 22 23 24 25 26 27 28
mirror mode id

Fig. SS11: Characterizing neural network (trained with Astigmatism offset) responses to mirror mode changes using PSFs measured from fluorescent beads. (**A**) Network response to individual mirror mode changes. Each row of the response matrix shows the network responded mirror coefficients under a unit change of each mirror deformation mode. After linear combining measured mirror modes (images below the title) with network responded coefficients, we obtained network estimated wavefront shape w.r.t. individual mirror mode changes (the 2nd column). The 1st column shows phase retrieved wavefronts from beads imaged individual mirror mode changes. The PSFs were measured with 100-nm-diameter crimson beads. PSFs from -1.5 μm to 1.5 μm around the focus, with 0.1 μm step size, were collected for characterizing network responses. (**B**) Difference between network estimated wavefront and phase retrieved wavefront (the first two columns in A). The top row shows the pixel-wise differences between wavefronts obtained from network estimation and that obtained from phase retrieval. The plot below shows the root mean square wavefront error[31] (W_{rms}, **Methods**) of each wavefront difference. (**C**) Similarity between network estimated wavefront and phase retrieved wavefront. The similarity is quantified with 2D normalized cross correlation (NCC).

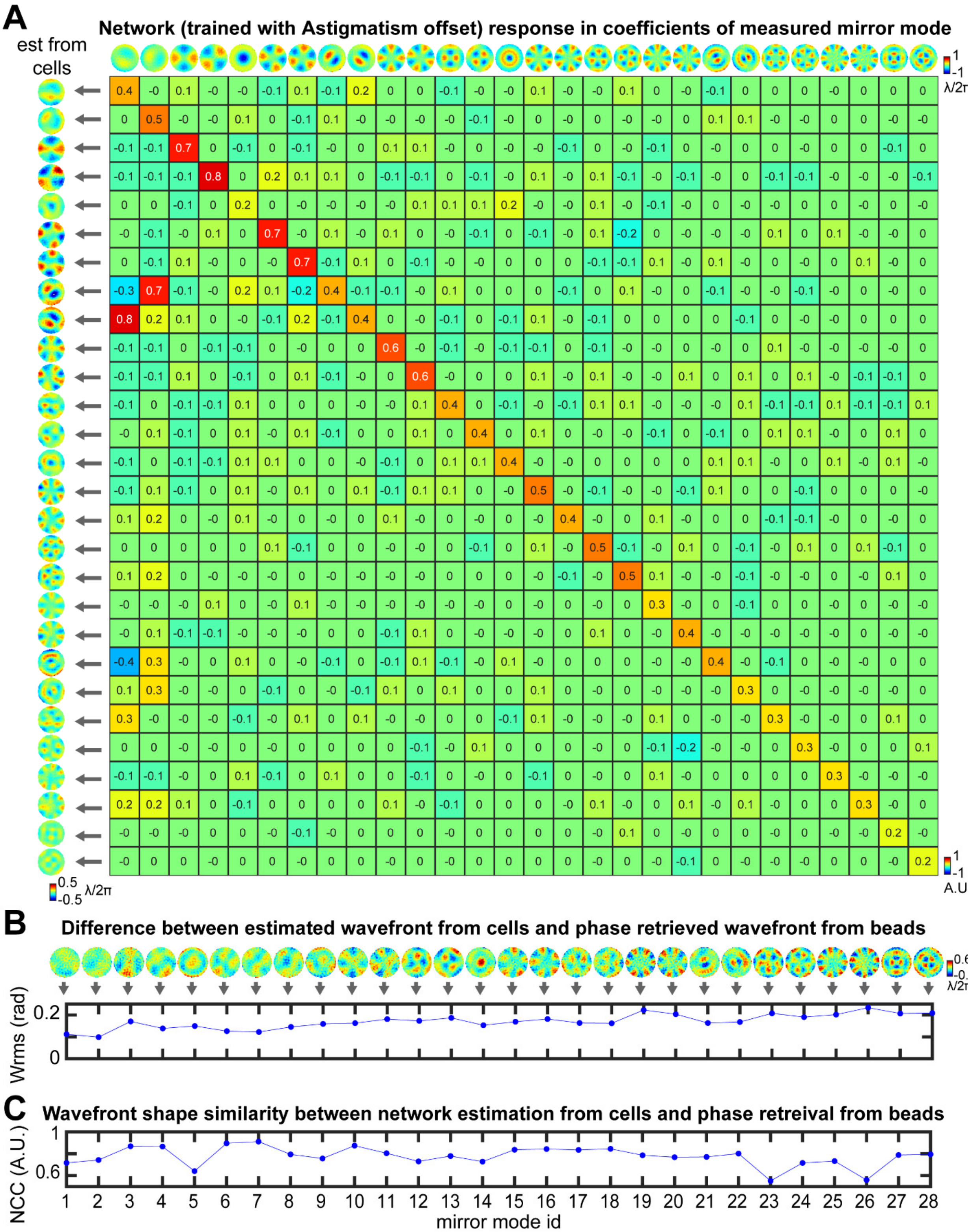
A
Network (trained with Astigmatism offset) response in coefficients of measured mirror mode
est from cells
λ/2π
A.U
B
Difference between estimated wavefront from cells and phase retrieved wavefront from beads
Wrms (rad)
C
Wavefront shape similarity between network estimation from cells and phase retreival from beads
NCC (A.U.)
mirror mode id

Fig. SS12: Characterizing neural network (trained with Astigmatism offset) responses to mirror mode changes using PSFs measured from blinking molecules. (**A**) Network response to individual mirror mode changes. Each row of the response matrix shows the network responded mirror coefficients under a unit change of each mirror deformation mode. After linear combining measured mirror modes (images below the title) with network responded coefficients, we obtained network estimated wavefront shape w.r.t. individual mirror mode changes. The PSFs were measured from experimental blinking frames of immune-fluorescence-labeled Tom20 specimens. A background map estimated by the temporal median filter was subtracted from each camera frame before segmentation. The intensity of each sub-region was estimated by summing up the photon counts in each pixel, after subtracting the median map. An intensity threshold of 1500 photons was applied to the segmented subregions to filter out PSFs with low photon counts. (**B**) Difference between network estimated wavefront (left column in A) and phase retrieved from beads (left column in **Fig. SS9**). The top row shows the pixel-wise differences between wavefronts obtained from network estimation and that obtained from phase retrieval. The plot below shows the root mean square wavefront error[31] (W_{rms}, **Methods**) of each wavefront difference. (**C**) Similarity between network estimated wavefront and phase retrieved wavefront. The similarity is quantified with 2D normalized cross correlation (NCC).

7.3 Restoring Astigmatism PSFs with DL-AO

To characterize DL-AO's capacity in restoring Astigmatism PSFs, we introduced random wavefront distortions using the deformable mirror and compensated these distortions with DL-AO during SMLM experiments with immune-fluorescence-labeled Tom20 in COS-7 cells. Visualizing the raw blinking data during the correction, we found the PSFs became less distorted and similar to Astigmatism PSF even after a single compensation (**Supplementary Videos 10-11**). Since PSFs from blinking molecules have limited photons and stochastic positions, making them challenging to quantify, we further verified the PSF shape post correction by axially scanning fluorescent beads nearby the compensation areas. Through phase retrieval, we found DL-AO results are highly similar and close to the Astigmatism wavefront shape used as the instrument optimum (**Supplementary Note 7.1.1**), with a residual of 0.35 ± 0.01 rad in W_{rms} (mean ± s.t.d, N=7, **Fig. SS13**). Comparing the PSFs post DL-AO and the instrument optimum Astigmatism PSF shape, high similarities of 0.96 ± 0.01 (mean ± s.t.d, N=7) were consistently achieved, quantified by 3D normalized cross correlation (**Fig. SS13**). Besides, we observed that DL-AO is capable of

compensating significant index mismatch induced aberrations using constructed specimens from ~146 μm in thickness with water-based imaging media (**Fig. SS14**), restoring the PSFs to approach the instrument optimum.

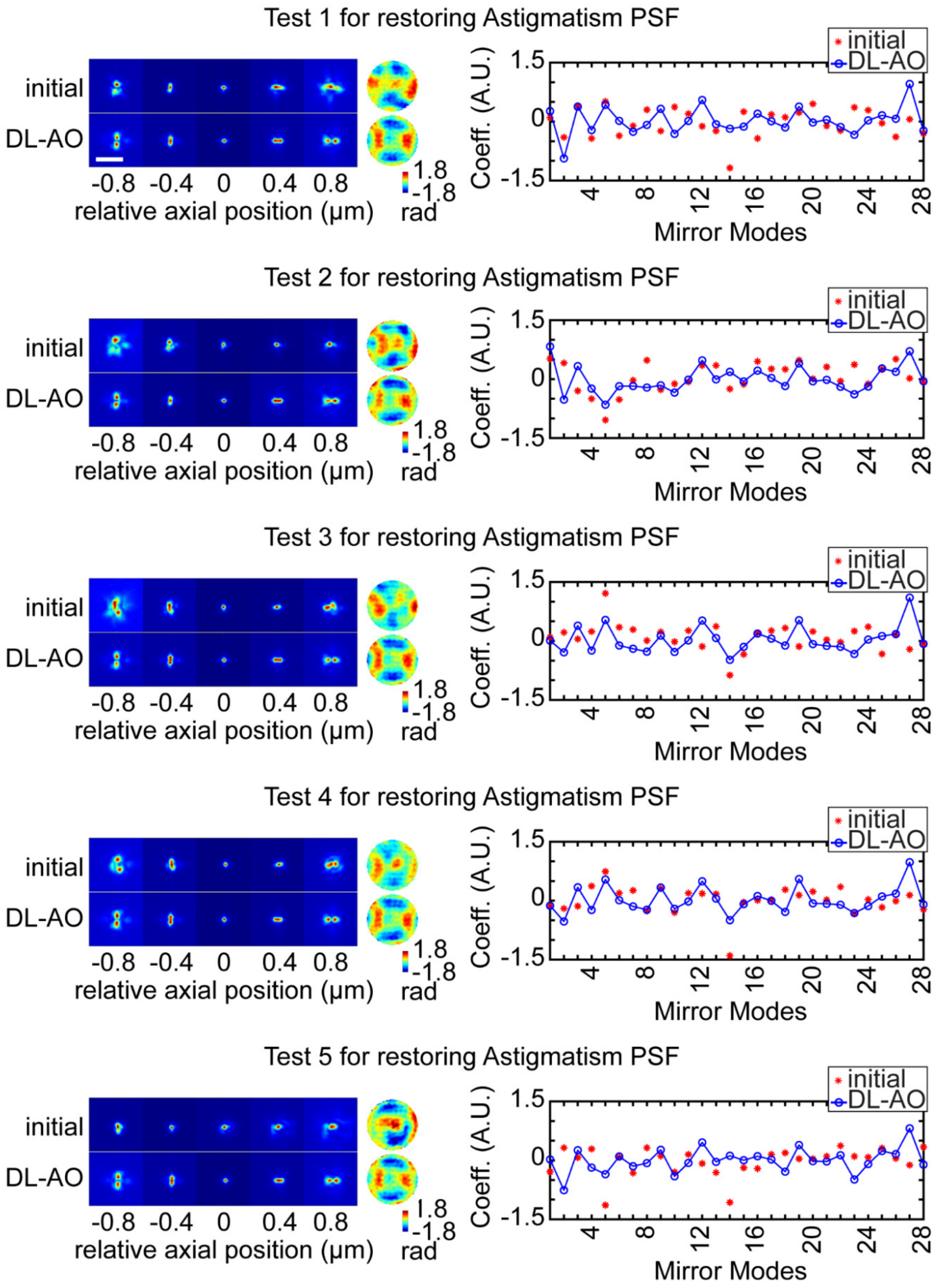
Test 1 for restoring Astigmatism PSF
initial
DL-AO
-0.8 -0.4 0 0.4 0.8
relative axial position (μm)
1.8
-1.8
rad
initial
DL-AO
1.5
0
-1.5
Coeff. (A.U.)
4 8 12 16 20 24 28
Mirror Modes
Test 2 for restoring Astigmatism PSF
initial
DL-AO
-0.8 -0.4 0 0.4 0.8
relative axial position (μm)
1.8
-1.8
rad
initial
DL-AO
1.5
0
-1.5
Coeff. (A.U.)
4 8 12 16 20 24 28
Mirror Modes
Test 3 for restoring Astigmatism PSF
initial
DL-AO
-0.8 -0.4 0 0.4 0.8
relative axial position (μm)
1.8
-1.8
rad
initial
DL-AO
1.5
0
-1.5
Coeff. (A.U.)
4 8 12 16 20 24 28
Mirror Modes
Test 4 for restoring Astigmatism PSF
initial
DL-AO
-0.8 -0.4 0 0.4 0.8
relative axial position (μm)
1.8
-1.8
rad
initial
DL-AO
1.5
0
-1.5
Coeff. (A.U.)
4 8 12 16 20 24 28
Mirror Modes
Test 5 for restoring Astigmatism PSF
initial
DL-AO
-0.8 -0.4 0 0.4 0.8
relative axial position (μm)
1.8
-1.8
rad
initial
DL-AO
1.5
0
-1.5
Coeff. (A.U.)
4 8 12 16 20 24 28
Mirror Modes

Fig. SS13: DL-AO restores Astigmatism PSF from artificially induced aberrations. Compensations are performed in real time during SMLM experiment based on experimental blinking frames from immune-fluorescence-labeled Tom20 specimens. A background map estimated by the temporal median filter was subtracted from each camera frame before segmentation. The intensity of each sub-region was estimated by summing up the photon counts in each pixel, after subtracting the median map. An intensity threshold of 1500 photons was applied to the segmented subregions to filter out PSFs with low photon counts. Five examples of PSFs, pupil phases and mirror mode coefficients before and after DL-AO are shown in this figure. The artificial aberrations are induced at 0.5 radian level. PSFs are measured from 100-nm-diameter crimson beads nearby the compensation area post SMLM acquisition. See Supplementary Video 10-11 for the compensation process.

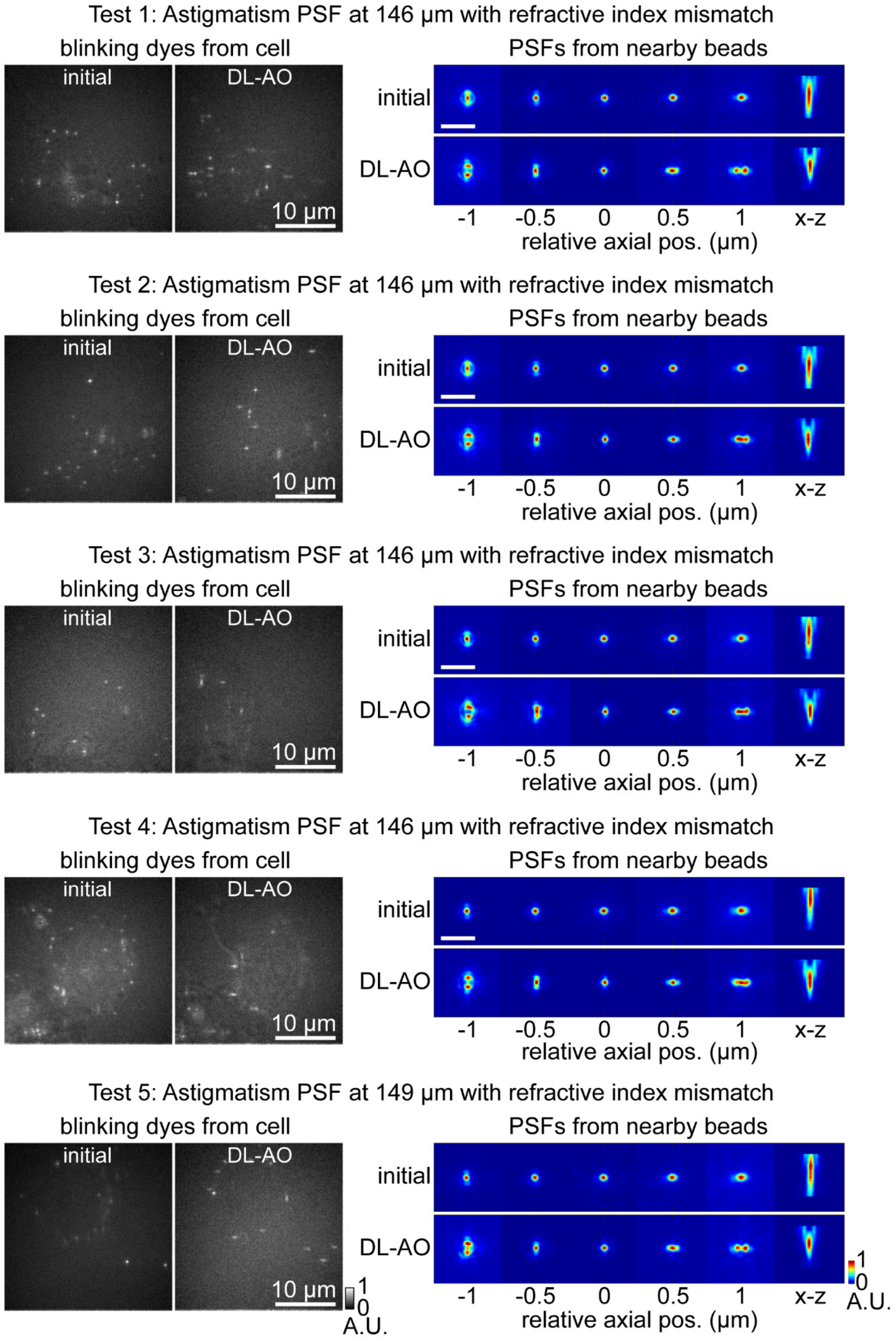
Test 1: Astigmatism PSF at 146 μm with refractive index mismatch
blinking dyes from cell
PSFs from nearby beads
initial
DL-AO
10 μm
-1
-0.5
0
0.5
1
x-z
relative axial pos. (μm)
Test 2: Astigmatism PSF at 146 μm with refractive index mismatch
blinking dyes from cell
PSFs from nearby beads
initial
DL-AO
10 μm
-1
-0.5
0
0.5
1
x-z
relative axial pos. (μm)
Test 3: Astigmatism PSF at 146 μm with refractive index mismatch
blinking dyes from cell
PSFs from nearby beads
initial
DL-AO
10 μm
-1
-0.5
0
0.5
1
x-z
relative axial pos. (μm)
Test 4: Astigmatism PSF at 146 μm with refractive index mismatch
blinking dyes from cell
PSFs from nearby beads
initial
DL-AO
10 μm
-1
-0.5
0
0.5
1
x-z
relative axial pos. (μm)
Test 5: Astigmatism PSF at 149 μm with refractive index mismatch
blinking dyes from cell
PSFs from nearby beads
initial
DL-AO
10 μm
1
0
A.U.
-1
-0.5
0
0.5
1
x-z
relative axial pos. (μm)
1
0
A.U.

Fig. SS14: DL-AO restores Astigmatism PSF from index mismatch induced aberrations. The SMLM blinking frames for compensation were acquired from immune-fluorescence-labeled Tom20 specimen at ~146 μm from bottom coverslip surface in water-based media (n = 1.35). The results shown are representative of 5 trials. A background map estimated by the temporal median filter was subtracted from each camera frame before segmentation. The intensity of each sub-region was estimated by summing up the photon counts in each pixel, after subtracting the median map. An intensity threshold of 1500 photons was applied to the segmented subregions to filter out PSFs with low photon counts. PSFs were measured from 100-nm-diameter crimson beads nearby the compensation area post SMLM acquisition. Imaging depths were measured by the differences in PIFOC readings between the apparent focus of the region-of-interest and the bottom coverslip surface. Scale bar on PSFs from fluorescent beads: 2 μm. See **Supplementary Video 12** for the compensation process.

7.4 Robustness of DL-AO for Astigmatism-based setup

Next, we evaluated the robustness of DL-AO on compensating different levels of wavefront distortion, from 0.25 to 2 radians in W_{rms}. We observed that PSFs were restored to approach the instrument optimum Astigmatism shape, with a similarity of 0.96 ± 0.01 (mean ± s.t.d, N=15 in NCC, examples shown in **Fig. SS15**). By assessing the residual wavefront error post correction using both simulated data and experimental single molecule blinking data, we observed a residual level at 0.37 ± 0.1 radians (mean ± s.t.d, N=135) for simulated data (**Fig. SS16A**), and at 0.48 ± 0.26 radians (mean ± s.t.d, N=120) of the induced level was compensated for experimental data (**Fig. SS16B**) after 19 mirror updates.

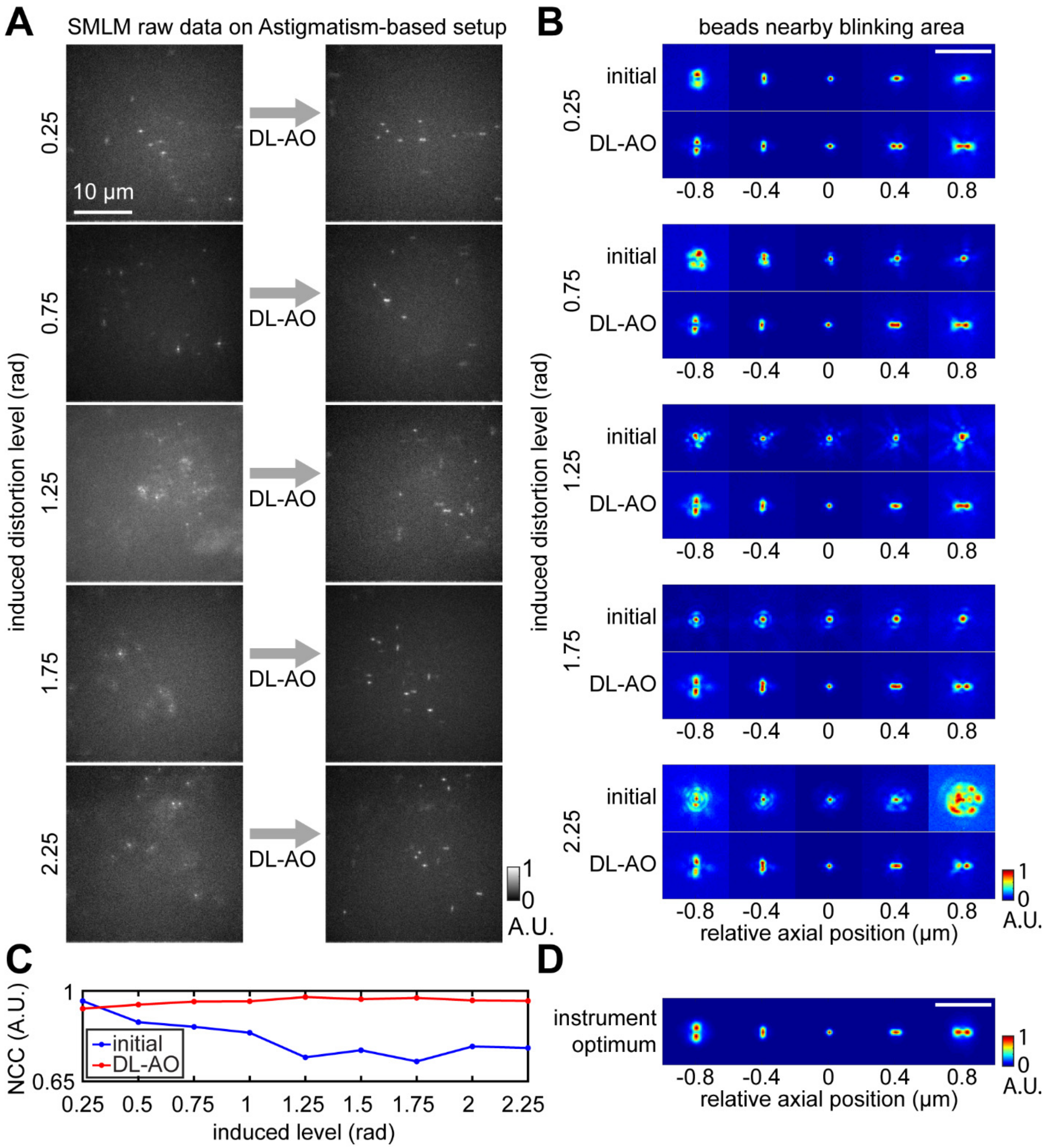

Fig. SS15: Examples of Astigmatism PSFs before and after DL-AO at various amounts of induced aberrations. (**A**) SMLM frames of Astigmatism setup before and after DL-AO compensating various amounts of induced aberrations. DL-AO was compensating aberrations at different levels (in W_{rms}, **Methods**) based on experimental blinking frames from immune-fluorescence-labeled Tom20 specimens. 100 camera frames were used for DL-AO estimation before each mirror update. A background map estimated by the temporal median filter was subtracted from each camera frame before segmentation. The intensity of each sub-region was estimated by summing up the photon counts in each pixel, after subtracting the median map. An intensity threshold of 1500 photons was applied to the segmented subregions to filter out

PSFs with low photon counts. The blinking data after DL-AO were the compensation results after 19 mirror updates. The results shown are representative of 5 trials. (**B**) Examples of Astigmatism PSFs before and after DL-AO, when compensating artificially induced aberrations. Compensations are performed in real time during SMLM experiments shown in A. PSFs are measured from 100-nm-diameter crimson beads nearby the compensation area post SMLM acquisition. Scale bar: 5 μm. (**C**) Quantitative comparisons between PSFs measured under instrument optimum and those measured before and after DL-AO using 3D normalized cross correlation (NCC). 31 PSFs from -1.5 μm to 1.5 μm axial positions were used to calculated the normalized cross correlation. (**D**) PSFs are simulated using pupil measured under instrument optimum (**Methods**) with an +1.5 (A.U.) Mirror Mode 3 (**Fig. SS10**) offset to mimic Astigmatism-based setup. Scale bar: 5 μm.

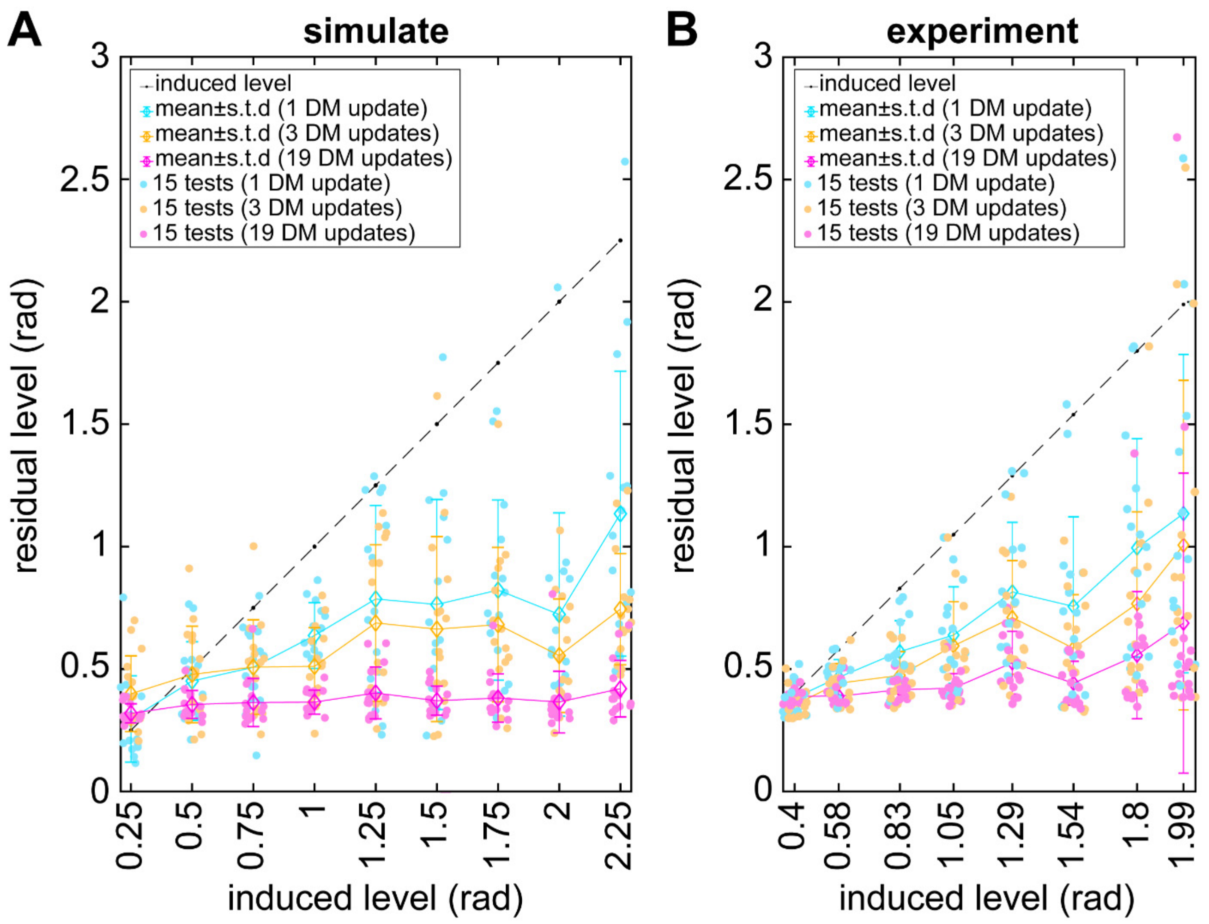

Fig. SS16: Repeated tests of DL-AO in Astigmatism-based setup. (**A**) Summary of repeated tests of DL-AO for compensating aberrations of different levels (in W_{rms}) based on simulated SMLM blinking data. Each simulated SMLM frames contain 128×128 pixels, with pixel size of 119 nm. Number of PSFs per frame were generated from Poisson distribution with a mean of 13. Axial positions of molecules were generated from uniform distribution from -1 to 1 μm range. The number of photon counts in each PSF was generated from exponential distribution with mean equal to 2500. The number of background photon counts in each frame was set to be 10. (**C**) Summary of repeated tests of DL-AO for compensating aberrations in different levels (in W_{rms}) based on experimental blinking frames from immune-fluorescence-labeled Tom20 specimen. A background map estimated by the temporal median filter was subtracted from each camera frame before segmentation. The intensity of each sub-region were estimated by summing up the photon counts in each pixel, after subtracting the median map. An intensity threshold of 1500 photons were applied to the segmented subregions to filter out PSFs with low photon counts.

7.5 Discussion about adapting DL-AO for other 3D imaging modalities

We have demonstrated that DL-AO is capable of restoring PSFs in biplane setup and Astigmatism-based setup, two commonly used 3D imaging modalities in SMLM. PSF engineering approaches[42] have been developed to encode 3D position information of a molecule into PSF shape with extended imaging capacities, such as axial imaging volume. These approaches require using more complexed phase mask in the optical system. To adapt DL-AO in such complex system, it is important to check whether there is degeneracy problem[3,22], i.e. the situation where different aberrations correspond to the same PSF shape. In the case where degeneracy is unavoidable, an potential solution is to electronically/mechanically remove the phase mask during compensation, and restore the phase mask after DL-AO.

8. Investigation on controlling 50 modes simultaneously with DL-AO

8.1 Modifications in DL-AO for controlling 50 modes simultaneously

We further investigated whether DL-AO can be upgraded to simultaneously estimate and compensate more complex aberration shapes using 50 mirror modes. In these tests, the optical setup (**Methods**) and DL-AO workflow (**Supplementary Note 2**) remain unchanged.

In the current demonstration, we only changed the output size from 28 to 50 in the network architecture (**Supplementary Table 2**). We expect that future development in designing training data and neural network architecture will improve the inference accuracy of DL-AO through a large compensation range. The experimental mirror deformations (**Fig. SS17**) in optical system were measured using the same procedure as described in **Supplementary Note 3.3**. Each PSF was generated following the process described in **Supplementary Note 4.2**. The only change is

to replace $(\varphi_{M1}, \varphi_{M2} \dots, \varphi_{M28})$ by $(\varphi_{M1}, \varphi_{M2} \dots, \varphi_{M50})$ in Equations (1) to (3). The variation ranges of training parameters were documented in **Table SS2**.

Table SS2: Variation range of parameters in training data generation for controlling 50 mirror modes

Parameters	Range of uniform distributions		
	Network1	Network2	Network3
Mirror Mode 1 and 2^{+}	[-1, 1]	[-2, 2]	[-0.25, 0.25]
Mirror Mode 3 and 4	[-1, 1]	[-1, 1]	[-0.25, 0.25]
Mirror Mode 5	[-20, 20]	[-1, 1]	[-0.25, 0.25]
Mirror Mode 6-13	[-1, 1]	[-1, 1]	[-0.25, 0.25]
Mirror Mode 14	[-5, 5]	[-1, 1]	[-0.25, 0.25]
Mirror Mode 15-50	[-1, 1]	[-1, 1]	[-0.25, 0.25]
Photon counts per PSF	[1000, 20000] counts		
Photon counts in detection plane 2 ÷ Photon counts in detection plane 1	[0.9, 1.5]		
Background photon counts per detection plane	[1, 300] counts		
Background photon counts in detection plane 2 ÷ Background photon counts in detection plane 1	[0.9, 1.5]		
PSF position relative to sub-region center (x and y)	[-3, 3] pixels**		
Lateral shift of PSF in detection plane 2 relative to plane 1*	[-1.5, 1.5] pixels		
Molecule's axial position relative to focus*	[-2, 2] μm		
Axial distance between two detection plane	[0.298, 0.426] μm		

$^{+}$ Mirror mode 1-50 can have different shapes and levels in W_{rms} for different optical systems. The coefficient values here are the scaling factors for deformable mirror voltage control. They have arbitrary units. Their actual influences can be estimated by linear combining of measured mirror modes (**Fig. SS17**) then calculating W_{rms} of the composed wavefront. This estimation is accurate only when mirror deforms linearly with input voltages.

* Focus of biplane setup here is defined as the axial position where the PSFs in two detection planes look most similar.

** pixel size is 119 nm.

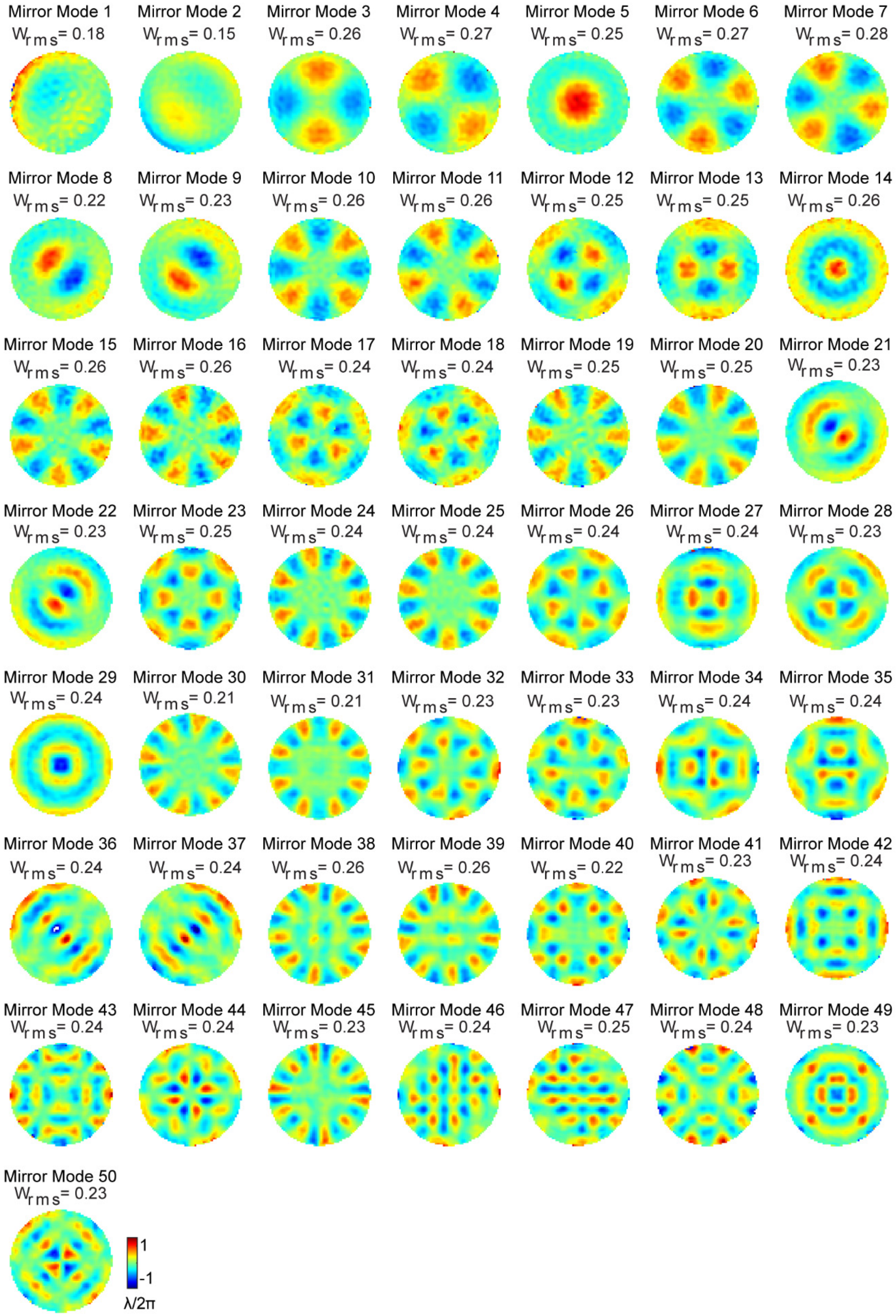
Mirror Mode 1
Wrms= 0.18
Mirror Mode 2
Wrms= 0.15
Mirror Mode 3
Wrms= 0.26
Mirror Mode 4
Wrms= 0.27
Mirror Mode 5
Wrms= 0.25
Mirror Mode 6
Wrms= 0.27
Mirror Mode 7
Wrms= 0.28
Mirror Mode 8
Wrms= 0.22
Mirror Mode 9
Wrms= 0.23
Mirror Mode 10
Wrms= 0.26
Mirror Mode 11
Wrms= 0.26
Mirror Mode 12
Wrms= 0.25
Mirror Mode 13
Wrms= 0.25
Mirror Mode 14
Wrms= 0.26
Mirror Mode 15
Wrms= 0.26
Mirror Mode 16
Wrms= 0.26
Mirror Mode 17
Wrms= 0.24
Mirror Mode 18
Wrms= 0.24
Mirror Mode 19
Wrms= 0.25
Mirror Mode 20
Wrms= 0.25
Mirror Mode 21
Wrms= 0.23
Mirror Mode 22
Wrms= 0.23
Mirror Mode 23
Wrms= 0.25
Mirror Mode 24
Wrms= 0.24
Mirror Mode 25
Wrms= 0.24
Mirror Mode 26
Wrms= 0.24
Mirror Mode 27
Wrms= 0.24
Mirror Mode 28
Wrms= 0.23
Mirror Mode 29
Wrms= 0.24
Mirror Mode 30
Wrms= 0.21
Mirror Mode 31
Wrms= 0.21
Mirror Mode 32
Wrms= 0.23
Mirror Mode 33
Wrms= 0.23
Mirror Mode 34
Wrms= 0.24
Mirror Mode 35
Wrms= 0.24
Mirror Mode 36
Wrms= 0.24
Mirror Mode 37
Wrms= 0.24
Mirror Mode 38
Wrms= 0.26
Mirror Mode 39
Wrms= 0.26
Mirror Mode 40
Wrms= 0.22
Mirror Mode 41
Wrms= 0.23
Mirror Mode 42
Wrms= 0.24
Mirror Mode 43
Wrms= 0.24
Mirror Mode 44
Wrms= 0.24
Mirror Mode 45
Wrms= 0.23
Mirror Mode 46
Wrms= 0.24
Mirror Mode 47
Wrms= 0.25
Mirror Mode 48
Wrms= 0.24
Mirror Mode 49
Wrms= 0.23
Mirror Mode 50
Wrms= 0.23
1
-1
λ/2π

Fig. SS17: Measured mirror modes in optical setup for controlling 50 modes. The mirror modes were measured with phase retrieval on 100-nm-diameter crimson beads (**Methods**). Each mirror deformation mode is generated by introducing a unit change in mirror mode voltage control. The level of distortion introduced by each mirror mode is estimated by calculating the root mean square wavefront error (W_{rms}, **Methods**) of each phase retrieved wavefront. The unit of W_{rms} is λ/2π. Mirror Mode 1-28 looks different from the corresponding Mirror Modes in **Fig. SS6** due to system modification.

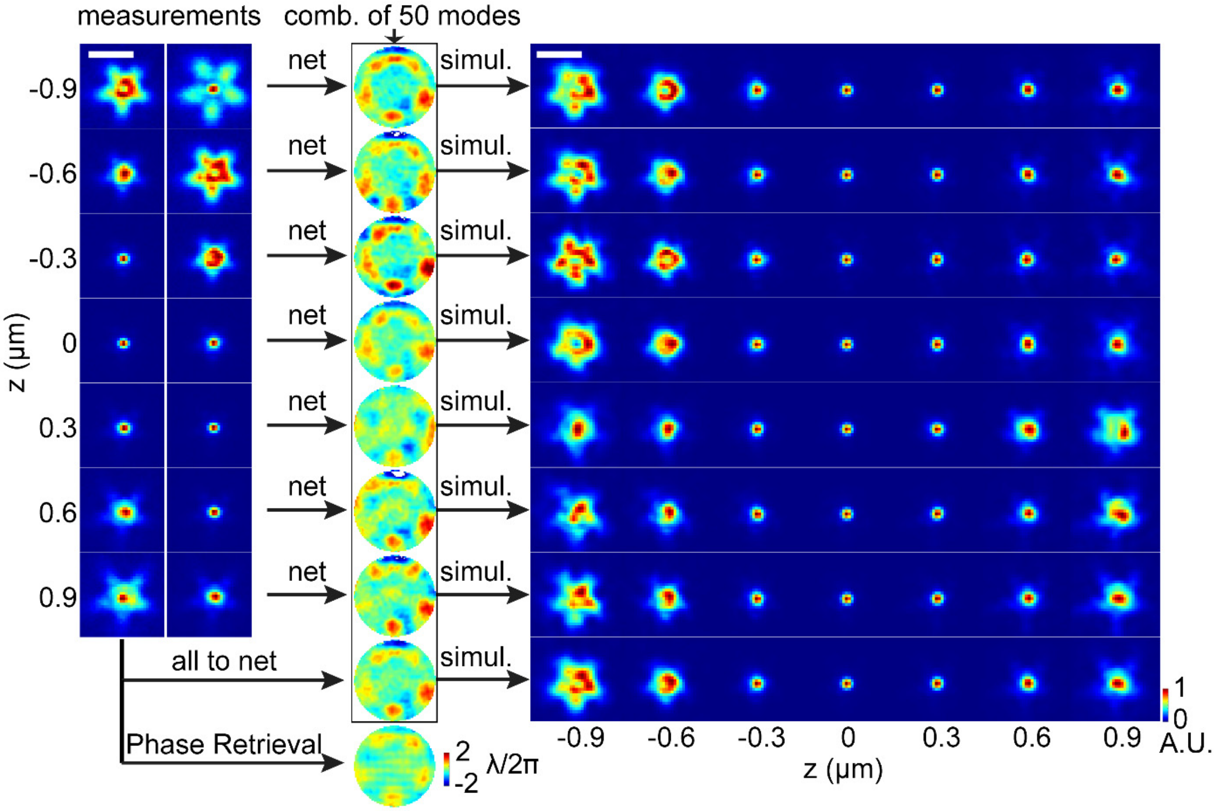
measurements
comb. of 50 modes
net
simul.
all to net
Phase Retrieval
-0.9
-0.6
-0.3
0
0.3
0.6
0.9
z (μm)
2
-2
λ/2π
1
0
A.U.

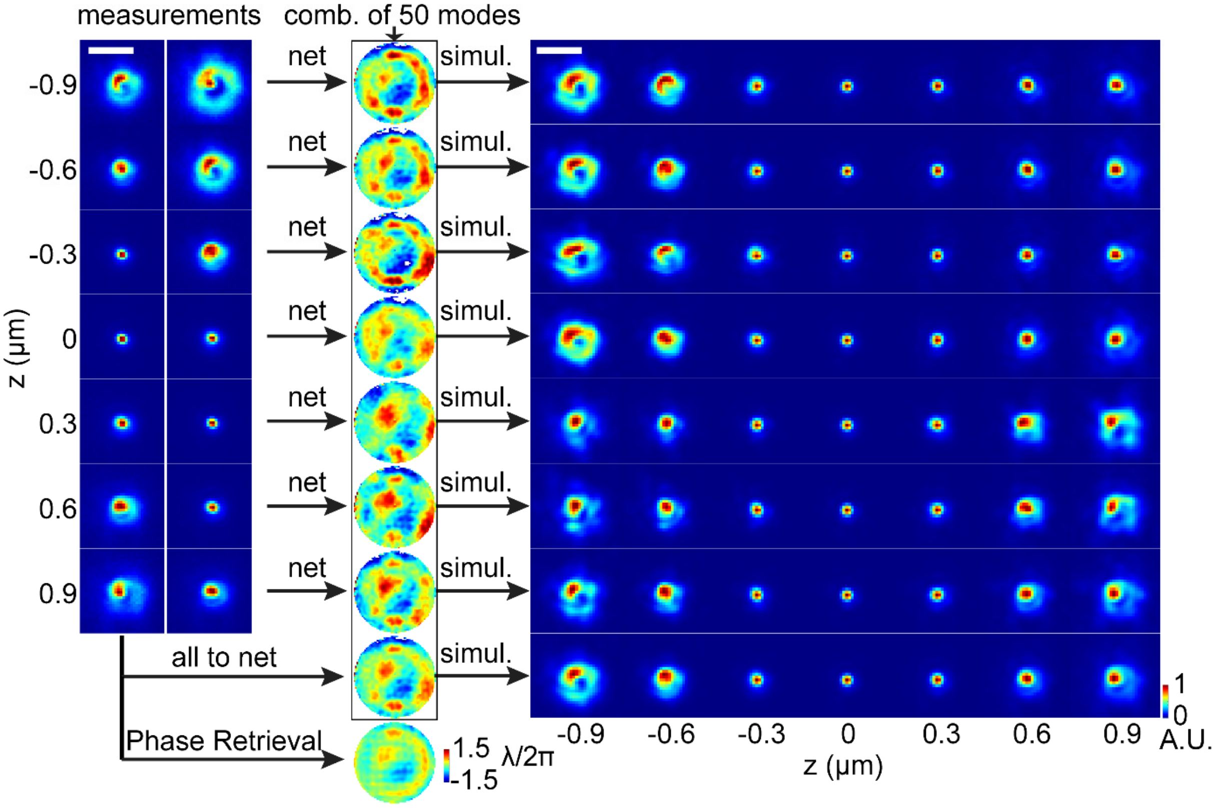
measurements
comb. of 50 modes
net
simul.
all to net
Phase Retrieval
-0.9
-0.6
-0.3
0
0.3
0.6
0.9
z (μm)
1.5
-1.5
λ/2π
1
0
A.U.

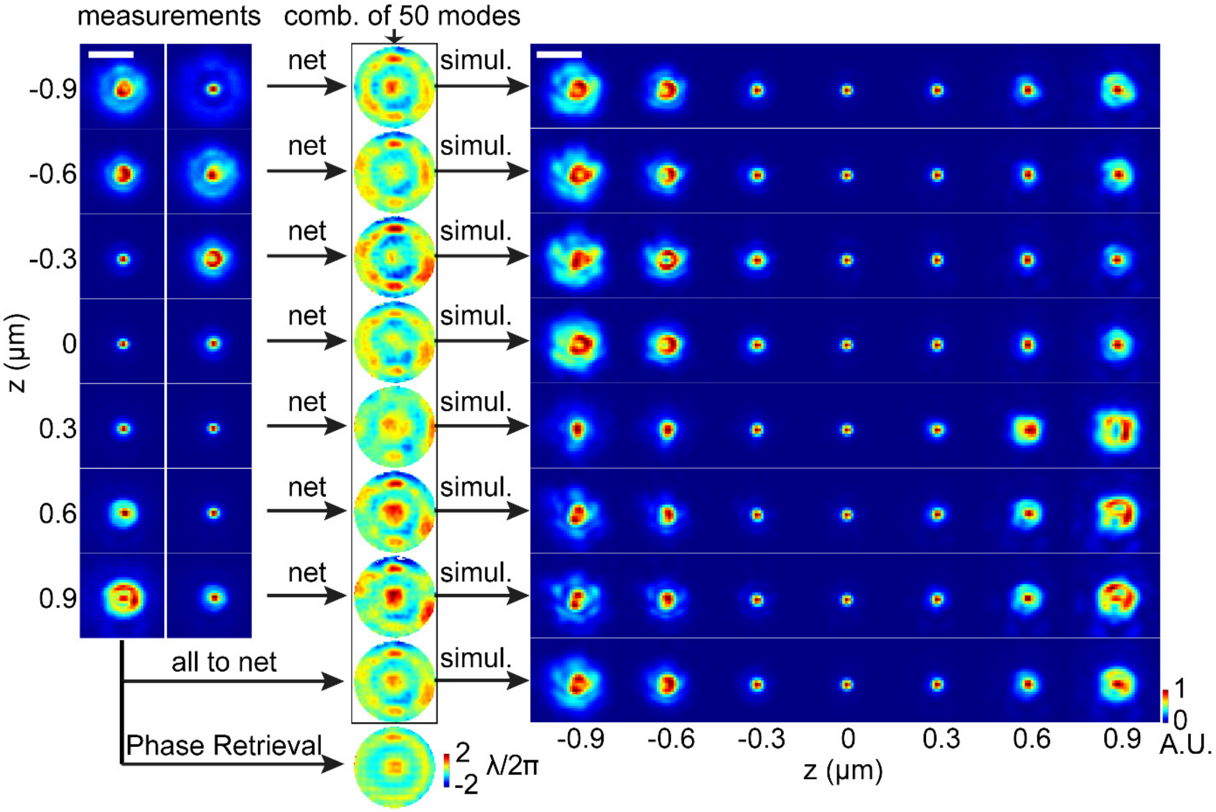

Fig. SS18: Comparison between measured PSFs and PSFs simulated from network estimations based on a single measurement of an isolated molecule. The left column shows measured PSFs from 100-nm-diameter crimson beads when scanning Piezo stage at different axial positions. The measured PSFs in biplane sub-regions were sent to neural network, which outputs a vector of mirror mode coefficients for each sub-region. The 50 measured mirror modes (**Fig. SS17**) were linear combined with mirror mode coefficients output from network, which result in wavefronts shown in the middle column (within the black box). The wavefront for network estimations based on all measured PSFs (the bottom one inside the black box) were generated with an averaged value among network outputs w.r.t. PSFs at different axial positions. The wavefront outside the black box is obtained by phase retrieval[11]. The network estimated wavefronts were then used to simulate PSFs at different axial positions to check the similarity between measured PSFs and PSFs simulated from network estimations. PSFs were simulated without background and noise for visualization. Scale bars: 2 μm.

8.2 Response accuracy of DL-AO network to 50 mirror modes changes

We first characterized the response accuracy of DL-AO network using controlled wavefront distortions generated by the deformable mirror. These wavefront distortions resulted in aberrated emission patterns, which were then collected and sent to DL-AO network (**Methods**). By

comparing the induced deformation amplitudes with those estimated by DL-AO, we observed that DL-AO network responded towards individual mirror deformations mostly in a one-to-one manner. This behavior was consistently observed with both beads samples and blinking single molecules from immune-fluorescence-labeled cell specimens (**Figs. SS19-20**). We observed that independent measurements from DL-AO and phase retrieval[10,32] using PSFs of fluorescent beads resulted in similar wavefront shapes with a small difference of 0.12 ± 0.03 rad (mean ± s.t.d, N=50) quantified in root mean square wavefront error[31] (W_{rms}, **Methods**, **Fig. SS19A-B**). Further, comparing the wavefronts estimated by DL-AO network using single molecule blinking data to that retrieved by phase retrieval from beads, we observed high similarities of 0.70 ± 0.14 (mean ± s.t.d, N=50, normalized cross correlation), and a wavefront difference of 0.17 ± 0.03 rad (mean ± s.t.d, N=50) in Wrms (**Fig. SS20**).

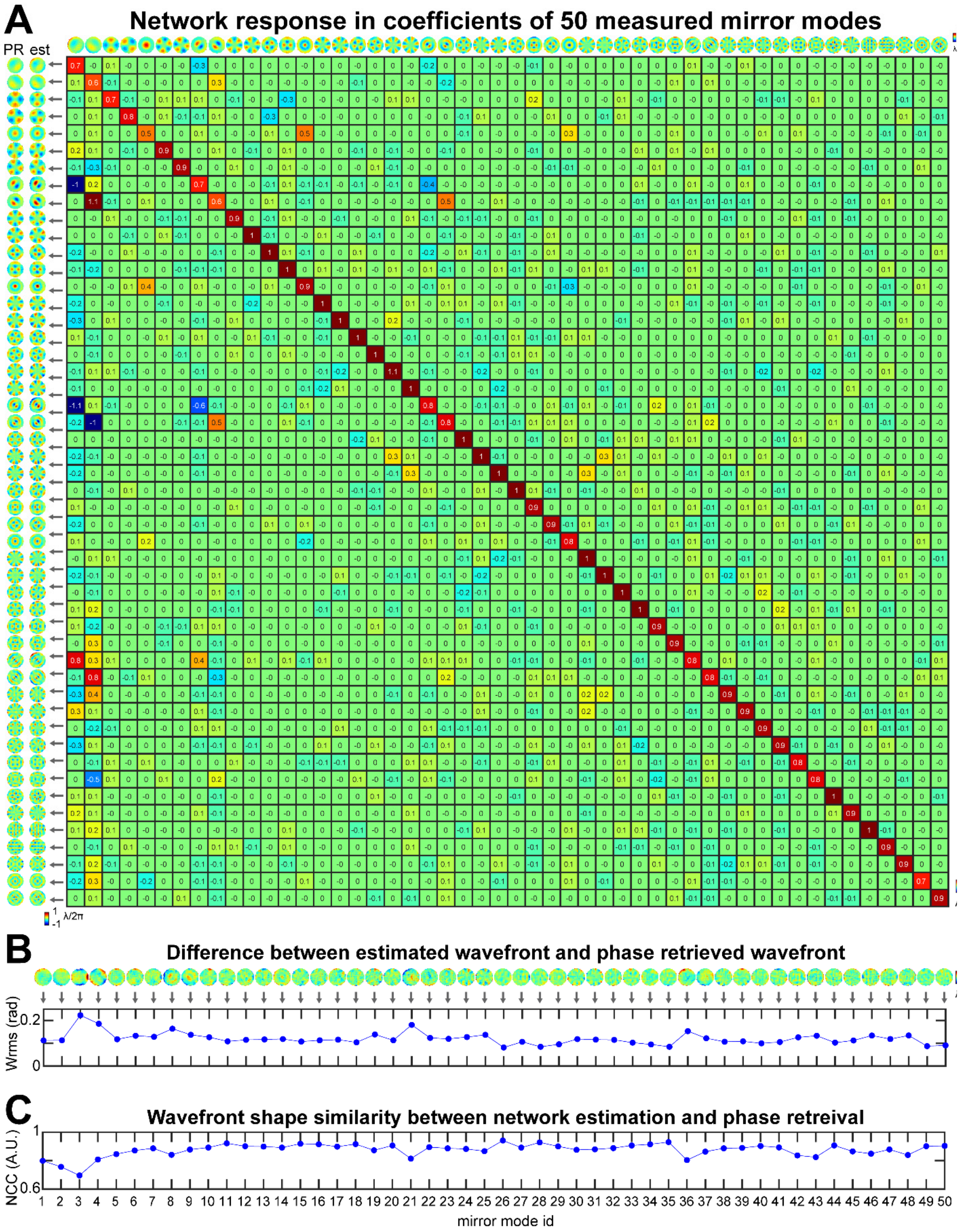
A
Network response in coefficients of 50 measured mirror modes
PR est
λ/2π
B
Difference between estimated wavefront and phase retrieved wavefront
Wrms (rad)
0.2
0
C
Wavefront shape similarity between network estimation and phase retreival
NCC (A.U.)
1
0.6
1 2 3 4 5 6 7 8 9 10 11 12 13 14 15 16 17 18 19 20 21 22 23 24 25 26 27 28 29 30 31 32 33 34 35 36 37 38 39 40 41 42 43 44 45 46 47 48 49 50
mirror mode id

Fig. SS19: Characterizing neural network responses to 50 mirror mode changes using PSFs measured from fluorescent beads. (**A**) Network response to individual mirror mode changes. Each row of the response matrix shows the network responded mirror coefficients under a unit change of each mirror deformation mode. After linear combining measured mirror modes (images below the title) with network responded coefficients, we obtained network estimated wavefront shape w.r.t. individual mirror mode changes (the 2nd column). The 1st column shows phase retrieved wavefronts from beads imaged individual mirror mode changes. The PSFs were measured with 100-nm-diameter crimson beads. PSFs from -1.5 μm to 1.5 μm around the focus, with 0.1 μm step size, were collected for characterizing network responses. (**B**) Difference between network estimated wavefront and phase retrieved wavefront (the first two columns in A). The top row shows the pixel-wise differences between wavefronts obtained from network estimation and that obtained from phase retrieval. The plot below shows the root mean square wavefront error[31] (W_{rms}, **Methods**) of each wavefront difference. (**C**) Similarity between network estimated wavefront and phase retrieved wavefront. The similarity is quantified with 2D normalized cross correlation (NCC).

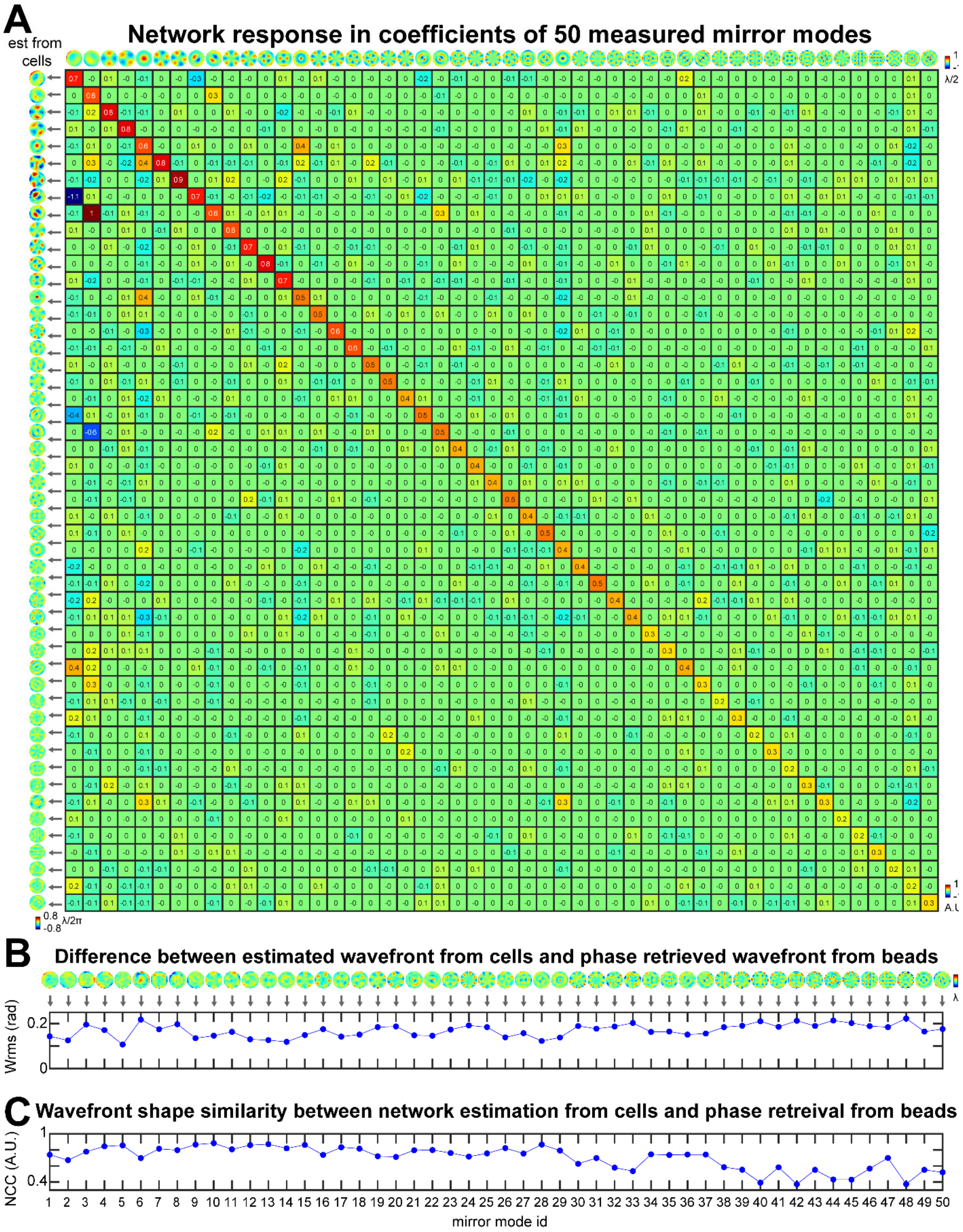
A
Network response in coefficients of 50 measured mirror modes
est from cells
λ/2π
A.U.
B
Difference between estimated wavefront from cells and phase retrieved wavefront from beads
Wrms (rad)
C
Wavefront shape similarity between network estimation from cells and phase retreival from beads
NCC (A.U.)
mirror mode id

Fig. SS20: Characterizing neural network responses to 50 mirror mode changes using PSFs measured from blinking molecules. (**A**) Network response to individual mirror mode changes. Each row of the response matrix shows the network responded mirror coefficients under a unit change of each mirror deformation mode. After linear combining measured mirror modes (images below the title) with network responded coefficients, we obtained network estimated wavefront shape w.r.t. individual mirror mode changes. The PSFs were experimental blinking frames measured from immune-fluorescence-labeled Tom20 specimen. A background map estimated by the temporal median filter was subtracted from each camera frame before segmentation. The intensity of each sub-region was estimated by summing up the photon counts in each pixel, after subtracting the median map. An intensity threshold of 2500 photons was applied to the segmented subregions to filter out PSFs with low photon counts. (**B**) Difference between network estimated wavefront (left column in A) and phase retrieved from beads (left column in **Fig. SS19**). The top row shows the pixel-wise differences between wavefronts obtained from network estimation and that obtained from phase retrieval. The plot below shows the root mean square wavefront error[31] (W_{rms}, **Methods**) of each wavefront difference. (**C**) Similarity between network estimated wavefront and phase retrieved wavefront. The similarity is quantified with 2D normalized cross correlation (NCC).

8.3 Restoring PSFs using 50 modes

To characterize DL-AO's capacity in restoring PSFs when controlling 50 mirror modes, we introduced random wavefront distortions using the deformable mirror and compensated these distortions with DL-AO during SMLM experiments with immune-fluorescence-labeled Tom20 in COS-7 cells. We verified the PSF shape post correction by axially scanning fluorescent beads nearby the compensation areas. We observed that PSFs post DL-AO and the instrument optimum PSF shape have similarities of 0.96 ± 0.02 (mean ± s.t.d, N=22), quantified by 3D normalized cross correlation (**Fig. SS21**).

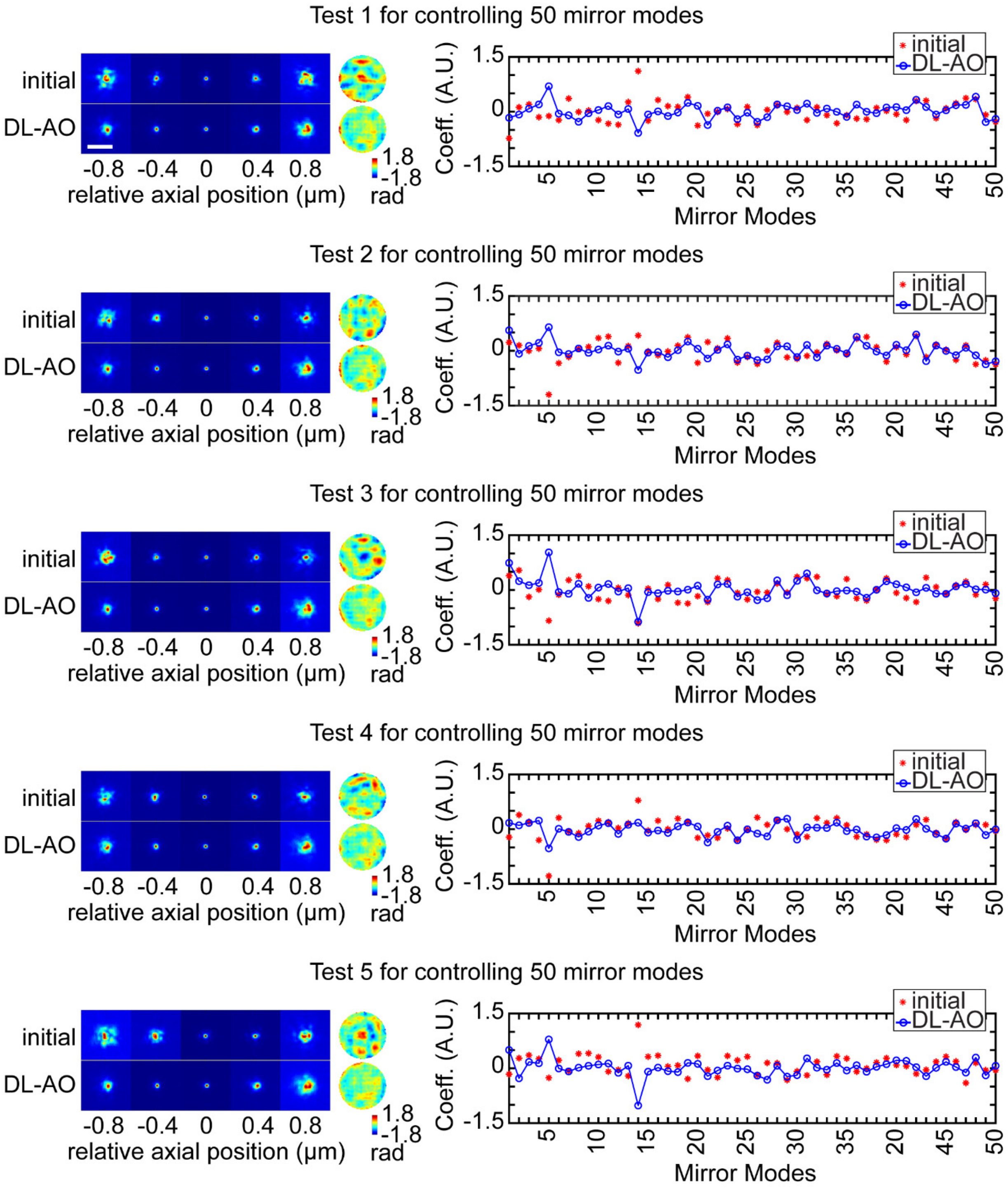

Fig. SS21: DL-AO simultaneously controls 50 mirror modes to restore PSFs from artificially induced aberrations. Compensations are performed in real time during SMLM experiment based on experimental blinking frames from immune-fluorescence-labeled Tom20 specimens. A background map estimated by the temporal median filter was subtracted from each camera frame before segmentation. The intensity of each sub-region was estimated by summing up the photon counts in each pixel, after subtracting the median map. An intensity

threshold of 2500 photons was applied to the segmented subregions to filter out PSFs with low photon counts. Five examples of PSFs, pupil phases and mirror mode coefficients before and after DL-AO are shown in this figure. The artificial aberrations are induced at 0.5 radian level. PSFs are measured from 100-nm-diameter crimson beads nearby the compensation area post SMLM acquisition.

9. Discussion about imaging tissue sections

9.1 Challenges in comparing resolution among super resolution techniques

Super-resolution microscopies such as Stimulated Emission Depletion Microscopy (STED)[43], Structured Illumination Microscopy (SIM)[44], and Single Molecule Localization Microscopy (SMLM)[45–47] are parallel approaches that break the fundamental diffraction limit of light using different principles. The unique advantage of SMLM lies in measuring individual molecules without ensemble averaging[42,48], its potential in achieving ultra-high resolution in both live and fixed specimens, and molecular counting. Further improving the resolution and imaging depths of SIM, STED, and SMLM in cells and tissues remain ongoing research fields. There are review articles[49,50] about this cross-modality comparison between these super resolution techniques. However, in practice, it can be challenging to complete in tissue specimens. One of the major challenges of super-resolution imaging through tissues are sample and labeling optimizations. In our experience, each new type of tissue specimens requires up to 3-month to 1-year optimization before it is viable for single molecule detection and able to provide quantifiable data (similar to those shown in the manuscript). Optimizing the labeling for the same epitope in tissues for both SMLM and STED are further challenged by the opposite requirement of photophysical property of these technologies. Among many other requirements, STED requires photostable probe while SMLM require photo switchable (or blinking) dyes. An attempted comparison between these

modalities on the same specimen would potentially lead to a measurement on sample or probe compatibility rather than the technically achieved resolution between these modalities.

9.2 Challenges in whole slice reconstruction and vision for future development

The ability to image through thicker sections will also allow us to correlate super resolution structural imaging with functional imaging of the brain. However, imaging the entire brain section is yet to be feasible at the current stage. Regardless of the fluorescent background and the acquisition time for scanning through the entire brain section, we were not able to observe continuous signals throughout the brain section. We expect that this is mainly due to the staining methods we have used. Recently developed tissue clearing technologies would be extremely important here in allowing homogenous labeling throughout the tissue depths. Combing the developed DL-AO approach with optically cleared tissues would be an exciting step forward allowing ultra-structures of tissue constituents to be imaged at nanometer scale. The SMLM field is moving towards scanning through a whole brain section, with advancements in adaptive optics, high throughput imaging, light sheet, and tissue clearing. We hope DL-AO can be an indispensable tool in this promising future of SMLM.

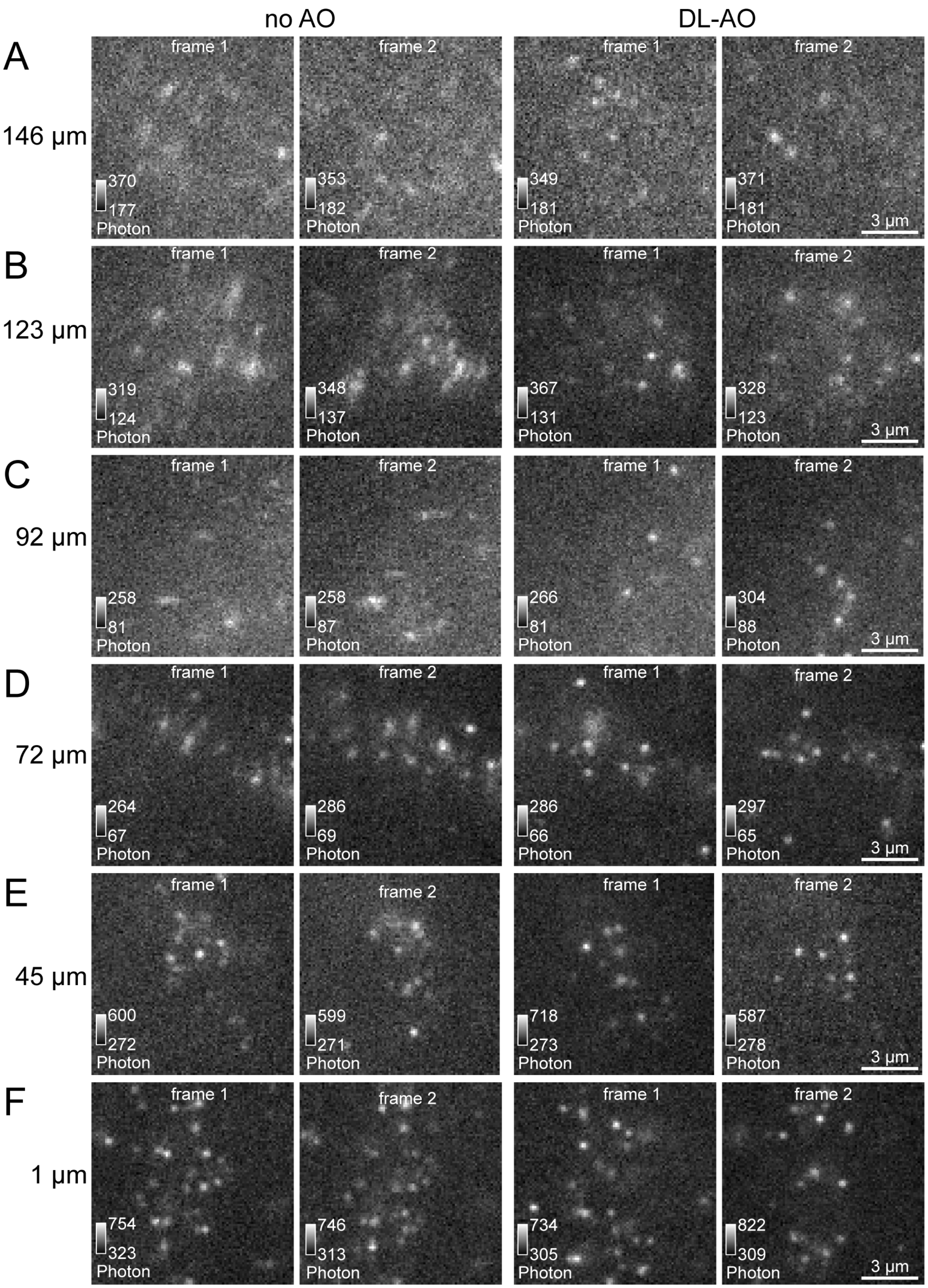

no AO
DL-AO
A
146 μm
B
123 μm
C
92 μm
D
72 μm
E
45 μm
F
1 μm
frame 1
frame 2
Photon
3 μm
370
177
353
182
349
181
371
181
319
124
348
137
367
131
328
123
258
81
258
87
266
81
304
88
264
67
286
69
286
66
297
65
600
272
599
271
718
273
587
278
754
323
746
313
734
305
822
309

Fig. SS22: Representative images of raw data before and after DL-AO at different depth imaging through the brain section. (**A, B**) Raw data of imaging immune-fluorescence-labeled amyloid-β fibrils in 200-μm-cut brain sections of 7.5-month-old 5XFAD female mouse. (**C**) Raw data of imaging Tom20 proteins in COS-7 cells using SMLM through unlabeled mouse brain section. (**D, E, F**) Raw data of imaging Thy1-ChR2-EYFP using SMLM in mouse brain section. The results shown are representative of 5 trials.

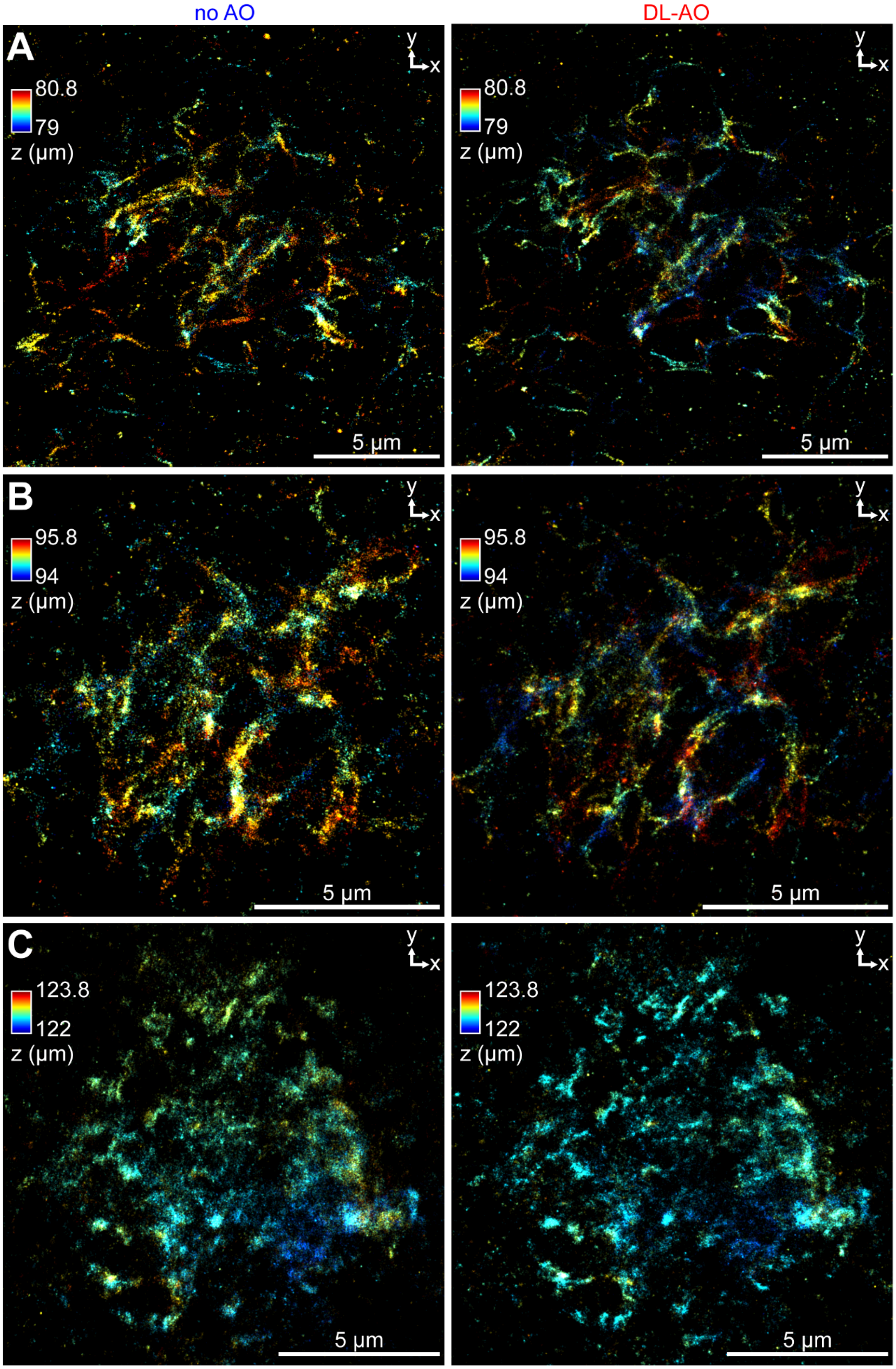
no AO
DL-AO
A
80.8
79
z (µm)
5 µm
B
95.8
94
z (µm)
5 µm
C
123.8
122
z (µm)
5 µm
y
x

Fig. SS23: 3D reconstructions of immune-fluorescence-labeled amyloid-β plaques in 125-μm-cut brain sections of 7.5-month-old 5XFAD female mice. (**A-C**) Comparisons between without and with DL-AO on amyloid-β plaques imaged using SMLM and reconstructed with in situ PSF model (INSPR) at 80 μm, 95 μm, and 123 μm from coverslip surface, respectively. Color code indicates axial positions of single molecule localizations. The imaged structures were found at depths near the axial limit of tissue thicknesses. Optically measured tissue thicknesses vary among samples, which might be caused by variations in media volume between the bottom and top coverslips

References

1. Descloux, A., Grußmayer, K. S. & Radenovic, A. Parameter-free image resolution estimation based on decorrelation analysis. *Nat Methods* **16**, 918–924 (2019).

2. Diederich, B., Then, P., Jugler, A., Forster, R. & Heintzmann, R. cellSTORM—Cost-effectivesuper-resolutionona cellphoneusingdSTORM. *PLoS One* **14**, e0209827 (2019).

3. Xu, F. *et al.* Three-dimensional nanoscopy of whole cells and tissues with in situ point spread function retrieval. *Nat Methods* **17**, 531–540 (2020).

4. Petrov, P. N. & Moerner, W. E. Addressing systematic errors in axial distance measurements in single-emitter localization microscopy. *Opt Express* **28**, 18616–18632 (2020).

5. LeCun, Y. *et al.* Backpropagation applied to digit recognition. *Neural computation* vol. 1 541–551 Preprint at (1989).

6. LeCun, Y., Bottou, L., Bengio, Y. & Haffner, P. Gradient-based learning applied to document recognition. *Proceedings of the IEEE* **86**, 2278–2323 (1998).

7. He, K., Zhang, X., Ren, S. & Sun, J. Deep residual learning for image recognition. in *Proceedings of the IEEE Computer Society Conference on Computer Vision and Pattern Recognition* vols 2016-Decem 770–778 (2016).

8. He, K., Zhang, X., Ren, S. & Sun, J. Delving deep into rectifiers: Surpassing human-level performance on imagenet classification. in *Proceedings of the IEEE International Conference on Computer Vision* vol. 2015 Inter 1026–1034 (2015).

9. Izeddin, I. *et al.* PSF shaping using adaptive optics for three-dimensional single-molecule super-resolution imaging and tracking. *Opt Express* **20**, 4957–4967 (2012).

10. Hanser, B. M., Gustafsson, M. G. L., Agard, D. A. & Sedat, J. W. Phase-retrieved pupil functions in wide-field fluorescence microscopy. *J Microsc* **216**, 32–48 (2004).

11. Liu, S., Kromann, E. B., Krueger, W. D., Bewersdorf, J. & Lidke, K. A. Three dimensional single molecule localization using a phase retrieved pupil function. *Opt Express* **21**, 29462–29487 (2013).

12. Petrov, P. N., Shechtman, Y. & Moerner, W. E. Measurement-based estimation of global pupil functions in 3D localization microscopy. *Opt Express* **25**, 7945–7959 (2017).

13. Mlodzianoski, M. J. *et al.* Active PSF shaping and adaptive optics enable volumetric localization microscopy through brain sections. *Nat Methods* **15**, 583–586 (2018).

14. Siemons, M. E., Hanemaaijer, N. A. K., Kole, M. H. P. & Kapitein, L. C. Robust adaptive optics for localization microscopy deep in complex tissue. *Nat Commun* **12**, 1–9 (2021).

15. Burke, D., Patton, B., Huang, F., Bewersdorf, J. & Booth, M. J. Adaptive optics correction of specimen-induced aberrations in single-molecule switching microscopy. *Optica* **2**, 177–185 (2015).

16. Tehrani, K. F., Xu, J., Zhang, Y., Shen, P. & Kner, P. Adaptive optics stochastic optical reconstruction microscopy (AO-STORM) using a genetic algorithm. *Opt Express* **23**, 13677–13692 (2015).

17. Tehrani, K. F., Zhang, Y., Shen, P. & Kner, P. Adaptive optics stochastic optical reconstruction microscopy (AO-STORM) by particle swarm optimization. *Biomed Opt Express* **8**, 5087–5097 (2017).

18. Rumelhart, D. E., Hinton, G. E. & Williams, R. J. Learning representations by back-propagating errors. *Nature* **323**, 533–536 (1986).

19. LeCun, Y. & Bengio, Y. Convolutional networks for images, speech and time-series. in *The Handbook of Brain Theory and Neural Networks* vol. 136 255–258 (MIT Press, 1995).

20. Möckl, L., Roy, A. R. & Moerner, W. E. Deep learning in single-molecule microscopy: fundamentals, caveats, and recent developments [Invited]. *Biomed Opt Express* **11**, 1633–1661 (2020).

21. Paine, S. W. & Fienup, J. R. Machine learning for improved image-based wavefront sensing. *Opt Lett* **43**, 1235–1238 (2018).

22. Zhang, P. *et al.* Analyzing complex single-molecule emission patterns with deep learning. *Nat Methods* **15**, 913–916 (2018).

23. Zelger, P. *et al.* Three-dimensional localization microscopy using deep learning. *Opt Express* **26**, 33166 (2018).

24. Möckl, L., Petrov, P. N. & Moerner, W. E. Accurate phase retrieval of complex 3D point spread functions with deep residual neural networks. *Appl Phys Lett* **115**, 1–5 (2019).

25. Saha, D. *et al.* Practical sensorless aberration estimation for 3D microscopy with deep learning. *Opt Express* **28**, 29044 (2020).

26. Smith, C. S., Joseph, N., Rieger, B. & Lidke, K. A. Fast, single-molecule localization that achieves theoretically minimum uncertainty. *Nat Methods* **7**, 373–375 (2010).

27. Huang, F. *et al.* Video-rate nanoscopy using sCMOS camera–specific single-molecule localization algorithms. *Nat Methods* **10**, 653–658 (2013).

28. Wang, B. & Booth, M. J. Optimum deformable mirror modes for sensorless adaptive optics. *Opt Commun* **282**, 4467–4474 (2009).

29. Ioffe, S. & Szegedy, C. Batch normalization: Accelerating deep network training by reducing internal covariate shift. in *32nd International Conference on Machine Learning, ICML 2015* vol. 1 448–456 (2015).

30. Kay, S. M. *Fundamentals of statistical signal processing, volume I: estimation theory*. (Prentice Hall, 1993).

31. Wyant, J. C. & Creath, K. Basic Wavefront Aberration Theory for Optical Metrology. in *Applied Optics and Optical Engineering* vol. 11 2 (Academic Press, 1992).

32. Liu, S., Huh, H., Lee, S.-H. & Huang, F. Three-Dimensional Single-Molecule Localization Microscopy in Whole-Cell and Tissue Specimens. *Annu Rev Biomed Eng* **22**, 155–184 (2020).

33. Hu, L., Hu, S., Gong, W. & Si, K. Deep learning assisted Shack–Hartmann wavefront sensor for direct wavefront detection. *Opt Lett* **45**, 3741 (2020).

34. Kalman, R. E. A new approach to linear filtering and prediction problems. *Transaction of the ASME—Journal of Basic Engineering* **82**, 35–45 (1960).

35. Lu, G. *et al.* Deep kalman filtering network for video compression artifact reduction. *Lecture Notes in Computer Science (including subseries Lecture Notes in Artificial Intelligence and Lecture Notes in Bioinformatics)* **11218 LNCS**, 591–608 (2018).

36. Angeli, A., Desmet, W. & Naets, F. Deep learning of multibody minimal coordinates for state and input estimation with Kalman filtering. *Multibody Syst Dyn* **53**, 205–223 (2021).

37. Roy, S. K., Nicolson, A. & Paliwal, K. K. On supervised LPC estimation training targets for augmented Kalman filter-based speech enhancement. *Speech Commun* **142**, 49–60 (2022).

38. Quirin, S., Pavani, S. R. P. & Piestun, R. Optimal 3D single-molecule localization for superresolution microscopy with aberrations and engineered point spread functions. *Proc Natl Acad Sci U S A* **109**, 675–679 (2012).

39. McGorty, R., Schnitzbauer, J., Zhang, W. & Huang, B. Correction of depth-dependent aberrations in 3D single-molecule localization and super-resolution microscopy. *Opt Lett* **39**, 275–278 (2014).

40. Gerchberg, R. W. & Saxton, W. O. A practical algorithm for the determination of phase from image and diffraction plane pictures. *Optik (Stuttg)* **35**, 237–246 (1972).

41. Hanser, B. M., Gustafsson, M. G. L., Agard, D. A. & Sedat, J. W. Phase retrieval for high-numerical-aperture optical systems. *Opt Lett* **28**, 801–803 (2003).

42. Von Diezmann, A., Shechtman, Y. & Moerner, W. E. Three-Dimensional Localization of Single Molecules for Super-Resolution Imaging and Single-Particle Tracking. *Chemical Reviews* vol. 117 7244–7275 Preprint at https://doi.org/10.1021/acs.chemrev.6b00629 (2017).

43. Hell, S. W. & Wichmann, J. Breaking the diffraction resolution limit by stimulated emission: stimulated-emission-depletion fluorescence microscopy. *Opt Lett* **19**, 780–782 (1994).

44. Wu, Y. & Shroff, H. Faster, sharper, and deeper: structured illumination microscopy for biological imaging. *Nat Methods* **15**, 1011–1019 (2018).

45. Betzig, E. *et al.* Imaging intracellular fluorescent proteins at nanometer resolution. *Science* **313**, 1642–1645 (2006).

46. Hess, S. T., Gould, Travis J Gunewardene, Mudalige Bewersdorf, J. & Mason, M. D. Ultrahigh resolution imaging of biomolecules by fluorescence photoactivation localization microscopy. *Methods Mol Biol* **544**, 483–522 (2009).

47. Rust, M. J., Bates, M. & Zhuang, X. Sub-diffraction-limit imaging by stochastic optical reconstruction microscopy (STORM). *Nat Methods* **3**, 793–795 (2006).

48. Moerner, W. E., Shechtman, Y. & Wang, Q. Single-molecule spectroscopy and imaging over the decades. *Faraday Discuss* **184**, 9–36 (2015).

49. Wegel, E. *et al.* Imaging cellular structures in super- resolution with SIM, STED and Localisation Microscopy: A practical comparison. (2016) doi:10.1038/srep27290.

50. Valli, J. *et al.* Seeing beyond the limit: A guide to choosing the right super-resolution microscopy technique. *Journal of Biological Chemistry* **297**, 100791 (2021).
