# Supplementary material for: Deep learning-driven adaptive optics for single-molecule localization microscopy
Source: Nat Methods. 2023 Sep 28;20(11):1748–58. doi: 10.1038/s41592-023-02029-0 (PMC10630144; doi:10.1038/s41592-023-02029-0)
Supplement: Supplementary file 2 — Reporting Summary [file 41592_2023_2029_MOESM2_ESM.pdf]

nature portfolio

Corresponding author(s): Fang Huang, Alexander A. Chubykin, Gary E. Landreth

Last updated by author(s): Jul 24, 2023

Reporting Summary

Nature Portfolio wishes to improve the reproducibility of the work that we publish. This form provides structure for consistency and transparency in reporting. For further information on Nature Portfolio policies, see our Editorial Policies and the Editorial Policy Checklist.

Statistics

For all statistical analyses, confirm that the following items are present in the figure legend, table legend, main text, or Methods section.

n/a	Confirmed	
☐	☒	The exact sample size (n) for each experimental group/condition, given as a discrete number and unit of measurement
☐	☒	A statement on whether measurements were taken from distinct samples or whether the same sample was measured repeatedly
☒	☐	The statistical test(s) used AND whether they are one- or two-sided<br>*Only common tests should be described solely by name; describe more complex techniques in the Methods section.*
☒	☐	A description of all covariates tested
☒	☐	A description of any assumptions or corrections, such as tests of normality and adjustment for multiple comparisons
☐	☒	A full description of the statistical parameters including central tendency (e.g. means) or other basic estimates (e.g. regression coefficient) AND variation (e.g. standard deviation) or associated estimates of uncertainty (e.g. confidence intervals)
☒	☐	For null hypothesis testing, the test statistic (e.g. F, t, r) with confidence intervals, effect sizes, degrees of freedom and P value noted<br>*Give P values as exact values whenever suitable.*
☒	☐	For Bayesian analysis, information on the choice of priors and Markov chain Monte Carlo settings
☒	☐	For hierarchical and complex designs, identification of the appropriate level for tests and full reporting of outcomes
☒	☐	Estimates of effect sizes (e.g. Cohen's d, Pearson's r), indicating how they were calculated

Our web collection on statistics for biologists contains articles on many of the points above.

Software and code

Policy information about availability of computer code

Data collection	Data was collected on a custom-built SMLM microscope which is described in Online Methods. The microscope is controlled by custom-made controlling software written in LabVIEW 2015 (National Instruments). The program is available from the corresponding author upon request.
Data analysis	Data analysis was performed using custom single molecule localization algorithm in MATLAB R2020a (MathWorks) which was shared in 'INSPR toolbox' available at https://github.com/HuanglabPurdue/INSPR. The in situ PSF model was obtained with INSPR toolbox, the detailed parameter of which was described in Supplementary Notes. The in vitro model was obtained using phase retrieval algorithm, which was described in Supplementary Notes and was shared at: https://github.com/HuanglabPurdue/smNet. The training data generation process used in this study was written in MATLAB R2020a (MathWorks), which is shared in supplementary software. The training and testing scripts used in this study was written in Python3.6.9 with Pytorch0.4.0 and CUDA10.1, which is shared in supplementary software with example training and testing data. The supplementary software for reproducing this study as well as its future updates will be available at GitHub: https://github.com/HuanglabPurdue/DL-AO. Additional datasets are available from the corresponding authors upon request.

For manuscripts utilizing custom algorithms or software that are central to the research but not yet described in published literature, software must be made available to editors and reviewers. We strongly encourage code deposition in a community repository (e.g. GitHub). See the Nature Portfolio guidelines for submitting code & software for further information.

Data

Policy information about availability of data

All manuscripts must include a data availability statement. This statement should provide the following information, where applicable:

- Accession codes, unique identifiers, or web links for publicly available datasets
- A description of any restrictions on data availability
- For clinical datasets or third party data, please ensure that the statement adheres to our policy

The results of molecular localizations for cell/tissue structures are available in Figshare, doi: 10.6084/m9.figshare.23823438. Example training and testing data for DL-AO are available in supplementary software packages. Complete training and testing datasets can be generated through the shared codes. Other data that support the findings of this study are available from the corresponding authors upon request.

Human research participants

Policy information about studies involving human research participants and Sex and Gender in Research.

Reporting on sex and gender	N/A
Population characteristics	N/A
Recruitment	N/A
Ethics oversight	N/A

Note that full information on the approval of the study protocol must also be provided in the manuscript.

Field-specific reporting

Please select the one below that is the best fit for your research. If you are not sure, read the appropriate sections before making your selection.

☒ Life sciences ☐ Behavioural & social sciences ☐ Ecological, evolutionary & environmental sciences

For a reference copy of the document with all sections, see nature.com/documents/nr-reporting-summary-flat.pdf

Life sciences study design

All studies must disclose on these points even when the disclosure is negative.

Sample size	For biological data, the number of sub-regions analyzed is determined by the number of emission events obtained from single molecule switching nanoscopy experiments. Number of localizations per dataset is listed in Supplementary Table 1. For robustness test, the sample size was determined by the number of experiments performed using our technique. The number of repeated experiments was labeled in figures or figure captions. For simulated data, the sample size was determined by the simulated emission patterns of single molecules for different imaging conditions.
Data exclusions	Qualitative exclusion criteria for accepting or rejecting imaged samples were pre-established based on comparisons to previously published images and preliminary experiments. Single molecule localizations were statistically tested and rejected/accepted based on their log-likelihood ratio (as goodness of fit metric), theoretical uncertainty, emitted photon as well as their convergence during fitting.
Replication	All attempts at replication were successful. The number of replications were provided in the figure legends.
Randomization	In this study, there is no allocation of sample into groups.
Blinding	In this study, there is no allocation of sample into groups.

Reporting for specific materials, systems and methods

We require information from authors about some types of materials, experimental systems and methods used in many studies. Here, indicate whether each material, system or method listed is relevant to your study. If you are not sure if a list item applies to your research, read the appropriate section before selecting a response.

Materials & experimental systems

n/a	Involved in the study
☐	☒ Antibodies
☐	☒ Eukaryotic cell lines
☒	☐ Palaeontology and archaeology
☐	☒ Animals and other organisms
☒	☐ Clinical data
☒	☐ Dual use research of concern

Methods

n/a	Involved in the study
☒	☐ ChIP-seq
☒	☐ Flow cytometry
☒	☐ MRI-based neuroimaging

Antibodies

Antibodies used	Primary antibodies:<br>Tom20 antibody (Santa Cruz Biotechnology, Cat#sc-11415, used at 1:500)<br>β-Amyloid antibody (Cell Signaling Technology, Cat#2454, used at 1:1000)<br>Anti-GFP antibody (Abcam, Cat#ab13970, used at 1:1000)<br><br>Secondary antibodies:<br>Goat anti-rabbit IgG (H+L) highly cross-adsorbed secondary antibody, Alexa Fluor 647 (Invitrogen, Cat#A21245, used at 1:500)<br>Donkey anti-rabbit IgG (H+L) highly cross-adsorbed secondary antibody, Alexa Fluor 647 (Invitrogen, Cat#A31573, used at 1:1000)<br>Goat anti-chicken IgY (H+L) secondary antibody, Alexa Fluor 647 (Invitrogen, Cat#A21449, used at 1:600)
Validation	All the antibodies used here are commercial products. They have been extensively used by us and others in the past, and have been validated through our previous experiments as well as the manufacturer's own test and validations. Below we list one reference for each antibody:<br>Tom20 antibody - doi: 10.1016/j.cell/2016/06/016<br>β-Amyloid antibody - doi: 10.1038/s41592-018-0053-8<br>Anti-GFP antibody - doi: 10.1038/s41467-018-08146-1<br>Goat anti-rabbit IgG (H+L) highly cross-adsorbed secondary antibody, Alexa Fluor 647 - doi: 10.1016/j.cell/2016/06/016<br>Donkey anti-rabbit IgG (H+L) highly cross-adsorbed secondary antibody, Alexa Fluor 647 - doi: 10.1016/j.cell/2016/06/016<br>Goat anti-chicken IgY (H+L) secondary antibody, Alexa Fluor 647 - doi: 10.1016/j.stem.2016.11.019

Eukaryotic cell lines

Policy information about cell lines and Sex and Gender in Research

Cell line source(s)	COS-7 cells (CRL-1651 from ATCC)
Authentication	Cell line was purchased from ATCC and was not independently authenticated.
Mycoplasma contamination	Not applicable.
Commonly misidentified lines<br>(See ICLAC register)	No commonly misidentified cell lines were used.

Animals and other research organisms

Policy information about studies involving animals; ARRIVE guidelines recommended for reporting animal research, and Sex and Gender in Research

Laboratory animals	For amyloid β plaques in mouse brains, 5xFAD mice were reported previously (PMID: 17021169). The mouse strain #: 000664. Briefly, this transgenic mouse coexpress five FAD mutations [APP K670N/M671L (Swedish) + I716V (Florida) + V717I (London) and PS1 M146L + L286V], introduced into APP and PS1 cDNAs by site-directed mutagenesis, and then subcloned into exon 2 of the mouse Thy1 cassette (PMID: 17021169). In this study, female 7.5-month-old mice were used and weighed between 20-30g. The mice were housed with 40-60% humidity at 20-26 °C.<br>For dendrites of neurons in the mouse primary visual cortex, male postnatal day 89 and 273 Ai32 mice were used (Jackson Lab, stock#012569). Briefly, the mice express ChR2-eYFP following exposure to Cre-recombinase, allowing for conditional optogenetic activation. The mice were housed with 44% humidity at 22 °C. Mice strain#: 000664. Mice species: Mus musculus.
Wild animals	The study did not involve wild animals
Reporting on sex	This information has not been collected
Field-collected samples	The study did not involve samples collected from the field
Ethics oversight	All animal procedures associated with mice were approved by the Indiana University School of Medicine Institutional Animal Care and Use Committee (IACUC) and Purdue Animal Care and Use Committee (PACUC), and complied with all relevant ethical regulations.

Note that full information on the approval of the study protocol must also be provided in the manuscript.
