# Supplementary material for: Deep learning-driven adaptive optics for single-molecule localization microscopy
Source: Nat Methods. 2023 Sep 28;20(11):1748–58. doi: 10.1038/s41592-023-02029-0 (PMC10630144; doi:10.1038/s41592-023-02029-0)
Supplement: Supplementary file 16 — This software is distributed as accompanying software for this paper. [file 41592_2023_2029_MOESM16_ESM.zip › Software/DL-AO user manual.pdf]

Example code for deep learning driven adaptive optics ('DL-AO')

This software is distributed as accompanying software for the manuscript: 'Deep Learning Driven Adaptive Optics for Single Molecule Localization Microscopy' by Peiyi Zhang, Donghan Ma, Xi Cheng, Andy P. Tsai, Yu Tang, Hao-Cheng Gao, Li Fang, Cheng Bi, Gary E. Landreth, Alexander A. Chubykin and Fang Huang

1. Files included in this package

1.1 Content of 'Training Data Generation

Example Data:

MirrorMode.mat:	Measured mirror deformation modes used for DL-AO
SystemPupil.mat:	Measured pupil phase and magnitude under instrument optimum

Matlab scripts:

helpers\ PSF_MM.m:	Simulation of PSFs with optical aberration
helpers\ OTFrescale.m:	OTF rescaling of the simulated PSFs
helpers\ Net1Filter.m:	Function for selecting detectable PSFs
helpers\ filterSub.m:	Function for identifying pixels containing local maximum intensities
main.m:	Main script for generating training dataset

1.2 Content of 'Training and testing for DL-AO'

Example Data:

data.mat:	A small training dataset containing simulated PSFs.
label.mat:	Labels of the training dataset
testdata.mat	A small test dataset
testlabel.mat	Underlying true positions to compare estimation results
Network1.pth	Trained model1 for DL-AO
Network2.pth	Trained model2 for DL-AO
Network3.pth	Trained model3 for DL-AO

Python scripts:

main.py: Main script for training neural network

model.py: Script for neural network architecture definition

opts.py: Definitions of user-adjustable variables

test.py: Script for testing training result

1.3 Content of 'DL-AO_Inference_Demo_Colab'

Google Colaboratory (i.e. Colab) Notebook:

DL-AOInferenceDemo.ipynb: Script for testing training results in Colab

Python scripts (Modified from scripts in **1.2** for execution in Colab):

model.py: Script for neural network architecture definition

opts.py: Definitions of user-adjustable variables

Note:

1. Example data only showed 1000 sub-regions. Training for DL-AO used 6 million images. Additional training and validation datasets are available upon request.

2. Network1-3 are three models with different training ranges used in DL-AO. The detailed training range sees Supplementary Table 4.

3. Before running Jupyter Notebook 'DL-AOInferenceDemo.ipynb' in Colab, create an 'ExampleData' folder, then copy 'MirrorMode.mat', 'testdata.mat', 'testlabel.mat', 'Network1.pth', 'Network2.pth', 'Network3.pth' from the other two folders (**1.1** and **1.2)** into this 'ExampleData' folder in 'DL-AO_Inference_Demo_Colab' (see **'Instruction for Testing Network in Web Browser.pdf'** for details)

2. Instructions on generating training dataset

Change MATLAB current folder to the directory that contains main.m. Then run main.m to generate the training dataset. (uncomment Line 90 when generating training data for Net1)

This code has been tested on the following system and packages:

Microsoft Windows 10 Education, Matlab R2020a, DIPimage 2.8.1 (http://www.diplib.org/).

3. Instructions on training and testing neural networks for DL-AO

The code has been tested on the following system and packages:

Ubuntu16.04LTS, Python3.6.9, Pytorch0.4.0, CUDA10.1, MatlabR2015a

1. To start training, type the following command in the terminal:

python main.py --datapath ./ExampleData --save ./Models

The expected output and runtime with the small example training dataset is shown below:

```
loading from:  ./ExampleData
training data size: 990 x 2 x 32 x 32
training label size: 990 x 28
validation data size: 10 x 2 x 32 x 32
validation label size: 10 x 28

epoch: 1
Training (# of batches):: 100%|| 8/8 [00:12<00:00,  1.07s/it]
training error: 0.6630098447203636  (A.U.)
Validating (# of batches):: 100%|| 1/1 [00:00<00:00, 25.39it/s]
validation error: inf  (A.U.)

epoch: 2
Training (# of batches):: 100%|| 8/8 [00:01<00:00,  7.41it/s]
training error: 0.5740059688687325  (A.U.)
Validating (# of batches):: 100%|| 1/1 [00:00<00:00, 28.26it/s]
validation error: nan  (A.U.)
```

Due to insufficient training data included in ‘ExampleData’, the validation error is inf. More training datasets can be generated with the Matlab code described in the Section 2. An example output with 100 times more training data is shown below:

```
loading from:  ./ExampleData
training data size: 99000 x 2 x 32 x 32
training label size: 99000 x 28
validation data size: 1000 x 2 x 32 x 32
validation label size: 1000 x 28

epoch: 1
Training (# of batches):: 100%|| 774/774 [01:56<00:00,  7.34it/s]
training error: 0.3818694018886379  (A.U.)
Validating (# of batches):: 100%|| 8/8 [00:00<00:00, 25.27it/s]
validation error: 0.3765249215066433  (A.U.)

epoch: 2
Training (# of batches):: 100%|| 774/774 [01:49<00:00,  7.10it/s]
training error: 0.3573831679439052  (A.U.)
Validating (# of batches):: 100%|| 8/8 [00:00<00:00, 24.57it/s]
validation error: 0.3540059588495445  (A.U.)
```

2. To test this, type the following command in the terminal:

python test.py --datapath ./ExampleData/ --save ./result –checkptname ./ExampleData/Network2

The expected output and runtime with the small testing dataset are shown below:

```
test data size: 1000 x 2 x 32 x 32
Testing (# of batches): 100%|| 8/8 [00:11<00:00,  1.41s/it]
result saved at:  ./result
```

Note:

1. Each iteration will save a model named by the iteration number in folder './Models/'
2. The user could open errorplot.png in the folder './Models/' to observe the evolution of training and validation errors.
3. The user-adjustable variables for training will be saved in '/Models/opt.txt'
4. The training and validation errors for each iteration will be saved in 'Models/error.log' (The 1st column is the training error and the 2nd column is the validation error)
5. Pytorch Installation Instruction and typical installation time see https://pytorch.org/get-started/locally/.
