# Supplementary material for: Deep learning-driven adaptive optics for single-molecule localization microscopy
Source: Nat Methods. 2023 Sep 28;20(11):1748–58. doi: 10.1038/s41592-023-02029-0 (PMC10630144; doi:10.1038/s41592-023-02029-0)
Supplement: Supplementary file 16 — This software is distributed as accompanying software for this paper. [file 41592_2023_2029_MOESM16_ESM.zip › Software/Instruction for Testing Network in Web Browser.pdf]

Instruction for Testing Network in Web Browser

This instruction is distributed with accompanying software for the manuscript: 'Deep Learning Driven Adaptive Optics for Single Molecule Localization Microscopy' by Peiyi Zhang, Donghan Ma, Xi Cheng, Andy P. Tsai, Yu Tang, Hao-Cheng Gao, Li Fang, Cheng Bi, Gary E. Landreth, Alexander A. Chubykin and Fang Huang

1. Files included in 'DL-AO_Inference_Demo_Colab':

Google Colaboratory (i.e. Colab) Notebook:

DL-AOInferenceDemo.ipynb: Script for testing training results in Colab

Python scripts:

model.py:	Script for neural network architecture definition
opts.py:	Definitions of user-adjustable variables

Note:

ExampleData is required for running the Jupyter Notebook. Before running 'DL-AOInferenceDemo.ipynb', you need to copy the following data into this folder:

1. MirrorMode.mat (in Software\Training Data Generation\ExampleData)
2. testdata.mat (in Software\Training and testing for DL-AO\ExampleData)
3. teslabel.mat (in Software\Training and testing for DL-AO\ExampleData)
4. Network1.pth (in Software\Training and testing for DL-AO\ExampleData)
5. Network2.pth (in Software\Training and testing for DL-AO\ExampleData)
6. Network3.pth (in Software\Training and testing for DL-AO\ExampleData)

2. Instructions on testing the trained DL-AO network in Web Browser

This code has been tested in Google Chrome Browser on Google Colaboratory (Colab, https://colab.research.google.com/), which provides free access to essential computing hardware such as GPUs or TPUs used in DL-AO. Besides, all the packages essential for testing DL-AO are pre-installed in Colab. Therefore, no installation processes are required on your local PC.

To run the DL-AO network inference:

1. Open https://drive.google.com/drive/my-drive in Google Chrome Browser and log in with your Gmail account.

2. Drag the entire ‘DL-AO_Inference_Demo_Colab’ folder into the ‘My Drive’ folder in your Google Drive.
3. Open ‘DL-AO_Inference_Demo_Colab’ folder in your Google Drive and you should see the files as follows:

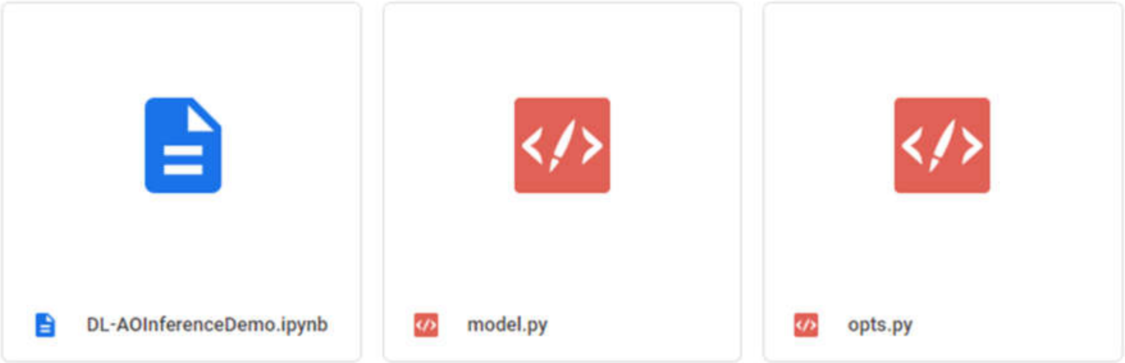

4. Right-click the Jupyter Notebook named ‘DL-AOInferenceDemo.ipynb’, then select “Google Colaboratory” to open the Notebook in Colab

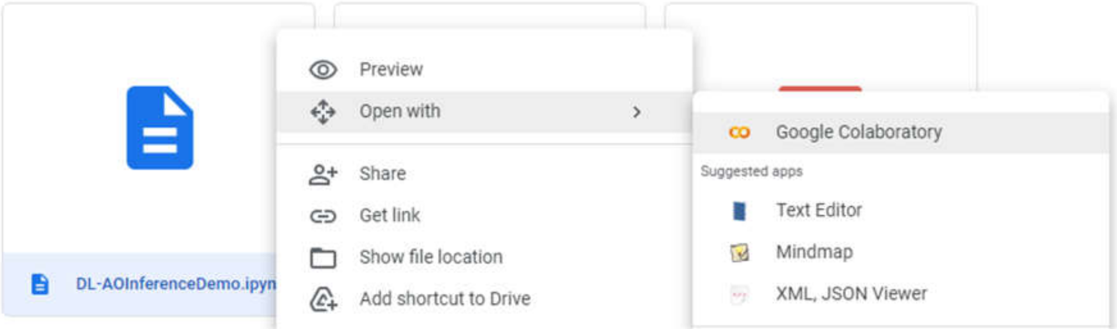

5. To enable GPU in your notebook, click "Edit", choose "Notebook Setting", then select “GPU” as Hardware Accelerator in “Notebook Setting”

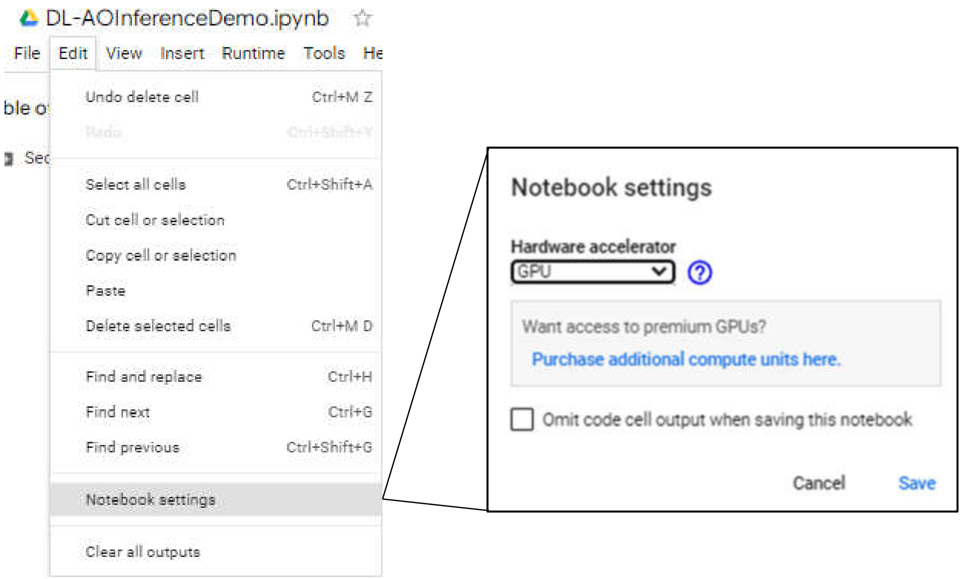

6. Run the Code Cell in 'DL-AOInferenceDemo.ipynb' by clicking the "run" button in the top left corner. There are two Code Cells in the 'DL-AOInferenceDemo.ipynb' Notebook. The first one is for testing the DL-AO network, and the second one is for displaying the test result. An example output of these Code Cells is attached as follows

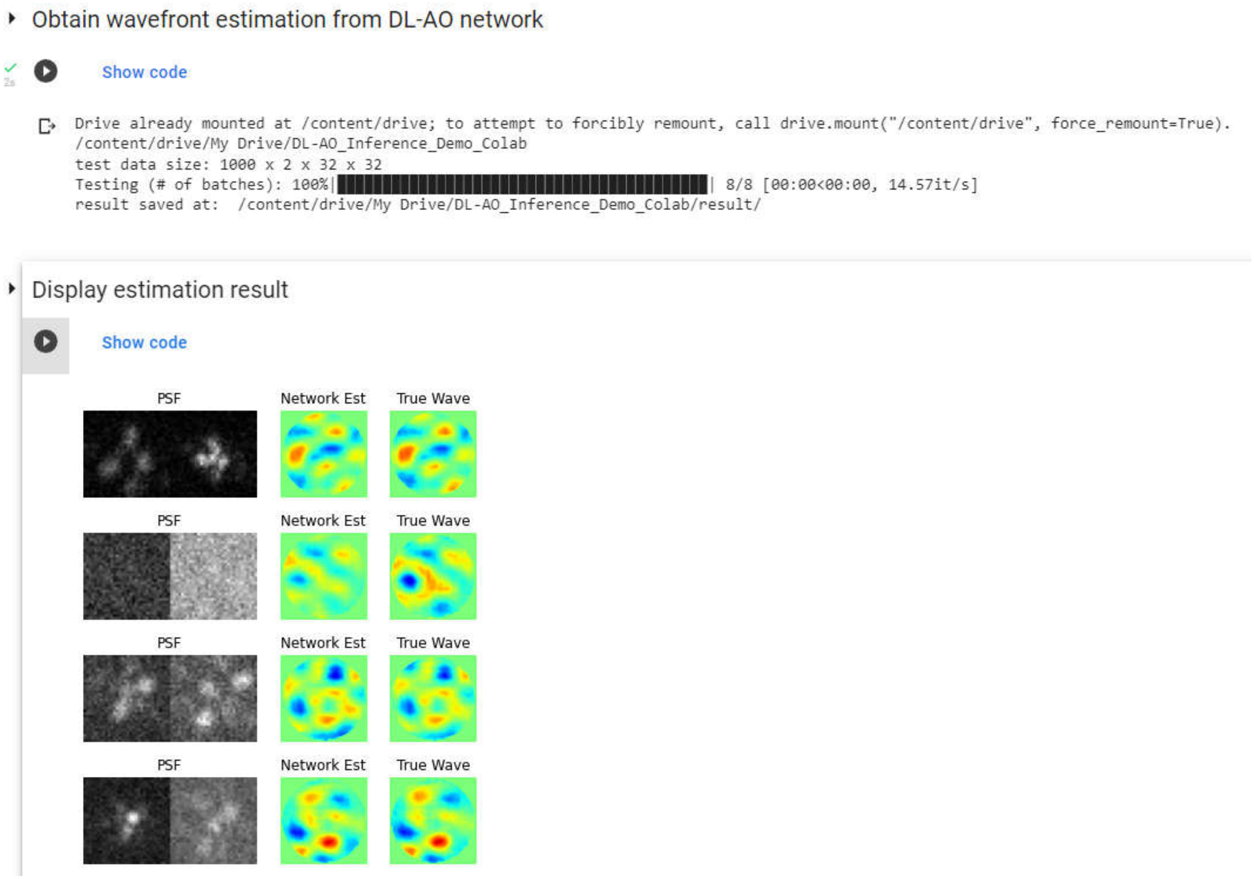

3. Step-by-Step explanation on code in ‘DL-AOInferenceDemo.ipynb’

In this section, we write a detailed explanation of code in the first Code Cell in this Notebook. We re-arranged the code into multiple smaller Code Cells. The code in each Code Cell (grey areas), and the expected outputs (white areas) are attached below:

(1) Input modules required for running this Notebook

```
# import modules
import torch
import torch.nn as nn
import torch.nn.parallel
import os
import sys
import scipy.io as sio
import numpy as np
from torch.autograd import Variable
import argparse
from tqdm import tqdm
import h5py
from google.colab import drive
```

(2) Verify if GPU is enabled in your notebook

```
# verify if GPU is enabled (Output "True" indicates GPU is available now)
torch.cuda.is_available()
```

```
True
```

(3) Connect Google Drive with Colab to access Python Scripts and data in Google Drive (You need to grant access to this notebook to access your Google Drive following the instructions from the website)

```
# connect Google Drive into Colab
drive.mount('/content/drive')
```

```
Mounted at /content/drive
```

(4) Change the directory to access files in the ‘DL-AO_Inference_Demo_Colab’ folder in Google Drive

```
basedir = '/content/drive/My Drive/DL-AO_Inference_Demo_Colab/'
os.chdir(basedir)                                               # change directory
!pwd                                                            # check current directory
```

```
/content/drive/My Drive/DL-AO_Inference_Demo_Colab
```

(5) Execute the opts.py script in your Google Drive. This script contains definitions of user-adjustable variables.

```
execfile('opts.py')                         # executes the contents of opts.py
opt = options()                             # access the function in opts.py
opt.datapath = basedir + 'ExampleData/'     # set path for loading test data
```

(6) Load test data as follows

```
#load test data
def loaddata(opt):                                                                                          # function definition
    mat_contents = h5py.File(os.path.join(opt.datapath, 'testdata.mat'),'r')                               # read testdata file
    dataset = torch.from_numpy(np.array(mat_contents.get('imsp'))).float()                                 # load variable contains PSFs in test data
    if dataset.dim() < 4:                                                                                  # check test data format
        print('(Error) require 4D input. \Plese modify your input to be: imsz x imsz x nchannel x datasize')  # error message
        exit()

    print('test data size:', dataset.size(0), 'x', dataset.size(1), 'x', dataset.size(2), 'x', dataset.size(3)) # print data dimension

    for i in range(data.size(0)):
        for j in range(opt.channel):
            dataset[i][j]=dataset[i][j]/torch.max(dataset[i][j])                                           # normalize sub-regions

    return dataset

data = loaddata(opt)                                                                                       # load testdata
```

```
test data size: 1000 x 2 x 32 x 32
```

(7) Load the trained DL-AO network

```
execfile('model.py')
opt.datapath = basedir + 'ExampleData/'                     # set path for loading test data
opt.checkptname = 'Network2.pth';                           # set name of pre-trained DL-AO network
model = torch.load(os.path.join(opt.datapath, opt.checkptname))  # Load the trained DL-AO network
model = model.module.to(device='cuda:0')                    # Specify hardware
```

(8) Test the DL-AO network.

```
def test(model, opt, data):
    count, totalErr, err = 0, 0, 0
    opt.datasize = data.size(0)                                                            # number of sub-regions
    opt.imWidth = data.size(2)                                                             # image width of sub-regions
    opt.imHeight = data.size(3)                                                            # image height of sub-regions
    if opt.datasize<opt.batchsize:
      opt.batchsize = opt.datasize                                                         # batch size for testing
    x = torch.Tensor(opt.batchsize, opt.channel, opt.imWidth, opt.imHeight).cuda()         # Tensor for loading sub-regions
    model.eval()                                                                           # set model in evaluation (inference) mode
    output = torch.Tensor()                                                                # Tensor for concatenating output
    with torch.no_grad():                                                                  # deactivate autograd engine
        for i in tqdm(range(0, opt.datasize, opt.batchsize), ncols = 100, desc = "Testing (# of batches)"):  # loop through batches with progress bars
            for ii in range(opt.batchsize):                                                # loop sub-regions in one batch
                if (i+ii) > opt.datasize - 1:
                    break
                else:
                    x[ii] = data[i+ii]
            out = model.forward(x)                                                         # forward propagation
            output = torch.cat([output, out.cpu()], 0)                                     # concatenating outputs

    savepath = basedir + 'result/';                                                        # path for saving results
    if not os.path.exists(savepath):
        os.makedirs(savepath)                                                              # create folder for saving results
    sio.savemat(os.path.join(savepath, 'result.mat'), {'aberration': output[0:opt.datasize].numpy()})  # save result
    print('\nresult saved at: ', savepath)

test(model, opt, data)                                                                     # Test network
```

```
Testing (# of batches): 100%|████████████████████████████████████████| 8/8 [00:00<00:00, 14.23it/s]
result saved at:  /content/drive/My Drive/DL-AO_Inference_Demo_Colab/result/
```

(9) After step 8, there will be a result.mat file generated in the result folder (see the output of step 8). Now click “run” on the “Display test result” Code Cell to visualize the estimation result from

a trained DL-AO network on every single bi-plane sub-region. An example output is shown as follows:

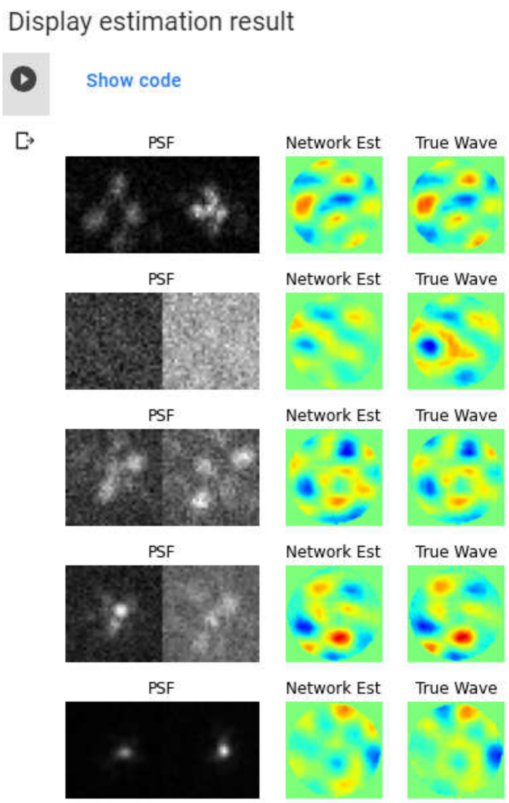

Note:

We compare the estimation with the ground truth using wavefront shape, instead of mirror mode coefficients. This is because one wavefront shape can correspond to different mirror mode coefficients due to the coupling between experimental mirror deformation modes.
